# Supplementary material for: Localized transmission of an aquatic pathogen drives hidden epidemics and population collapse in a terrestrial host
Source: Nat Ecol Evol. 2026 Jan 2;10(2):308–17. doi: 10.1038/s41559-025-02930-1 (PMC12890588; doi:10.1038/s41559-025-02930-1)
Supplement: Supplementary file 1 — Supplementary Text, Figs. 1–48 and Tables 1–11. [file 41559_2025_2930_MOESM1_ESM.pdf]

nature ecology & evolution

Supplementary information https://doi.org/10.1038/s41559-025-02930-1

# Localized transmission of an aquatic pathogen drives hidden epidemics and population collapse in a terrestrial host

In the format provided by the authors and unedited

# Contents

**Lifestyle and Bd research** **1**

**Bayesian spatial multistate capture-recapture model** **5**

**Prevalence and spatial clustering of Bd infection** **11**

**Environmental variables** **13**

**Spatial point pattern analysis** **14**

**Individual-based model** **17**

**Role of syntopic amphibians in Bd transmission** **25**

**Probability of dispersal** **28**

**Bacteriome** **30**

**Strength of evidence** **31**

**References** **32**

**Supplementary Figures** **36**

**Supplementary Tables** **84**

# Supplementary Information: Lifestyle and Bd Research

## Introduction

Amphibians exhibit a remarkable diversity of life histories, with at least 74 distinct reproductive modes identified to date (Nunes-de-Almeida et al. 2021). Despite this extraordinary diversity, amphibians are frequently assumed to be intrinsically linked to aquatic environments such as streams, ponds, or wetlands. This perception is mirrored in the research focus on chytridiomycosis, the disease caused by the fungal pathogen *Batrachochytrium dendrobatidis* (Bd), which has disproportionately concentrated on species that are aquatic during at least part of their life cycle.

However, a substantial number of amphibian species are fully terrestrial, lacking any aquatic larval stage and completing their entire life cycle on land. To provide quantitative support for this observation and evaluate potential Bd research biases, we carried out two complementary analyses: (1) we quantified the global prevalence of fully terrestrial amphibians using species-level data from the IUCN Red List; and (2) we conducted a systematic literature review to assess the lifestyle of focal species in field-based studies investigating the individual- or population-level impacts of Bd in wild amphibian populations.

## Classification of amphibians by lifestyle

Using the IUCN Red List API (https://iucn-uk.github.io/iucnredlist/), accessed via the `iucnredlist` R package (Chamberlain 2023), we classified amphibians into two broad lifestyle categories: *fully terrestrial* and *aquatic*. Specifically, a species was classified as aquatic if any of the following IUCN habitat types (see the Red List's Habitat Classification Scheme for further details, https://www.iucnredlist.org/resources/habitat-classification-scheme) were listed in its Red List assessment:

- Forest – Subtropical/Tropical Swamp
- Wetlands (inland) – Bogs, Marshes, Swamps, Fens, Peatlands
- Wetlands (inland) – Permanent Freshwater Marshes/Pools (under 8ha)
- Wetlands (inland) – Seasonal/Intermittent Freshwater Marshes/Pools (under 8ha)

- Wetlands (inland) – Permanent Rivers/Streams/Creeks (includes waterfalls)
- Wetlands (inland) – Seasonal/Intermittent Freshwater Lakes (over 8ha)
- Wetlands (inland) – Permanent Freshwater Lakes (over 8ha)
- Wetlands (inland) – Seasonal/Intermittent/Irregular Rivers/Streams/Creeks
- Artificial/Aquatic – Excavations (open)
- Artificial/Aquatic – Irrigated Land (includes irrigation channels)
- Artificial/Aquatic – Ponds (below 8ha)
- Wetlands (inland) – Freshwater Springs and Oases
- Artificial/Aquatic – Canals and Drainage Channels, Ditches
- Artificial/Aquatic – Water Storage Areas (over 8ha)
- Artificial/Aquatic – Aquaculture Ponds
- Artificial/Aquatic – Seasonally Flooded Agricultural Land
- Artificial/Aquatic – Karst and Other Subterranean Hydrological Systems (human-made)
- Marine Coastal/Supratidal – Coastal Brackish/Saline Lagoons/Marine Lakes
- Wetlands (inland) – Permanent Saline, Brackish or Alkaline Marshes/Pools
- Wetlands (inland) – Shrub Dominated Wetlands
- Wetlands (inland) – Karst and Other Subterranean Hydrological Systems (inland)
- Marine Coastal/Supratidal – Coastal Caves/Karst
- Wetlands (inland) – Alpine Wetlands (includes temporary waters from snowmelt)
- Artificial/Aquatic – Wastewater Treatment Areas
- Marine Neritic – Estuaries
- Marine Intertidal – Salt Marshes (Emergent Grasses)
- Wetlands (inland) – Geothermal Wetlands
- Wetlands (inland) – Seasonal/Intermittent Saline, Brackish or Alkaline Marshes/Pools
- Wetlands (inland) – Permanent Inland Deltas

- Wetlands (inland) – Tundra Wetlands (incl. pools and temporary waters from snowmelt)
- Wetlands (inland) – Permanent Saline, Brackish or Alkaline Lakes
- Wetlands (inland) – Seasonal/Intermittent Saline, Brackish or Alkaline Lakes and Flats
- Marine Intertidal – Sandy Shoreline and/or Beaches, Sand Bars, Spits, etc.

If none of these habitats were listed, the species was classified as fully terrestrial.

Therefore, we defined species as aquatic if they were associated with aquatic habitats during any part of their life cycle. This broad category includes stream-breeding, pond-breeding, and fully aquatic species. Conversely, we expect that species classified as fully terrestrial are mostly those that lack an aquatic larval stage, including most direct-developing species and those with terrestrial larvae. We expect many of these species to be forest specialists (Müller et al. 2013), as is the case for *Rhinoderma darwinii*. The rationale behind this dichotomous classification is grounded in our expectation that Bd transmission dynamics are fundamentally different in species that do not utilise water bodies compared to those that do. While we acknowledge that this is a simplified framework—given that water dependence can vary even within species—we argue that it provides a valuable starting point for assessing the prevalence of fully terrestrial lifestyles and their representation in Bd research.

From a total of 8,009 amphibian species assessed by the IUCN Red List, 2,720 (34%) were classified as fully terrestrial. We regard this estimate as conservative, as we classified species as aquatic if they were associated with any of the habitat types above, irrespective of the reported habitat suitability. Thus, some species may have been classified as aquatic even if the habitat in question was only marginal or of low relevance to their actual ecology.

We examined the distribution of lifestyles across taxonomic orders, whether Bd was listed in the `ias` (invasive alien species) field of the IUCN threats module (a free-text field reporting threats from invasive pathogens, animals, or plants), as well as biogeographical realms and extinction risk categories (Supplementary Fig. 10).

Our analysis revealed that fully terrestrial amphibians occur in all amphibian orders, but are particularly prevalent in Caudata and Gymnophiona. Bd is commonly listed as a threat to fully terrestrial species in every order; notably in nearly 75% of urodeles, despite the surprisingly limited number of field-based Bd impact studies for this group (see below). Fully terrestrial amphibians are especially common in the Neotropical and Australasian realms—regions where Bd-related declines were first documented—, and are less common in the Palearctic.

In terms of conservation status, a high number of fully terrestrial species are in threatened categories. Notably, over 50% of species listed as Extinct are fully terrestrial. This finding aligns with previous studies showing that amphibians with terrestrial larvae are

particularly sensitive to habitat modification (*50*), which remains the leading threat to amphibians globally.

## Evaluation of field-based Bd impact studies

To quantify the scope of field studies that have assessed the effects of Bd infection at the individual or population level in the wild, we conducted a structured literature search using Web of Science. We used the following search terms:

> `"batrachochytrium" OR "chytridiomycosis"` AND
> `"capture-mark-release" OR "release-recapture" OR "unmarked" OR "capture-recapture" OR "capture-mark-recapture" OR "jolly-seber" OR "mark" OR "cormack-jolly-seber" OR "multi-state" OR "n-mixture" OR "hierarchical model" OR "state-space model" OR "integra* projection model" OR "integra* population model" OR "skeletochronology" OR "age-structure" OR "time-series" OR "time series" OR "demograph*" OR "longitudinal" OR "long-term" OR "multievent" OR "population model" OR "occupancy"`

We performed a search on the 23rd of March 2025 for the period 1998-2025, which returned 457 results. For comparison, a broader search using only "`batrachochytrium`" OR "`chytridiomycosis`" returned 2,752 articles, indicating that only a small fraction of Bd-related research incorporates population-level or longitudinal field data.

We read the abstracts of 457 publications. We excluded literature reviews, mesocosm or laboratory experiments, theoretical studies that did not use novel data but relied on estimates from previous research, studies that only focused on Bd infection prevalence or infection intensity, and studies conducted in Bd-free populations or those lacking a clear Bd detection methodology (e.g. a study might claim to evaluate host demography before and after Bd invasion but fail to provide evidence on how Bd presence in the studied population(s) was assessed). This resulted in 75 articles covering 49 amphibian species. Of these 49 species, only 8% (4 species) are fully terrestrial (Supplementary Fig. 11).

# Supplementary Information: Bayesian Spatial Multistate Capture-recapture Model

## Introduction

Our Bayesian spatial multistate capture-recapture model estimates the true survival probability and state-transition probabilities (in this case, infection-status transitions) of individuals within a defined study area using search-encounter capture-recapture data. It integrates the state-space formulation of the multistate capture-recapture model (as described in [Kéry and Schaub 2012]) with the movement submodel of the spatial Cormack-Jolly-Seber model from Schaub and Royle (2014). Specifically, our model consists of two interdependent state processes that evolve in a Markovian fashion, along with one observation process. The first state process models survival and infection dynamics, while the second state process models movement. The model is implemented in BUGS language in JAGS (Plummer 2003).

## Model description

### State transitions

We define a latent state variable $z_{i,t}$ to indicate the true Bd-infection and survival status of individual $i$ at time $t$:

- $z_{i,t} = 1$: Bd-negative and alive
- $z_{i,t} = 2$: Bd-positive and alive
- $z_{i,t} = 3$: Dead

The latent state process is described as follows:

$$\begin{aligned} z_{i,f_i} &= fs_i \\ z_{i,t+1} \mid z_{i,t} &\sim \text{Categorical}(\Omega_{z_{i,t},i,t,1\ldots S}) \end{aligned} \tag{1}$$

where $fs_i$ denotes the true initial state of individual $i$ at its first capture event $f$. In this model the true initial state is assumed to be observed without error. Note that the model

tracks the state (and observation) of individual $i$ only from $f_i$ onward. The transition between states follows a first-order Markov process, where the state at time $t+1$ depends only on the state at time $t$. Specifically, the latent state of individual $i$ in time $t + 1$ follows a categorical distribution, with probabilities defined by the four-dimensional state-transition matrix $\mathbf{\Omega}$ (denoted `ps` in the JAGS model implementation). This matrix defines the probability of transitioning from state $S(t)$ to state $S(t+1)$ as follows:

$$\begin{aligned}
\Omega_{1,i,t,1} &= \phi_A[i,t] \cdot (1 - \psi_{AB}[i,t]) \\
\Omega_{1,i,t,2} &= \phi_A[i,t] \cdot \psi_{AB}[i,t] \\
\Omega_{1,i,t,3} &= 1 - \phi_A[i,t] \\
\Omega_{2,i,t,1} &= \phi_B[i,t] \cdot \psi_{BA}[i,t] \\
\Omega_{2,i,t,2} &= \phi_B[i,t] \cdot (1 - \psi_{BA}[i,t]) \\
\Omega_{2,i,t,3} &= 1 - \phi_B[i,t] \\
\Omega_{3,i,t,1} &= 0 \\
\Omega_{3,i,t,2} &= 0 \\
\Omega_{3,i,t,3} &= 1
\end{aligned} \tag{2}$$

where $\phi_A$ and $\phi_B$ denote the survival probabilities of uninfected and infected individuals, respectively. The transition probability $\psi_{AB}$ represents the likelihood of moving from the uninfected to the infected state (i.e., infection probability, also denoted as $p_{\text{inf}}$ in the individual-based model), while $\psi_{BA}$ represents the probability of transitioning from the infected to the uninfected state (i.e., recovery probability, also denoted as $p_{\text{recov}}$ in the individual-based model).

The first and fourth dimensions of $\Omega$ denote the states of departure and arrival, respectively, while the second dimension corresponds to the individual ($i$), and the third dimension to time ($t$).

## Movement process

The second state process models movement in Cartesian coordinates, specifically in the x- and y-directions. The location $G_{i,t}$ of individual $i$ at time $t + 1$ is modelled as a random walk that follows a normal distribution centered on its previous location:

$$G_{i,t+1} \sim \mathcal{N}(G_{i,t}, \sigma_G^2) \tag{3}$$

where $\sigma_G^2$ represents the movement variance in both the x- and y-directions. That is, $\sigma_G^2$ is a vector containing two values, one for the x-direction and one for the y-direction. Note that in the JAGS model implementation, the normal distribution is parameterised using the precision parameter $\tau$, defined as $1/\sigma_G^2$.

We also explored a $t$-distribution to model movement, as described in (Schaub and Royle 2014), but this did not improve the fit based on the Deviance Information Criterion (DIC) of the Bayesian model.

## Observation process

The final step of the Bayesian spatial multistate capture-recapture model is the observation process. Observation is conditional on survival and presence in the study area $A$. To determine if an individual is inside $A$, and in constrast to the non-spatial multistate capture-recapture model, this model considers a presence indicator variable $r_{i,t}$:

$$r_{i,t} = \begin{cases} 1, & \text{if } G_{i,t} \in A \\ 0, & \text{otherwise} \end{cases} \tag{4}$$

The observation process is modeled as:

$$y_{i,t} \mid z_{i,t}, r_{i,t} \sim \text{Categorical}\left(\mathit{\Theta}_{z_{i,t},i,t,1\ldots O} \cdot r_{i,t}\right) \tag{5}$$

where $y$ represents the observed multistate capture-recapture data. Similar to the first state process, $y$ is modeled using a categorical distribution with probabilities defined by the four-dimensional observation matrix $\boldsymbol{\mathit{\Theta}}$ (denoted as `po` in the JAGS model implementation). This matrix specifies the probabilities of observing state $O(t)$ given the latent state $S(t)$.

$$\begin{aligned} \mathit{\Theta}[1,i,t,1] &= pA[i,t] \\ \mathit{\Theta}[1,i,t,2] &= 0 \\ \mathit{\Theta}[1,i,t,3] &= 1 - pA[i,t] \\ \mathit{\Theta}[2,i,t,1] &= 0 \\ \mathit{\Theta}[2,i,t,2] &= pB[i,t] \\ \mathit{\Theta}[2,i,t,3] &= 1 - pB[i,t] \\ \mathit{\Theta}[3,i,t,1] &= 0 \\ \mathit{\Theta}[3,i,t,2] &= 0 \\ \mathit{\Theta}[3,i,t,3] &= 1 \end{aligned} \tag{6}$$

where $p_A$ and $p_B$ represent the recapture probabilities of uninfected and infected individuals, respectively. The first and fourth dimensions of $\mathit{\Theta}$ denote the true and observed states of an individual, respectively, while the second dimension corresponds to the individual ($i$), and the third dimension to time ($t$).

Note that for each individual ($i$), the observed state $O(t)$ is determined solely by the recapture probability associated with the individual's true (or latent) state during time $t$. That is, similar to the state process, the model assumes no error in state classification.

## Imputation submodel for missing covariate values

In one variation of our model, we evaluated whether the shortest distance to a Bd-positive individual for each focal individual $i$ at time $t$, $y_{\text{dist},i,t}$, influenced their infection probability $\psi_{AB,i,t}$ as:

$$\text{logit}(\psi_{AB}[i,t]) = \alpha + \beta \cdot y_{\text{dist},i,t} \tag{7}$$

where:

- $\alpha$ is the intercept term.
- $\beta$ represents the effect of distance on infection probability.
- $y_{\text{dist},i,t}$ is either an observed or imputed shortest distance.

However, as $y_{\text{dist}}$ is a time-varying covariate, for an occasion $t$ where individual $i$ was not captured, $y_{\text{dist},i,t}$ contains a missing value. This prevents parameter estimation because the likelihood of the model requires that from $f_i$ onward, all subsequent values of any time-varying covariate should be observed (i.e., provided to the model as data). Two general approaches for dealing with missing covariate values in this context exist: data imputation or the use of a conditional likelihood where only the observed covariate values are used (Bonner et al. 2010, Langrock and King 2013, Gimenez et al. 2018). Here, we followed the first approach.

Since movement distances were right-skewed, real distances were log-transformed as:

$$y_{\text{dist},i,t} = \log(\text{real dist}_{i,t} + 1) \tag{8}$$

where real $\text{dist}_{i,t}$ represents the raw distance data. If this transformed distance is observed, it is treated as given; otherwise, it is estimated using Bayesian imputation:

$$y_{\text{dist},i,t} \sim \mathcal{N}(\mu_t, \tau_t) \tag{9}$$

with hyperparameters defined hierarchically to incorporate temporal variability:

$$\mu_t \sim \mathcal{N}(\mu_{\text{global}}, \tau_{\text{global}}) \tag{10}$$

$$\tau_t = \frac{1}{\sigma_t^2}, \quad \sigma_t \sim \text{Uniform}(0.100, 1.428) \tag{11}$$

The global mean distance $\mu_{\text{global}}$ and global precision $\tau_{\text{global}}$ are modelled as:

$$\mu_{\text{global}} \sim \text{Uniform}(1.471, 3.901), \tag{12}$$

$$\tau_{\text{global}} = \frac{1}{\sigma_{\text{global}}^2}, \quad \sigma_{\text{global}} \sim \text{Uniform}(0.308, 0.550) \tag{13}$$

The informative priors used in equations (11), (12), and (13) were derived from log-transformed observed distance data specific to each geographical area (the values presented correspond to RFC). Specifically:

- In (11), we calculated $\sigma_t$ in the observed data and then used the minimum and maximum value as the lower and upper limit of the uniform prior distribution, respectively.
- In (12), lower and upper limit of the uniform prior distribution were determined as the observed $\mu_{\text{global}}$ minus and plus one standard deviation, respectively.
- In (13), we first calculated the observed $\mu_t$ and its standard deviation using bootstrapping (1,000 samples), and used the minimum and maximum bootstrapped standard deviations as the lower and upper limit of the uniform prior, respectively.

This hierarchical approach allows distance values to be estimated in a way that reflects overall patterns in the observed distance data for each occasion $t$ while preserving uncertainty in missing data points. This formulation also ensures that individuals with missing covariate values still contribute to the estimation of infection probability while accounting for uncertainty in their locations relative to Bd-positive individuals.

## Other predictors

Age class was used as an individual-level time-varying covariate in some of the model variations. For this variable, on occasions when individuals were not captured, their age class was assumed to be the same as the last age class recorded for that individual. Adding a dummy level to this factor for the occasions when individuals were not captured produced similar estimates for both the juvenile and adult age class, but these models did not reach chain convergence easily.

## Overall mortality probability and time to death quantile

In the main manuscript we present estimates of the complement of the overall (eventual) probability of recovery from *Bd* infection in *R. darwinii* from RFC. This was calculated as follows.

Once an individual becomes infected, its eventual recovery depends on surviving and recovering in any of the subsequent months. Let:

$$\phi_B: \text{ the monthly survival probability for infected individuals,}$$

$$\psi_{BA}: \text{ the monthly recovery probability.}$$

The process is as follows:

- **Month 1:** The probability of surviving and recovering is

$$\phi_B \times \psi_{BA}.$$

- **Month 2:** If the individual survives Month 1 without recovering, the probability of remaining infected is

$$\phi_B(1 - \psi_{BA}).$$

  Then, the probability of surviving and recovering in Month 2 is

$$\phi_B(1 - \psi_{BA}) \times \phi_B\psi_{BA}.$$

- **Month 3:** Similarly, the probability of recovering in Month 3 is

$$[\phi_B(1 - \psi_{BA})]^2 \times \phi_B\psi_{BA}.$$

In general, the probability of recovering in Month $n$ is:

$$[\phi_B(1 - \psi_{BA})]^{n-1} \times \phi_B\psi_{BA}.$$

Thus, the overall (eventual) probability of recovery is the sum of an infinite geometric series:

$$P_{\text{recover}} = \sum_{n=1}^{\infty} [\phi_B(1 - \psi_{BA})]^{n-1} \cdot \phi_B\psi_{BA} = \frac{\phi_B\psi_{BA}}{1 - \phi_B(1 - \psi_{BA})}.$$

The probability of eventually dying from infection (i.e., before recovering) is the complement of this:

$$P_{\text{death}} = 1 - P_{\text{recover}} = \frac{1 - \phi_B}{1 - \phi_B(1 - \psi_{BA})}.$$

### Time to death quantile

We also estimated the time point by which a given proportion of those that will eventually die (i.e., not recover) are expected to have died. Let $D_n$ denote the cumulative probability of dying while infected by month $n$, conditional on eventual death. Then:

$$D_n = \frac{\sum_{t=1}^{n} [\phi_B(1 - \psi_{BA})]^{t-1} (1 - \phi_B)}{P_{\text{death}}}.$$

Here, the numerator gives the cumulative probability of dying by month $n$, and the denominator rescales this to express it as a fraction of total expected deaths.

We report the smallest value of $n$ for which $D_n \geq 0.95$, that is, the time by which 95% of the individuals that will die from infection are expected to have done so.

# Supplementary Information: Prevalence and Spatial Clustering of Bd Infection

## Introduction

To assess whether there is spatial clustering in *Batrachochytrium dendrobatidis* (Bd) infection risk among *Rhinoderma darwinii* metapopulations, we developed a Bayesian model that estimates the difference between the observed number of Bd infections at each of six local populations within two study areas (RFC and HUI) and the expected number of infections under a scenario of spatially homogeneous infection risk. The expected number of Bd infections at each local population is derived from the estimated area-level prevalence of Bd infection and the number of individual hosts observed at each local population within a given area. The model is implemented in the BUGS language using JAGS (Plummer 2003).

## Model description

### Prevalence estimation

The model utilises month-specific Bd infection data collected across six monitored plots within each study area. The observed number of infected individuals in area $A$ at time $t$, denoted as $C_{A,t}$, is modelled as:

$$C_{A,t} \sim \text{Binomial}(N_{A,t}, p_{A,t}), \tag{1}$$

where $N_{A,t}$ represents the total number of individual hosts observed in area $A$ at time $t$, irrespective of their infection status, and $p_{A,t}$ is an unobserved latent variable describing the probability of an individual being Bd-infected in area $A$ at time $t$. When multiplied by 100, $p_{A,t}$ corresponds to the area-level prevalence of Bd infection.

In the JAGS model implementation, we assume a uniform prior for $p_{A,t}$:

$$p_{A,t} \sim \text{Uniform}(0, 0.3). \tag{2}$$

## Expected infections at each plot

For each plot $s$ within area $A$, the expected number of Bd-infected individuals at time $t$ is modelled as:

$$\text{Expected Infections}_{s,A,t} \sim \text{Binomial}(n_{\text{frog},s,A,t}, p_{A,t}), \quad (3)$$

where $n_{\text{frog},s,A,t}$ represents the number of *R. darwinii* frogs captured at plot $s$ in area $A$ at month $t$.

The difference between the observed and expected number of Bd-infected individuals is then calculated as:

$$\Delta\text{Infections}_{s,A,t} = n_{\text{bd},s,A,t} - \text{Expected Infections}_{s,A,t}, \quad (4)$$

where $n_{\text{bd},s,A,t}$ is the observed number of Bd-infected individuals at plot $s$ in area $A$ at month $t$.

Therefore, $\Delta\text{Infections}_{s,A,t}$ quantifies whether certain plots exhibit a higher-than-expected number of Bd-infected individuals for area $A$ at month $t$, which would indicate spatial clustering of Bd infection risk.

Results from these analyses are presented in Supplementary Fig. 2.

# Supplementary Information: Environmental Variables

In Field Study 1, we deployed an air temperature and humidity data logger (RHT10, Extech Instruments Corporation, Waltham, MA, USA) at the centre of each plot. The data loggers were placed 15 cm above ground level and covered by a plastic sheet to avoid direct sunlight. The devices were set to measure air temperature (°C) and air relative humidity (%) every 30 min for approximately 38 days per area (RFC:34, HUI:41) distributed across eight months, totalling 21,582 individual records for each variable (RFC: 9,714; HUI: 11,868), collected across six plots per area (Supplementary Fig. 13). Data within each area were collected during the same period, although there was generally about one week difference in the collection periods between RFC and HUI. During each month, data was collected during approximately 5 days per area. Using the raw data, we calculated for each month the (a) mean air temperature and (b) mean air relative humidity. We also calculated the fluctuation in these variables (defined as the difference between the 99% and 1% percentiles of each variable during the measurement period each month), specifically (c) air temperature fluctuation and (d) air relative humidity fluctuation. To evaluate differences in the four microclimatic variables among plots, we used a Bayesian linear model implemented in JAGS (Plummer 2003) through the R package `jagsUI` (Kellner 2015). In this model, the monthly value of each variable was the dependent variable, while plot was used as a predictor.

We also measured the diameter at breast height (DBH) of all trees within each plot. From these data, we calculated tree basal area ($m^2$ $ha^{-1}$) as the sum of the cross-sectional areas of all trees with a DBH greater than 5 cm. As we did not expect to detect considerable changes in tree size during the study period, this metric was measured only once per plot at the beginning of the study.

In Supplementary Table 4 we show a summary of the measured environmental variables for each plot in RFC and HUI. In Supplementary Figure 13 we show the raw microclimatic data collected each month, and in Supplementary Figures 14-15 a detailed summary of the temporal and spatial variation in these variables. In Supplementary Table 3 we also present a pairwise comparison of environmental variables between plots. Overall, our results show there was little variation in environmental variables among plots both at RFC and HUI.

# Supplementary Information: Spatial Point Pattern Analysis

## Introduction

We conducted an analysis to characterise the spatial distribution of *R. darwinii* and sympatric amphibians in our study areas, providing essential input for an individual-based model (IBM) designed to describe the dynamics of *Batrachochytrium dendrobatidis* infection in these amphibian populations. As we found spatial clustering in the distribution of *R. darwinii* during a preliminary analysis, we decided to fit a Cox process model, which is commonly used to model clustered point patterns. A Cox process (also known as a doubly stochastic Poisson process or modulated Poisson process) is essentially a Poisson process with a random intensity function (Baddeley et al. 2016). This model assumes an underlying, spatially varying intensity function that is random because it depends on unobservable external factors (e.g., spatially varying infection risks influencing host patterns or microclimatic variations) and measured covariates (Baddeley et al. 2016). Spatial variation in the random intensity function creates clustering where there are "hot spots" in the intensity. Such models are typically used when the point pattern is suspected to be influenced by unobserved covariates (Baddeley et al. 2016). Specifically, we fitted a log-Gaussian Cox process (LGCP) model with an exponential covariance function (also known as a correlation matrix). We employed the quasi-likelihood method to improve intensity estimation in the LGCP model (Baddeley et al. 2016).

## Log-Gaussian Cox process (LGCP) model

The LGCP is essentially a Poisson model with a random Gaussian intensity field. This means the intensity changes spatially, following a multivariate Gaussian probability distribution governed by two key parameters: variance and scale (Baddeley et al. 2016). The variance determines the magnitude of fluctuations in the log-intensity field, where higher variance leads to more extreme high and low-intensity regions. Conversely, smaller variance produces a smoother intensity field with less variation. The scale parameter governs the range over which the log-intensity values are spatially correlated (also referred to as the correlation parameter; Baddeley et al. 2016). A larger scale indicates that the intensity field changes gradually over space, resulting in broader, smoother clusters. A smaller scale implies rapid changes in intensity, creating finer, more localised clusters. An LGCP with an extremely small scale parameter approaches a Poisson process (Baddeley

et al. 2016). Put simply, variance controls the "height" of peaks and "depth" of troughs in intensity, while the scale determines the spatial distance between these peaks and troughs. In our analysis, variance and scale parameters exhibited a negative correlation, which is a common feature observed in practice when fitting LGCP models (Supplementary Fig. 16).

# Fitting the LGCP model to empirical data

We applied the LGCP model to point pattern data representing the spatial locations of *R. darwinii* in three geographical areas: RFC, HUI, and TAN. For RFC and HUI, we used novel data as described in the main manuscript, and for TAN, we used unpublished data from Valenzuela-Sánchez et al. (Valenzuela-Sánchez et al. 2014). TAN data were collected at TAN2, the same local population described in the main manuscript. At RFC and HUI, frogs were surveyed in six plots, but to increase sample size and provide better model parameter estimates, we merged data within each area. Fitting LGCP models to plot-level data showed inadequate fit, as demonstrated by exploration of L and G functions with global envelopes (see below). We excluded two months (November 2018 and March 2022) from HUI due to outlier scale parameter values that were five to six orders of magnitude larger than others, likely resulting from estimation issues.

We fitted separate LGCP models for data collected in individual months. Each month included three secondary capture occasions, but for this analysis, we retained only the spatial location of individuals during their first capture for a given month. To avoid point overlap, which can disrupt point pattern modelling, we applied random jitter to the points within a 0.1 m radius using the `rjitter` function in the `spatstat` package (version 3.0-3; Baddeley et al. 2016). LGCP models were fitted using the `kppm` function in `spatstat`.

# Null hypothesis testing

For null hypothesis testing of the LGCP model, we used the L and G functions with global envelopes. The L-function, a variance-stabilising transformation of Ripley's K-function, is recommended for global envelope tests (Baddeley et al. 2016). For the G-function, we used Fisher's arcsine transformation, which approximates variance stabilisation and is also recommended for global envelope tests (Baddeley et al. 2016). The null hypothesis (a fitted LGCP model) is rejected if the observed summary function falls outside the global envelopes (Baddeley et al. 2016). Using global envelopes, the significance level is determined by $k/(m+1)$, where $nrank = 10$ and $nsim = 199$ yield a significance level of 0.05. LGCP models fitted to area-level data showed adequate fit across all months in RFC, HUI, and TAN (Supplementary Figs. 17–22).

# LGCP model results

Among-area comparisons revealed no differences in LGCP model parameters (scale and variance), as shown by Bayesian linear model results and visual inspection (Supplementary Figs. 23 and 24). This suggests that the spatial distribution of *R. darwinii* individuals, or the statistical description of the resulting point pattern, is similar across populations of *R. darwinii* in different geographical areas, although intensity varies greatly (Supplementary Fig. 23).

At RFC, we also compared an LGCP model fitted to *R. darwinii*-only data with a model fitted to data that included *Eupsophus* spp (Supplementary Fig. 24). Both models showed no significant differences, as evidenced by Bayesian linear model results and visual inspection (Supplementary Figs. 25 and 26). Thus, the spatial distribution of *Eupsophus* does not need to be modelled differently from that of *R. darwinii* in the IBM.

Finally, as no significant among-area variation was found in the spatial distribution of *R. darwinii*, we calculated the mean LGCP model parameters using *R. darwinii*-only data for the IBM: variance = 3.54 and scale = 1.51.

# Supplementary Information: Individual-based Model

## Introduction

We implemented a discrete individual-based model (IBM) to simulate the spatial, demographic, and epidemiological dynamics of a *Rhinoderma darwinii* population and syntopic amphibians over a 12-month period in monthly time steps. We use the term “syntopic amphibians” to represent any anuran different from *R. darwinii* that might overlap spatially with individuals of this species. Due to a lack of data from most syntopic anuran species in our system, when parameterising the IBM for syntopic species we used only estimates from *Eupsophus* spp. collected at RFC. *Eupsophus* spp. are the most common syntopic amphibians in our study system, but at least other five species (*Batrachyla leptopus*, *B. antartandica*, *B. taeniata*, *Pleurodema thaul*, and *Hylorina sylvatica*) can also be found in the environments used by *R. darwinii*.

The model accounts for the spatial distribution of individuals, individual movement, infection by *Batrachochytrium dendrobatidis* (Bd), recovery, and survival. As we were interested in short-term, within-year dynamics, the model does not consider the birth of individuals during the simulation period.

## Model description: IBM function

### Initial population and spatial setup

The first step of the IBM function is to randomly generate the starting spatial location (see subsection “Movement dynamics” below) of *R. darwinii* or syntopic individuals. For this, we use a Log-Gaussian Cox process (LGCP) to simulate spatial point patterns representative of the spatial distribution of frogs in a local population. The LGCP point pattern is generated using the `spatstat` R package (Baddeley et al., 2016). A stationary LGCP model is defined within a square observation window, $W = [0, 20] \times [0, 20]$ (in metres), with parameters controlling the intensity (density, in an ecological context) and the Gaussian field. For the latter, we used average model parameters estimated by fitting the LGCP model to empirical data collected at RFC, HUI, and TAN (variance = 3.54 and scale = 1.51; for further details, see Supplementary Material: Spatial Point Pattern Analysis).

The log-intensity of the LGCP was defined as:

$$\mu = \log(\lambda) - \frac{\text{variance}}{2}. \tag{1}$$

where variance $= 3.54$ (which governs fluctuations in the Gaussian random field), and $\lambda$ is the expected number of individuals per unit area. Each point pattern is generated on a $40 \times 40$ grid to ensure fine spatial resolution in the intensity field.

## Population Structure

Each *R. darwinii* individual in the population is assigned an age class (juvenile or adult) and an infection status (Bd-positive or Bd-negative). Thus, at any time step $t$ each individual $i$ is classified into one of five states:

$$S_{i,t} = \begin{cases} 1, & \text{juvenile Bd-negative (uninfected)}, \\ 2, & \text{juvenile Bd-positive (infected)}, \\ 3, & \text{adult Bd-negative (uninfected)}, \\ 4, & \text{adult Bd-positive (infected)}, \\ 0, & \text{dead}. \end{cases} \tag{2}$$

For the initial state ($t = 1$), the assignment is based primarily on age. Let $p_{\text{juv}}$ denote the proportion of juveniles in the population. In the default setup, individuals are assigned to the four living states with the following probabilities:

$$\begin{aligned} P\Big(S_{i,1} = 1\Big) &= p_{\text{juv}}, & \text{(juvenile uninfected)}, \\ P\Big(S_{i,1} = 2\Big) &= 0, & \text{(juvenile infected)}, \\ P\Big(S_{i,1} = 3\Big) &= 1 - p_{\text{juv}}, & \text{(adult uninfected)}, \\ P\Big(S_{i,1} = 4\Big) &= 0, & \text{(adult infected)}. \end{aligned} \tag{3}$$

In other words, by default, no individual is initially infected.

To allow Bd introduction into the population, one randomly selected *R. darwinii* individual is re-assigned an infected state. The infection is allocated such that the individual becomes either a juvenile infected (state 2) or an adult infected (state 4) with equal probability.

Due to the lack of age-specific demographic and infection estimates for syntopic amphibians in our system, their state is categorised solely by infection status. In the main IBM, syntopic amphibians are assumed to acquire infection only secondarily; however, we also explored an alternative scenario in which Bd introduction occurs in a syntopic individual rather than a *R. darwinii* individual.

## Movement dynamics

Each individual is also assigned an initial spatial location, $(x_{i,1}, y_{i,1})$, based on the simulated point patterns (see subsection "Initial population and spatial setup" above). The initial spatial location also serves as their fixed centre of activity, $(x_{c_i}, y_{c_i})$. For $t \in \{2, 4, 6, 8, 10, 12\}$, the location for each individual at time $t$ is calculated based on random movement drawn from a normal distribution:

$$x_{i,t} \sim \mathcal{N}(x_{c_i}, \sigma), \quad y_{i,t} \sim \mathcal{N}(y_{c_i}, \sigma), \tag{4}$$

where $\sigma$ represents the standard deviation of movement in the $x$- and $y$-directions, with the model allowing different values for juveniles ($\sigma_{\text{juv}}$) and adults ($\sigma_{\text{adu}}$). In the simulations, we used $\sigma$ estimates obtained by fitting the spatial multistate model to empirical spatial capture-recapture data collected at HUI and RFC (see main manuscript) and averaged these values for each age class. This averaging resulted in similar values for $\sigma_{\text{juv}}$ and $\sigma_{\text{adu}}$, both of which were close to 5.000. In Supplementary Fig. 27, we show how this $\sigma$ value translates into linear monthly displacement distances.

To preserve the spatial clustering pattern observed in the empirical data while mimicking high levels of site fidelity in *R. darwinii* (Valenzuela-Sánchez et al., 2019), individuals are relocated to their fixed centres of activity during the months $t \in \{3, 5, 7, 9, 11\}$.

The IBM function uses the same movement dynamics and parameter values for *R. darwinii* and syntopic amphibians, but it can be easily extended to include interspecific variation in movement behaviour if required.

## State transition dynamics

At each monthly time step, $t$ to $t + 1$, state transitions in *R. darwinii* are governed by the matrix $T$:

$$T = \begin{bmatrix} \phi_{\text{rd,juv,neg}}\,(1 - p_{\text{inf}_{i,t}}) & \phi_{\text{rd,juv,neg}}\,p_{\text{inf}_{i,t}} & 0 & 0 & 1 - \phi_{\text{rd,juv,neg}} \\ \phi_{\text{rd,juv,pos}}\,p_{\text{recov}} & \phi_{\text{rd,juv,pos}}\,(1 - p_{\text{recov}}) & 0 & 0 & 1 - \phi_{\text{rd,juv,pos}} \\ 0 & 0 & \phi_{\text{rd,adu,neg}}\,(1 - p_{\text{inf}_{i,t}}) & \phi_{\text{rd,adu,neg}}\,p_{\text{inf}_{i,t}} & 1 - \phi_{\text{rd,adu,neg}} \\ 0 & 0 & \phi_{\text{rd,adu,pos}}\,p_{\text{recov}} & \phi_{\text{rd,adu,pos}}\,(1 - p_{\text{recov}}) & 1 - \phi_{\text{rd,adu,pos}} \\ 0 & 0 & 0 & 0 & 1 \end{bmatrix} \tag{5}$$

where $\phi_{\text{rd,juv,neg}}$ and $\phi_{\text{rd,adu,neg}}$ denote the monthly survival probabilities of Bd-negative *R. darwinii* juveniles and adults, respectively, and $\phi_{\text{rd,juv,pos}}$ and $\phi_{\text{rd,adu,pos}}$ denote those for Bd-positive *R. darwinii* juveniles and adults. The term $p_{\text{inf}_{i,t}}$ is the infection probability experienced by individual $i$ at time $t$, and $p_{\text{recov}}$ represents a fixed probability of recovery from infection. In $T$, the rows correspond to the states at time $t$ and the columns to the states at time $t + 1$.

To calculate $p_{\text{inf}_{i,t}}$, the model first calculates the minimum distance of individual $i$ to any Bd-positive individual regardless of their species identity at time $t$, denoted by $d_{i,t}$. This distance is then used to calculate the infection probability as:

$$p_{\text{inf}_{i,t}} = \frac{\exp\Big(\alpha_{p_{\text{inf,rd}}} + \beta_{p_{\text{inf,rd}}} \log\big(d_{i,t} + 1\big)\Big)}{1 + \exp\Big(\alpha_{p_{\text{inf,rd}}} + \beta_{p_{\text{inf,rd}}} \log\big(d_{i,t} + 1\big)\Big)}. \tag{6}$$

Here, $\alpha_{p_{\text{inf,rd}}}$ and $\beta_{p_{\text{inf,rd}}}$ are the intercept and slope of a logistic regression that models the probability of infection as a function of $\log(d_{i,t} + 1)$ in *R. darwinii* (see Supplementary Material: Bayesian spatial multistate capture-recapture model). In syntopic species, p_{inf_{i,t}} is calculated in the same way but using $\alpha_{p_{\text{inf,syntopic}}}$ and $\beta_{p_{\text{inf,syntopic}}}$.

If no Bd infected individuals are present at time $t$, the infection probability is set to a baseline value, $p_{\text{base}}$. When $p_{\text{base}} = 0$, the pathogen is extirpated from the population, preventing re-invasion during a given simulation.

As in syntopic amphibians we only used the infection status categories, the state transitions were calculated using a simplified matrix, using the same probabilities of infection and recovery from Bd infection as in *R.darwinii* but syntopic-specific survival probabilities.

## Population depression in *R. darwinii*

To quantify the population-level impact of Bd infection in *R. darwinii*, we use a metric termed population depression, $\delta$. This metric is defined as the proportional reduction in the observed population size due to Bd infection relative to a counterfactual scenario where Bd is absent.

Let $N^{+}_{t=12}$ denote the observed population size at the end of the simulation ($t = 12$) when Bd is present (i.e., including all individuals that have not died), and let $N^{-}_{t=12}$ denote the expected population size at $t = 12$ under a no-pathogen scenario, in which Bd transmission is prevented and individuals transition solely based on their age-specific survival probabilities in the absence of Bd infection (i.e., $\phi_{\text{rd,juv,neg}}$ and $\phi_{\text{rd,adu,neg}}$). Then, population depression is given by:

$$\delta = 1 - \frac{N^{+}_{t=12}}{N^{-}_{t=12}}. \tag{7}$$

A value of $\delta = 0$ indicates no population depression (i.e., the population size when Bd is present is equal to that when Bd is absent), whereas a value close to 1 indicates high Bd-induced mortality. In the extreme case, $\delta = 1$ if the population with Bd becomes extinct.

## Epidemiological metrics

The IBM function computes several metrics that capture the dynamics of Bd infection. The epidemic ratio $R_t$ is calculated as the ratio of the number of new infections occurring in month $t + 1$ to the total number of infected individuals present in month $t$ (Ferrari et al. 2005):

$$R_t = \frac{N_{\text{new, infected, } t+1}}{N_{\text{infected, } t}}, \tag{8}$$

The basic reproduction number $R_0$ (i.e., average number of infections arising during the infectious period of a single individual entering a population of entirely susceptible hosts; Foufopoulos et al. 2022) can be estimated as the intercept of a linear regression of $R_t$ on the cumulative number of infectious individuals at time $t$ (Ferrari et al. 2005, Foufopoulos et al. 2022):

$$R_t \ = R_0 - \beta \sum_{i=1}^{t} infected \tag{9}$$

To stabilise the variance on the residuals, the linear regression is performed as a weighted linear regression, with weights proportional to the number of infected individuals at time $t$ (Ferrari et al. 2005). To correct for bias in the estimation of $R_0$ due to the discretisation of a continuous epidemic process, $R_0$ is corrected as (Ferrari et al. 2005):

$$R_{0,\text{corrected}} \approx R_0 2(1 - e^{-1}) - e^{-1} \tag{10}$$

For each month $t$ (with $t \ \geq \ 2$), the model also calculates the average monthly infection probability for *R. darwinii* as:

$$PrInf_{\text{rd},t} = \frac{N_{\text{new, infected, rd}, t+1}}{N_{\text{susceptible, rd}, t}}, \tag{11}$$

where $N_{\text{new, infected, rd}, t+1}$ denotes the number of *R. darwinii* individuals that were susceptible (uninfected) at month $t$ and became infected during month $t \ + \ 1$, and $N_{\text{susceptible, rd}, t}$ represents the total number of susceptible (uninfected) *R. darwinii* individuals at month $t$. This formulation resembles how a constant (population-averaged) infection probability is estimated in a multistate capture-recapture model.

In addition, the model records a binary indicator variable, $Bd_{\text{fadeout}}$, which is set to 1 if no Bd-infected individuals are present at any time step (indicating permanent pathogen extirpation when $p_{\text{base}} = 0$) and 0 otherwise.

In Supplementary Table 5 we detail the epidemiological and demographic metrics returned by the IBM function.

# Global sensitivity analysis

We conducted a global sensitivity analysis (GSA) of our IBM, following the methods described in Harper et al. (2011) and Wilber et al. (2017). Sensitivity analyses are a critical step in simulation studies as they allow for the evaluation of the relative importance of input parameters in influencing model outcomes. In our case, GSA helps assess the impact of input parameters on the epidemiological and demographic metrics returned by our IBM function. GSA achieves this by constructing a set of scenarios, or unique combinations of parameter values—typically between 1,000 to 2,000 are sufficient when evaluating 10–20 input parameters (Harper et al. 2011). In each scenario, input parameters are perturbed (i.e., randomly sampled from predetermined options or probability distributions), and the model is fitted to the resulting dataset. Regression-based or machine learning methods are then used to quantitatively assess the relative importance of each input variable on a given model output (response variable).

We perturbed 19 input parameters (Supplementary Fig. 28) either by drawing a random value from a normal distribution and adding it to the logit-transformed initial parameter value (for input parameters in the probability scale) or by drawing a random value from a log-normal distribution and multiplying it by the initial parameter value (for input parameters in the continuous scale) (Supplementary Table 6). We randomly perturbed the parameters to construct 2,000 scenarios of parameter combinations. In each scenario, we ran 500 simulations of the IBM function and saved the median value of the model outcomes evaluated in the GSA (Supplementary Table 7).

We fitted GLM-based and random forest models to the resulting dataset to assess the importance of each input parameter in explaining the epidemiological and demographic output variables (Supplementary Table 8). In these analyses, all input parameters were scaled (z-transformed) to ensure comparability and improve the interpretability of their relative importance in model outcomes. We evaluated the performance of the GLM-based models using standard visual diagnostics, including residuals vs. fitted values, Q-Q plots, Scale-Location plots, and Cook's distance. Additionally, we assessed model performance in the logistic regression using ROC curve analysis.

Variable importance rankings were derived from standardised regression coefficients in GLM-based models and Mean Decrease in Accuracy (MDA) from random forest models. For the latter, the MDA scores returned by the `randomForest` R package (Liaw and Wiener 2002) were scaled using proportional normalisation, ensuring that the resulting values typically range between 0 and 1 (although negative values can occur when an input variable has minimal relevance and may introduce noise into the model). In general, the relative importance of the parameters was consistent between the GLM-based and random forest results (Supplementary Fig. 5).

## Simulation experiments

The sensitivity analysis showed that the variables $\alpha_{p_{\text{inf,rd}}}$, $\beta_{p_{\text{inf,rd}}}$, $\lambda_{\text{rd}}$, and $\lambda_{\text{syntopic}}$ consistently had the largest relative importance in the outcomes of the IBM (Supplementary Fig. 5). Therefore, we designed two simulation experiments to further explore the impact of these variables on $\delta$, $R_0$, $Bd_{\text{fadeout}}$, and $n_{\text{epidemic,rd}}$.

In the first simulation experiment, we explored $\alpha_{p_{\text{inf,rd}}}$ and $\beta_{p_{\text{inf,rd}}}$. We followed a full factorial design composed of all combinations of $\alpha_{p_{\text{inf,rd}}}$ from $-1.787$ to 3.311 (100 levels) and $\beta_{p_{\text{inf,rd}}}$ from $-2.867$ to $-0.562$ (100 levels), which are close to the most extreme 95% Bayesian credible intervals for these parameters as estimated at HUI and RFC in this study, and three levels of $\lambda_{\text{rd}}$ (0.01, 0.13, 0.25) and $\lambda_{\text{syntopic}}$ (0, 0.12, 0.24), which correspond to low/none, medium, and high densities for *R. darwinii* and syntopic species, respectively (see below). All other parameters used only the default values as detailed in Supplementary Table 6. This resulted in 90,000 parameter combinations. For each combination, 1,000 independent replicates of the IBM were simulated, and the simulation outputs were aggregated by calculating the median value across replicates for each output metric, thereby providing central estimates of the stochastic model's behavior.

In the second simulation experiment, we explored $\lambda_{\text{rd}}$ and $\lambda_{\text{syntopic}}$. We evaluated all combinations of $\lambda_{\text{rd}}$ from 0.01 to 0.25 frogs m$^{-2}$ (97 levels) and $\lambda_{\text{syntopic}}$ from 0 to 0.24 frogs m$^{-2}$ (97 levels). We used two unique combinations of $\alpha_{p_{\text{inf,rd}}}$ and $\beta_{p_{\text{inf,rd}}}$: the mean parameter estimates from RFC ($-0.976$ and $-0.936$, respectively) and from HUI (0.779 and $-1.767$, respectively). All other parameters used only the default values as detailed in Supplementary Table 6. This resulted in 18,818 parameter combinations. For each combination, 1,000 independent replicates of the IBM were simulated, and results were summarised using the median as described above.

The values of $\lambda_{\text{rd}}$ explored ensure variation in density consistent with empirical observations of free-living *R. darwinii* populations (Valenzuela-Sánchez et al., 2017, 2022; Supplementary Table 8). In syntopic amphibians, the estimated maximum density in *Eupsophus* spp. in RFC was $\lambda = 0.08$ frogs m$^{-2}$, three times lower than the maximum $\lambda_{\text{rd}}$ value explored in the IBM. We made this decision because our estimates of *Eupsophus* spp. density are most likely an underestimation of the density of syntopic anurans. First, our survey methodology is a diurnal visual encounter survey in which we walk through the forest searching for amphibians on the surface or between vegetation and twigs. To minimise habitat perturbation, we avoid searching under natural cover objects, and most such objects cannot be moved due to their large size. This search-encounter methodology is well suited for *R. darwinii* because the species is active on the forest floor during the day, whereas most syntopic species are under natural cover or hiding in inaccessible places during daytime surveys (Rabanal and Nuñez 2008). For instance, *Batrachyla* spp. are seldom captured during diurnal visual encounter surveys but are easy to find during night surveys. Indeed, we did not capture this species at RFC and only captured one individual at HUI, despite males being heard calling frequently in both areas. If individuals are not available for capture, even capture-recapture methods will lead to an underestimation of true abundance. Second, our field experience indicates that the abundance of syntopic

species is much higher in areas such as Chiloé Island (where TAN1 and TAN2 populations are located) than in RFC, although a formal estimation of this has not been conducted. Therefore, we believe the range of $\lambda_{\mathrm{rd}}$ values explored is most likely well within the natural abundance achieved by these syntopic species.

The simulation workflow was implemented in R using parallel processing.

The main results from the simulations are presented in Supplementary Figures 29-41.

# Supplementary Information: Role of Syntopic Amphibians in Bd Transmission

A key unresolved question is how individuals of a fully terrestrial species such as *R. darwinii* come into contact with a fungus with an aquatic infective stage such as Bd. While occasional dispersal of Bd from aquatic environments into terrestrial habitats via rain (Kolby et al. 2015), fog (Prado et al. 2023), or avian vectors (Hanlon et al. 2017) could be possible, these events are likely infrequent. Instead, syntopic amphibian species that utilise both freshwater and terrestrial habitats throughout their life cycles might be important for Bd dispersal into *R. darwinii* populations and for the spread of this pathogen once present in a local population (Valenzuela-Sánchez et al. 2017). Indeed, a greater abundance of syntopic amphibians in RFC compared to HUI (see main manuscript, Methods section, Observational study: design) could partially account for the higher Bd infection probability in *R. darwinii* in this area.

## Exclusion experiment

To experimental investigate the role of syntopic species in Bd transmission within *R. darwinii* populations, we conducted an exclusion experiment using solid fences and removal of syntopic amphibians (Supplementary Figs. 42a and 43). From 2018 to 2020, we conducted this experiment where only *R. darwinii* individuals were maintained inside fenced areas and all captured syntopic amphibians were removed. We were interested in evaluating if this management action can lead to a reduction in Bd infection probability in *R. darwinii*. To prevent the entry (by walking or climbing) of any amphibian, a solid metal fence of 1.15 m tall (15 cm buried) was built surrounding a natural *R. darwinii* population. We set up one replicate of this experiment in RFC, and two in HUI (Supplementary Fig. 42a). The average size of the fenced areas was 1,731 m2. The spatially structured populations from the observational study described above were used as controls in this experiment. From November to March, each fenced area was surveyed once per month using the same methodology described for the observational study. During the study period, we collected individual-level demographic and Bd infection data on 1,294 captures of 329 *R. darwinii* frogs (RFC: 108, HUI: 221). Additionally, we removed 62 frogs from the fenced areas (RFC: 45, HUI: 17), comprising five syntopic species (Supplementary Table 9).

We estimated survival and infection probability using the non-spatial multistate model because in this experiment we did not record the spatial location of individuals. However,

as emigration was prevented by the fences, we assume the estimated apparent survival represents the true survival probability. Indeed, true survival of uninfected frogs in the observational study estimated using the spatial multistate model were very similar to the estimates of survival of uninfected frogs in the exclusionary fences estimated using the non-spatial model.

This intervention did not result in a measurable reduction in Bd infection probability in *R. darwinii* after 2–4 years (Supplementary Fig. 42a). However, these results should be interpreted with caution, as two of the replicates were conducted in HUI, an area where Bd infection probability was naturally low, limiting potential effect sizes. Additionally, in RFC the experiment had to be prematurely terminated due to a surge in violent attacks in the area (see Methods section, 'Observational study: design'). At the time of the interruption, the abundance of syntopic species had not yet been sufficiently reduced, leaving the intervention incomplete (Supplementary Table 9). These results are presented to demonstrate the feasibility of this potential mitigation action in a real-world context, along with the associated challenges, in the hope that this information will support other researchers and conservation managers.

# Social network analysis

We additionally conducted social network analysis using spatial data from *R. darwinii* and syntopic species (mostly *Eupsophus* spp. individuals from RFC) to better understand the role of level of spatial overlapping between these species and the potential for interspecific Bd transmission. Using spatial data from Field Study 1, we analysed daily displacement distances of frogs to define the distance cut-off for a dyad (i.e. a pair of individuals) to be considered active (i.e. the pair of frogs was considered as having contacted). This information was used in the social network analysis. The number of possible dyads in an area can be calculated as n$x$ (n-1)/2, where n is the total number of individuals. For individuals with two observed displacements per primary capture occasion, we only retained the mean of these displacements. The cut-off was defined as the 80% percentile of the daily displacement distance observed across all primary capture occasions (i.e., 1.552 m). To define which dyads were active, we calculated the linear distance between all possible combinations of the spatial locations of each member of each potential dyad within each primary capture occasion. For instance, in a potential dyad where both members were captured during all the three secondary capture occasions, there are nine measurable distances within a primary capture occasion. If at least one of the measurable distances between members of a potential dyad within a primary capture occasion was equal or shorter than the cut-off distance, that dyad was considered as active in that primary capture occasion. We made this decision because we assume that both direct and indirect parasite transmission are relevant for Bd spread in this system (e.g. Burns et al. 2021), and because Bd zoospores are likely to remain infective in wet substrate in the forest floor during the duration of a primary capture occasion (which was generally less than 48 h since the first until the last survey during a month). For example, indirect transmission of the chytrid fungus B. salamandrivorans has been experimentally

demonstrated to occur up to 48 h after the soil had been in contact with an infected amphibian (Stegen et al. 2017).

The social network analysis was performed using data from all amphibian species, as well as using data from *R. darwinii* only. We calculated network-level metrics (edge density and transitivity) and individual-level metrics (degree centrality) using the R package igraph as detailed in Silk et al. (Silk et al. 2017). Edge density is defined as the proportion of completed edges (i.e. contacts) in a network. In the context of parasite transmission, all else being equal, edge density is expected to be positively associated with the velocity of infection spread (Silk et al. 2017). Transitivity is a measure of clustering, i.e. the probability that the adjacent nodes (i.e. frogs) of a node are connected. A higher transitivity indicates the network is more clustered into modules, a situation that hinders infection spread (Silk et al. 2017). Degree centrality is the number of edges or contacts an individual has. Individuals with a higher degree centrality are generally expected to have an increased likelihood of acquiring infection from another individual in the population, and to spread infection to a larger number of individuals (Silk et al. 2017). We decided to conduct the analyses for each plot separately because within a primary capture occasion each plot was effectively an isolated entity.

Interspecific interactions, defined as instances of spatial proximity between *R. darwinii* and *Eupsophus* spp. individuals, were frequent in RFC, accounting for an average of 14.4% of all detected dyads (i.e., pairs of individual amphibians) in this area (Supplementary Fig. 42c), underscoring the potential for interspecific Bd transmission in our study system. The social network analysis conducted at the local population level in RFC revealed that the presence of syntopic amphibians did not increase the connectivity (measured using edge density) nor decreased clustering (measured using transitivity) of the system in comparison to a network constructed with *R. darwinii* individuals only. These results suggest syntopic amphibians did not play a more significant role than *R. darwinii* individuals in facilitating Bd spread within local populations (Supplementary Fig. 42d).

Syntopic amphibians may still contribute significantly to Bd transmission and persistence in this system if they exhibit higher Bd prevalence or greater infection tolerance, remaining infectious longer than *R. darwinii.* At RFC, *Eupsophus* spp. showed a similar Bd prevalence to *R. darwinii* across all months (average prevalence: *Eupsophus* spp. = 11.2%, *R. darwinii* = 9.5%; Supplementary Fig. 44). Bd infection probability was higher in *Eupsophus* spp. (Supplementary Fig. 45a), but with moderate statistical support for an interspecific difference (84% probability). Sparse CR data prevented confident estimation of survival rates in Bd-infected syntopic frogs, which could inform infection tolerance. However, lower Bd infection intensity (Supplementary Fig. 45b) and the absence of detectable Bd-driven declines, despite frequent infections, suggest greater Bd tolerance in syntopic amphibians such as *Eupsophus* spp. and *Batrachyla* spp. (Bacigalupe et al. 2017).

# Supplementary Information: Probability of Dispersal

## Introduction

We investigated inter-plot movement (hereafter referred to as "dispersal") in *Rhinoderma darwinii* using capture-recapture (CR) data. Due to the species' strong site fidelity, dispersal events between plots were infrequent. This sparsity prevented the use of multistate or multievent models, which are commonly applied to estimate dispersal probabilities from CR data (Cayuela et al. 2018). Instead, we analysed the complete capture history of individuals to estimate the probability of dispersing at least once during the study period. A Bayesian binomial model was used to estimate the mean annual dispersal probability at both the plot and area level.

This analysis was restricted to individuals that were captured in at least two different months. Only 4.2% of the *R. darwinii* individuals captured more than once in RFC dispersed at least once during the study period. In HUI, 11.5% of the individuals captured more than once dispersed at least once during the study period. A total of 36 dispersal events were observed (15 in RFC and 21 in HUI), involving 27 individuals (10 in RFC and 17 in HUI) (Supplementary Fig. 46 and Supplementary Table 10). More dispersal events were observed in HUI than in RFC, likely due to the longer monitoring period: data were collected at RFC from November 2018 to March 2020, whereas at HUI, monitoring continued until March 2022. However, at an annual scale, dispersal probability is very similar among areas (see below).

## Model description

### Bayesian binomial model for dispersal probability

A Bayesian binomial model was implemented in JAGS (Plummer 2003) to estimate the probability of dispersal at the plot level. The model assumes that an individual's dispersal event follows a Bernoulli distribution:

$$D_i \sim \text{Bernoulli}(pd_i), \tag{1}$$

where $D_i$ is a binary variable indicating whether individual $i$ dispersed at least once during the study period ($D_i = 1$ if dispersal occurred, $D_i = 0$ otherwise), and $pd_i$ is the probability of dispersal.

The model estimates separate dispersal probabilities for each of the six monitored plots within each area, denoted as $p_{\text{disp},s}$, where $s$ represents the plot index. A uniform prior was assigned to each plot-specific dispersal probability:

$$p_{\text{disp},s} \sim \text{Uniform}(0,1), \tag{2}$$

which assumes equal prior probability across the entire range of possible dispersal values.

The estimated $p_{\text{disp},s}$ values are shown in Supplementary Table 11.

Additionally, we calculated the mean annual dispersal probability in each area: RFC = 0.046 (95% CRI: 0.023–0.075) and HUI = 0.040 (95% CRI: 0.024–0.059).

# Comparison of dispersal probabilities among plots

To examine differences in dispersal probability across plots within an area, posterior distributions of $p_{\text{disp},s}$ were compared using the `overlapping` package (Pastore 2018) in R. This analysis quantified the degree of overlap between posterior distributions for each pair of plots. The pairwise overlap matrix was computed as:

$$O_{p,q} = 1 - \text{overlap}(p_{\text{disp},p}, p_{\text{disp},q}), \tag{3}$$

where $O_{p,q}$ represents the difference in dispersal probability between plots $p$ and $q$. A value close to zero indicates high similarity in dispersal probabilities, whereas a value near one suggests minimal overlap and distinct dispersal rates.

No significant differences were detected between plots both at RFC (Supplementary Fig. 47) and HUI (Supplementary Fig. 48), indicating that dispersal probability was relatively homogeneous within each area.

# Conclusion

The results indicate that dispersal probability does not vary significantly among plots within RFC nor HUI. Dispersal probability, even considering the multi-year duration of this study, was low in both areas, supporting our assumption that each plot held a local population of *R. darwinii*.

# Supplementary Information: Microbiome

We determined the skin bacteriome (hereafter "microbiome") composition in 857 skin swabs from 397 *Rhinoderma darwinii* individuals captured in RFC (94.7% of the total of individual captured in that area). The skin microbiome composition was evaluated using the same DNA extractions used for *Batrachochytrium dendrobatidis* (Bd) detection. We purified these extractions using the Genomic DNA Clean & Concentrator kit (gDCC, Zymo Research) following the manufacturer's guidelines. To generate sequencing libraries, the V3-V4 hypervariable region of the 16S rRNA gene was amplified and sequenced, employing primers adapted from Klindworth et al. (2013). Library preparation adhered to the Illumina protocol for 16S Metagenomic Sequencing Library, employing 341F and 805R primers with attached Illumina index adapters, as described by Bourlat et al. (2016). The sequencing strategy incorporated elements from Fadrosh et al. (2014), including a linker sequence optimized for Illumina sequencing, index sequences, and a heterogeneity spacer. Amplicon sequencing was conducted using 300 bp paired-end reads on an Illumina MiSeq sequencer at the AUSTRAL-Omics core research facilities of the Universidad Austral de Chile (Valdivia, Chile).

The raw dataset consisted of 50,617,635 paired sequences, ranging from 129 to 120,981 reads (with an average of 52,562 reads/sample). Raw data were demultiplexed using cutadapt V 2.0 (Martin 2011), allowing a maximum of one mismatch in barcode sequences. Demultiplexed reads were subsequently processed to infer Amplicon Sequence Variants (ASVs) using the R package DADA2 (Callahan et al. 2016), following the recommended pipeline, which includes a quality filtering step. For quality filtering, the function FilterAndTrim was utilized with the parameters maxEE = c(2,5), truncLen = c(250,220), trimLeft = c(17,21). Chimeric sequences were identified and removed using the removeBimeraDenovo function in DADA2. The resulting ASVs were then taxonomically classified by mapping them to the SILVA138 bacterial 16S rRNA database (Quast et al. 2012) using the RDP naïve Bayesian classifier (Wang et al. 2007). A manual additional filtering step was performed. Then, ASVs classified as mitochondria or chloroplasts, as well as those with total abundances fewer than 5 reads, were filtered out. After this filtering step, the number of reads ranged from 360 to 39,390 reads (with an average of 15,770 reads per sample). To standardize the sequencing depth across samples, rarefaction was applied, resulting in 5,000 reads per sample.

To obtain the microbiome variables used as predictors of Bd infection probability in the multistate capture-recapture model, we summarised the ASV count table at the family level and calculated Observed Richness, Shannon Diversity Index, and Pielou's Evenness using the phyloseq package in R (McMurdie et al. 2013).

# Supplementary Information: Strength of Evidence

As we used a Bayesian framework for most of the statistical analyses in this study, we were able to quantify the strength of evidence for a difference in a given parameter between pairs of units (e.g., age classes, populations, areas, etc.). Specifically, the strength of evidence is defined as 1 minus the proportion of overlap between the posterior distributions of the parameter for the two units (Pastore and Calcagnì 2019). In other words, it represents the fraction of the posterior distribution that does not overlap. For instance, to assess the strength of evidence for a difference in mean infection probability between populations A and B, we calculate one minus the overlapping proportion of their respective posterior distributions for mean infection probability. This value is typically presented as a percentage. The overlapping proportion between two posterior distributions was computed using the R package overlapping (Pastore 2018). For regression coefficients, we evaluated the strength of evidence for a positive or negative association by calculating the proportion of the posterior distribution of the regression coefficient that was either positive or negative, respectively.

# Supplementary Information: References


Bacigalupe, L. D., Soto-Azat, C., García-Vera, C., Barría-Oyarzo, I.,& Rezende, E. L. (2017). Effects of amphibian phylogeny, climate and human impact on the occurrence of the amphibian-killing chytrid fungus. *Global Change Biology, 23*(9), 3543–3553. https://doi.org/10.1111/gcb.13610

Baddeley, A., Rubak, E., & Turner, R. (2016). *Spatial point patterns: Methodology and applications with R.* CRC Press, Taylor & Francis Group.

Bonner, S. J., Morgan, B. J., & King, R. (2010). Continuous covariates in mark–recapture–recovery analysis: A comparison of methods. *Biometrics, 66*(4), 1256–1265. https://doi.org/10.1111/j.1541-0420.2010.01385.x

Bourlat, S. J., Haenel, Q., Finnman, J., & Leray, M. (2016). Preparation of amplicon libraries for metabarcoding of marine eukaryotes using Illumina MiSeq: The dual-PCR method. In S. J. Bourlat (Ed.), *Marine Genomics* (Vol. 1452, pp. 197–207). Springer New York. https://doi.org/10.1007/978-1-4939-3774-5_13

Burns, T. J., Scheele, B. C., Brannelly, L. A., Clemann, N., Gilbert, D., & Driscoll, D. A. (2021). Indirect terrestrial transmission of amphibian chytrid fungus from reservoir to susceptible host species leads to fatal chytridiomycosis. *Animal Conservation, 24*(4), 602–612. https://doi.org/10.1111/acv.12665

Callahan, B. J., McMurdie, P. J., Rosen, M. J., Han, A. W., Johnson, A. J. A., & Holmes, S. P. (2016). DADA2: High-resolution sample inference from Illumina amplicon data. *Nature Methods, 13*(7), 581–583. https://doi.org/10.1038/nmeth.3869

Cayuela, H., Rougemont, Q., Prunier, J. G., Moore, J. S., Clobert, J., Besnard, A., & Bernatchez, L. (2018). Demographic and genetic approaches to study dispersal in wild animal populations: A methodological review. *Molecular Ecology, 27*(20), 3976–4010.

Chamberlain, S. (2023). *iucnredlist: 'IUCN' Red List Client* (R package version 0.7.0). https://docs.ropensci.org/iucnredlist/

Fadrosh, D. W., Ma, B., Gajer, P., Sengamalay, N., Ott, S., Brotman, R. M., & Ravel, J. (2014). An improved dual-indexing approach for multiplexed 16S rRNA gene sequencing on the Illumina MiSeq platform. *Microbiome, 2*(1), 6. https://doi.org/10.1186/2049-2618-2-6

Ferrari, M. J., Bjørnstad, O. N., & Dobson, A. P. (2005). Estimation and inference of $R_0$ of an infectious pathogen by a removal method. *Mathematical Biosciences, 198*(1), 14–26.

Foufopoulos, J., Wobeser, G. A., & McCallum, H. (2022). *Infectious disease ecology and conservation*. Oxford University Press.

Gimenez, O., Cam, E., & Gaillard, J. M. (2018). Individual heterogeneity and capture–recapture models: What, why and how? *Oikos, 127*(5), 664–686. https://doi.org/10.1111/oik.04773

Hanlon, S., Henson, J., & Kerby, J. (2017). Detection of amphibian chytrid fungus on waterfowl integument in natural settings. *Diseases of Aquatic Organisms, 126*(1), 71–74. https://doi.org/10.3354/dao03160

Harper, E. B., Stella, J. C., & Fremier, A. K. (2011). Global sensitivity analysis for complex ecological models: A case study of riparian cottonwood population dynamics. *Ecological Applications, 21*(4), 1225–1240. https://doi.org/10.1890/10-0384.1

Kellner, K. (2015). *jagsUI: A wrapper around 'rjags' to streamline 'JAGS' analyses*. R package version 1.3.7. http://CRAN.R-project.org/package=jagsUI

Kéry, M., & Schaub, M. (2012). *Bayesian population analysis using WinBUGS: A hierarchical perspective*. Academic Press.

Klindworth, A., Pruesse, E., Schweer, T., Peplies, J., Quast, C., Horn, M., & Glöckner, F. O. (2013). Evaluation of general 16S ribosomal RNA gene PCR primers for classical and next-generation sequencing-based diversity studies. *Nucleic Acids Research, 41*(1), e1. https://doi.org/10.1093/nar/gks808

Kolby, J. E., Ramirez, S. D., Berger, L., Griffin, D. W., Jocque, M., & Skerratt, L. F. (2015). Presence of amphibian chytrid fungus (*Batrachochytrium dendrobatidis*) in rainwater suggests aerial dispersal is possible. *Aerobiologia, 31*(3), 411–419. https://doi.org/10.1007/s10453-015-9374-6

Langrock, R., & King, R. (2013). Maximum likelihood estimation of mark–recapture–recovery models in the presence of continuous covariates. *Annals of Applied Statistics, 7*(3), 1709–1732. https://doi.org/10.1214/13-AOAS645

Liaw, A., & Wiener, M. (2002). Classification and regression by randomForest. *R News, 2*(3), 18–22. https://CRAN.R-project.org/doc/Rnews/

Martin, M. (2011). Cutadapt removes adapter sequences from high-throughput sequencing reads. *EMBnet.Journal, 17*(1), 10. https://doi.org/10.14806/ej.17.1.200

McMurdie, P. J., & Holmes, S. (2013). phyloseq: An R package for reproducible interactive analysis and graphics of microbiome census data. *PLoS ONE, 8*(4), e61217. https://doi.org/10.1371/journal.pone.0061217

Müller, H., Liedtke, H. C., Menegon, M., Beck, J., Ballesteros-Mejia, L., Nagel, P., & Loader, S. P. (2013). Forests as promoters of terrestrial life-history strategies in East African amphibians. *Biology Letters, 9*(3), 20121146. https://doi.org/10.1098/rsbl.2012.1146

Nowakowski, A. J., Thompson, M. E., Donnelly, M. A., & Todd, B. D. (2017). Amphibian sensitivity to habitat modification is associated with population trends and species

traits. *Global Ecology and Biogeography, 26*(6), 700–712. https://doi.org/10.1111/geb.12574

Nunes-de-Almeida, C. L., Haddad, C. F. B., & Toledo, L. F. (2021). A revised classification of the amphibian reproductive modes. *Salamandra, 57*(3), 413–427.

Pastore, M. (2018). Overlapping: An R package for estimating overlapping in empirical distributions. *Journal of Open Source Software, 3*(32), 1023. https://doi.org/10.21105/joss.01023

Pastore, M., & Calcagnì, A. (2019). Measuring distribution similarities between samples: A distribution-free overlapping index. *Frontiers in Psychology, 10*, 1089. https://doi.org/10.3389/fpsyg.2019.01089

Plummer, M. (2003). JAGS: A program for analysis of Bayesian graphical models using Gibbs sampling. In *Proceedings of the 3rd International Workshop on Distributed Statistical Computing* (pp. 1–10). Vienna, Austria.

Prado, J. S., Ernetti, J. R., Pontes, M. R., & Toledo, L. F. (2023). Chytrid in the clouds: An alternative passive transport of a lethal pathogen for amphibians. *Hydrobiologia, 850*(9), 2061–2073. https://doi.org/10.1007/s10750-023-05218-2

Quast, C., Pruesse, E., Yilmaz, P., Gerken, J., Schweer, T., Yarza, P., Peplies, J., & Glöckner, F. O. (2012). The SILVA ribosomal RNA gene database project: Improved data processing and web-based tools. *Nucleic Acids Research, 41*(D1), D590–D596. https://doi.org/10.1093/nar/gks1219

R Core Team. (2021). *R: A language and environment for statistical computing.* R Foundation for Statistical Computing, Vienna, Austria. https://www.R-project.org/

Rabanal, F., & Nuñez, J. J. (2008). *Anfibios de los bosques templados de Chile.* Universidad Austral de Chile.

Schaub, M., & Royle, J. A. (2014). Estimating true survival using spatial Cormack–Jolly–Seber models. *Methods in Ecology and Evolution, 4*(2), 146–155. https://doi.org/10.1111/2041-210X.12006

Silk, M. J., Croft, D. P., Delahay, R. J., Hodgson, D. J., Boots, M., Weber, N., & McDonald, R. A. (2017). Using social network measures in wildlife disease ecology, epidemiology, and management. *BioScience, 67*(3), 245–257. https://doi.org/10.1093/biosci/biw175

Stegen, G., et al. (2017). Drivers of salamander extirpation mediated by *Batrachochytrium salamandrivorans*. *Nature, 544*(7650), 353–356. https://doi.org/10.1038/nature22059

Valenzuela-Sánchez, A., et al. (2014). Home range and social analyses in a mouth brooding frog. *Journal of Zoology, 294*(4), 215–223. https://doi.org/10.1111/jzo.12165

Valenzuela-Sánchez, A., et al. (2017). Cryptic disease-induced mortality. *Proc. R. Soc. B, 284*(1863), 20171176. https://doi.org/10.1098/rspb.2017.1176

Wang, Q., et al. (2007). Naïve Bayesian classifier. *Appl. Environ. Microbiol., 73*(16), 5261–5267. https://doi.org/10.1128/AEM.00062-07

Wilber, M. Q., et al. (2017). Resistance, tolerance, and environmental transmission. *Ecology Letters, 20*(9), 1169–1181.

# Supplementary Figures

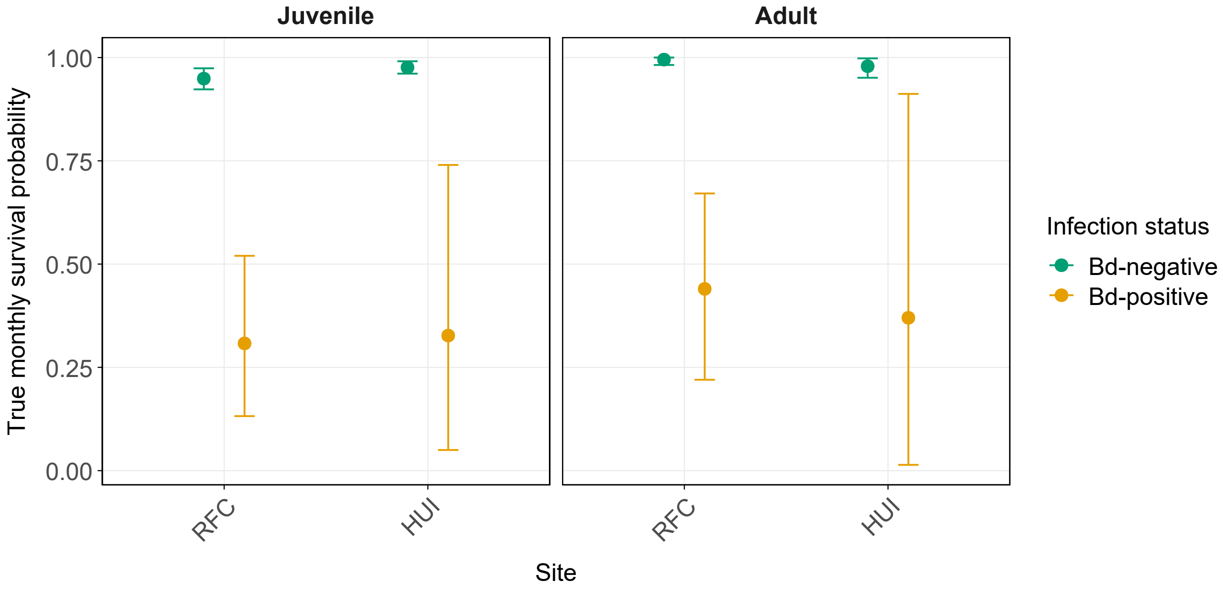


**Supplementary Figure** 1: Monthly survival probability of *Rhinoderma darwinii* in *Batrachochytrium dendrobatidis*-infected and uninfected juveniles and adults. Data were collected from two spatially structured populations (RFC and MNC; corresponding to Field Study 1 in the main manuscript). Survival probabilities reflect true survival as estimated using a spatial multistate capture-recapture model. Estimates are based on capture–recapture data from 758 *R. darwinii* individuals (RFC: 419; HUI: 339). The wider error bars for infected individuals reflect lower sample sizes relative to uninfected individuals (number of captures of infected individuals: RFC = 76, HUI = 13). Points represent posterior means, and error bars represent the Bayesian 95% credible intervals.

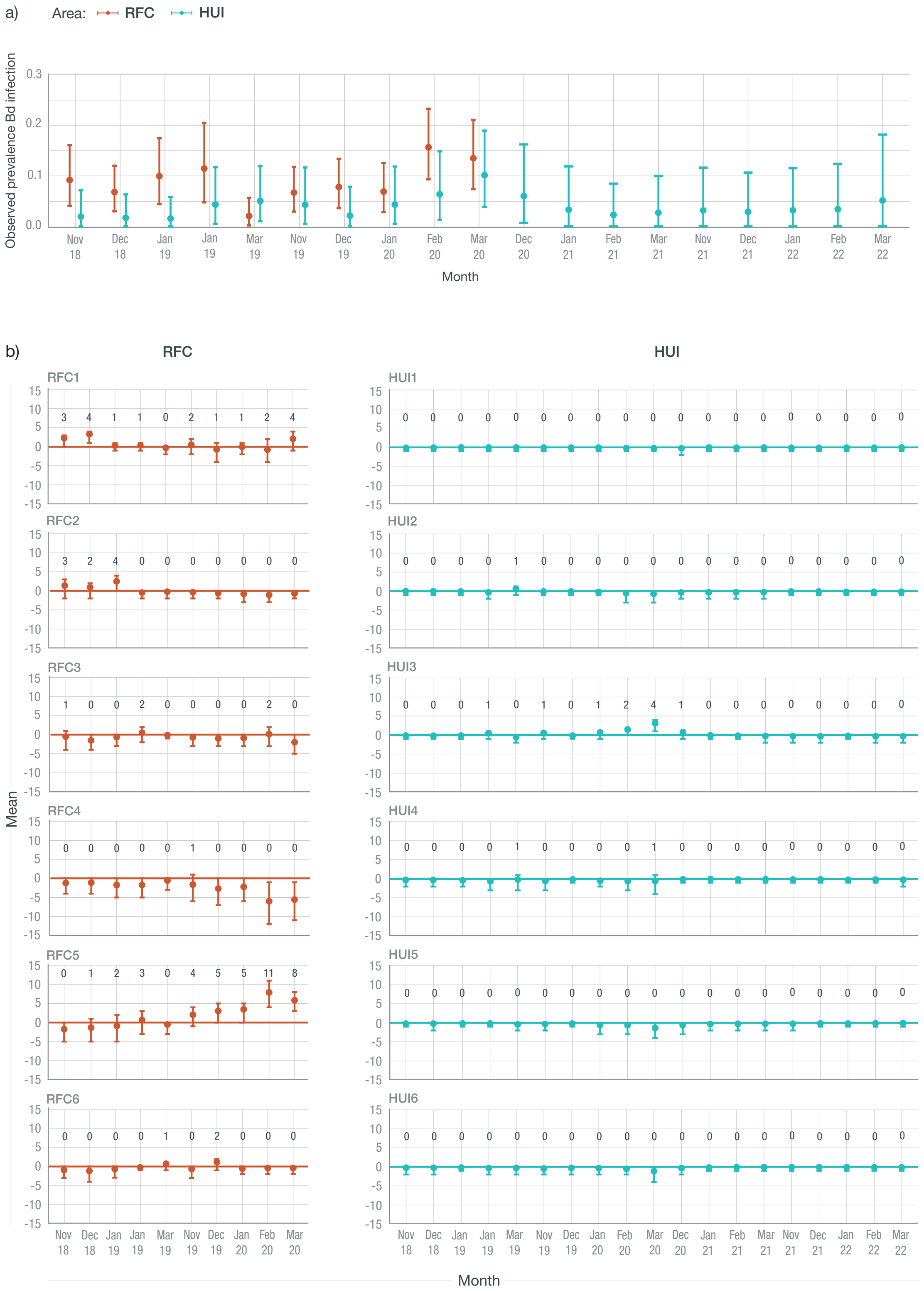


**Supplementary Figure** 2: Area-level prevalence of *Batrachochytrium dendrobatidis* infection (a) and difference between the observed and expected number of Bd-infected individuals in local populations (b) in *Rhinoderma darwinii* in two geographical areas in southern Chile. All parameters were estimated using Bayesian models fitted to data from 758 *R. darwinii* individuals (RFC: 419; HUI: 339). The numbers inside plots in (b) represent the number of Bd-infected individuals detected each month. Points represent posterior means, and error bars represent the Bayesian 95% credible intervals.

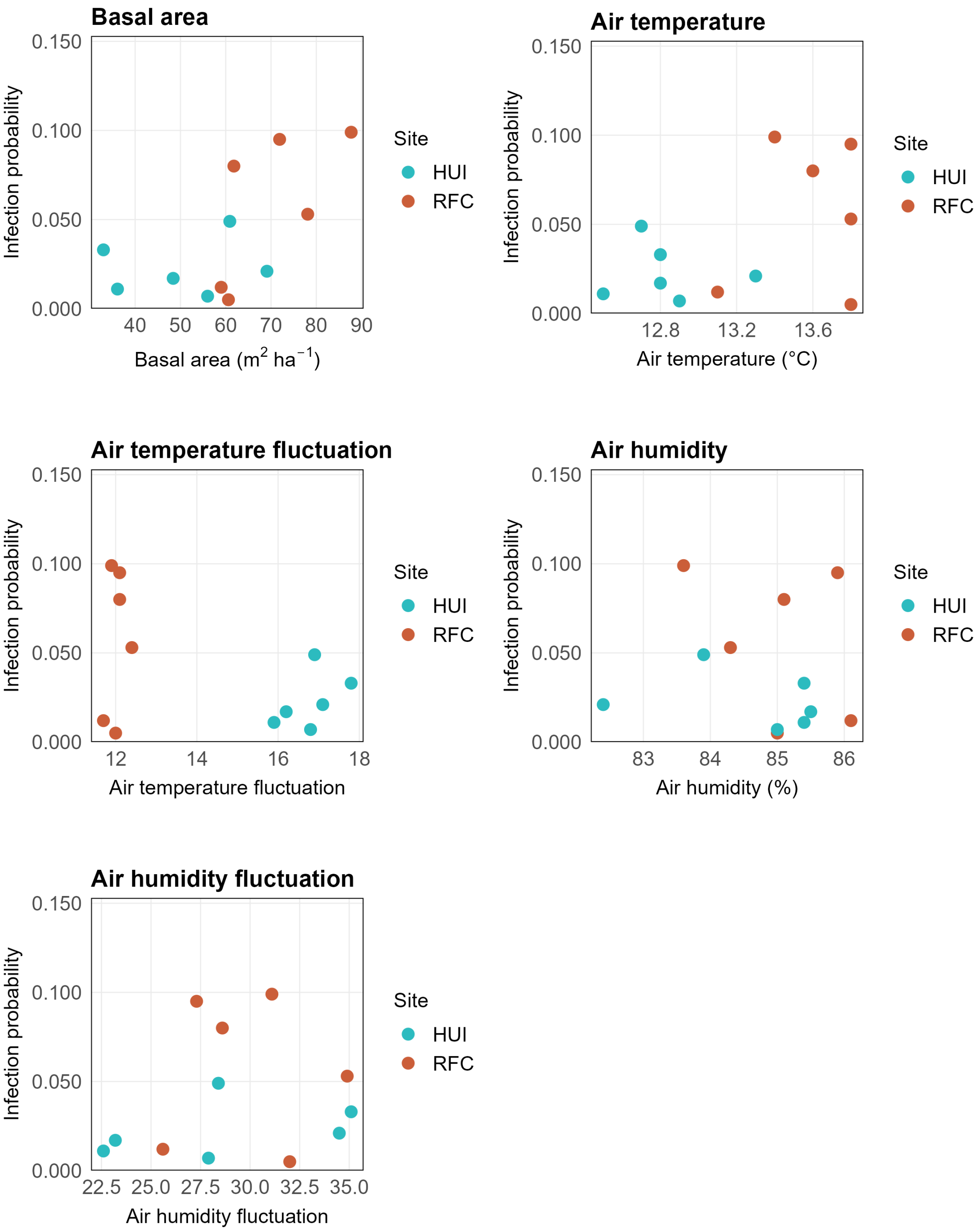


**Supplementary Figure** 3: Relationship between plot-level environmental variables and *Batrachochytrium dendrobatidis* infection probability in *Rhinoderma darwinii* from RFC and HUI. Temperature and humidity values represent posterior means from a Bayesian linear model fitted to monthly site-level variables (either the average or the fluctuation in each variable, the latter defined as the difference between the 99th and 1st percentiles of values recorded during each monthly sampling period). In each area, measurements were taken over approximately five consecutive days per month, totalling about 38 sampling days spread across eight months. Raw data comprised 21,582 individual records per variable (RFC: 9,714; HUI: 11,868), collected across six plots in each area.

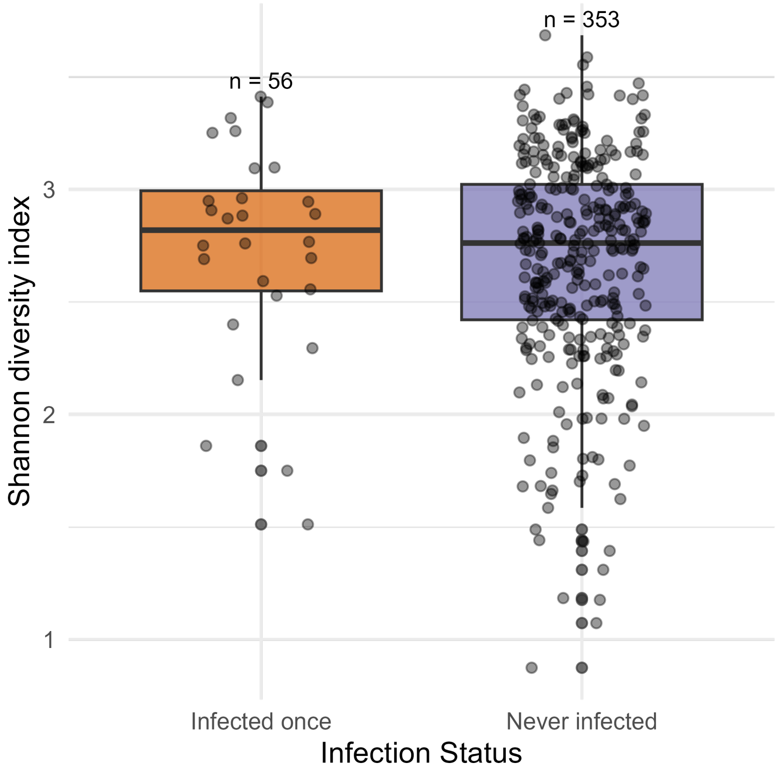


**Supplementary Figure** 4: Shannon diversity index of bacterial families on the skin of *Rhinoderma darwinii* individuals that were either captured once as infected with *Batrachochytrium dendrobatidis* or never observed infected. The plotted value is the average from all months where the individual was captured, including only months previous infection. A linear model indicated the small difference observed between groups was not statistically significant (p-value: 0.595). Boxplots show the median (horizontal line), interquartile range (box), and whiskers extending to the most extreme values within 1.5× the interquartile range from the box.

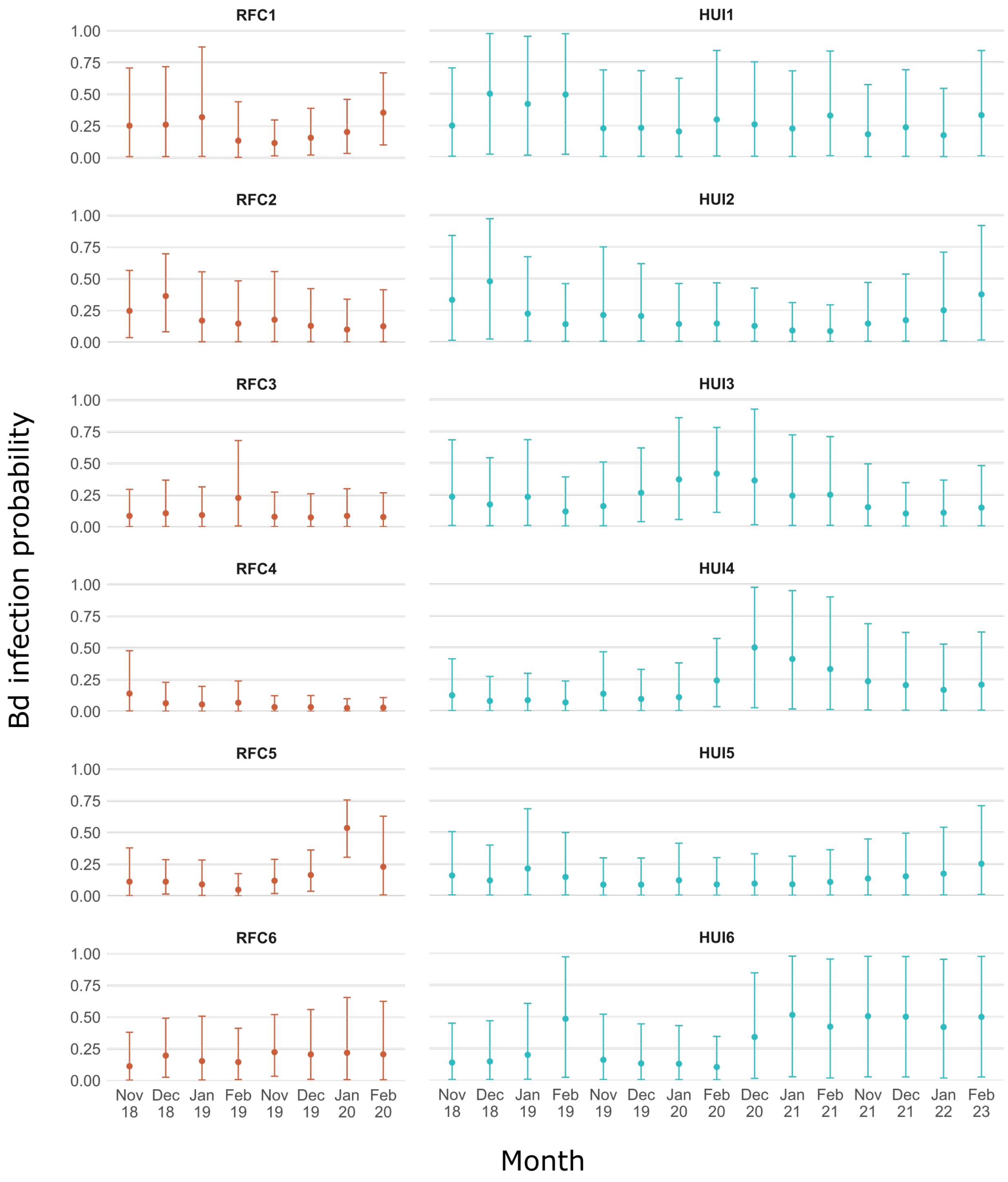


**Supplementary Figure** 5: Subpopulation-level monthly *Batrachochytrium dendrobatidis* infection probability in *Rhinoderma darwinii* from RFC and HUI, estimated using a spatial multistate capture-recapture model. Estimates are based on capture–recapture data from 758 *R. darwinii* individuals (RFC: 419; HUI: 339). Points represent posterior means, and error bars represent the Bayesian 95% credible intervals.

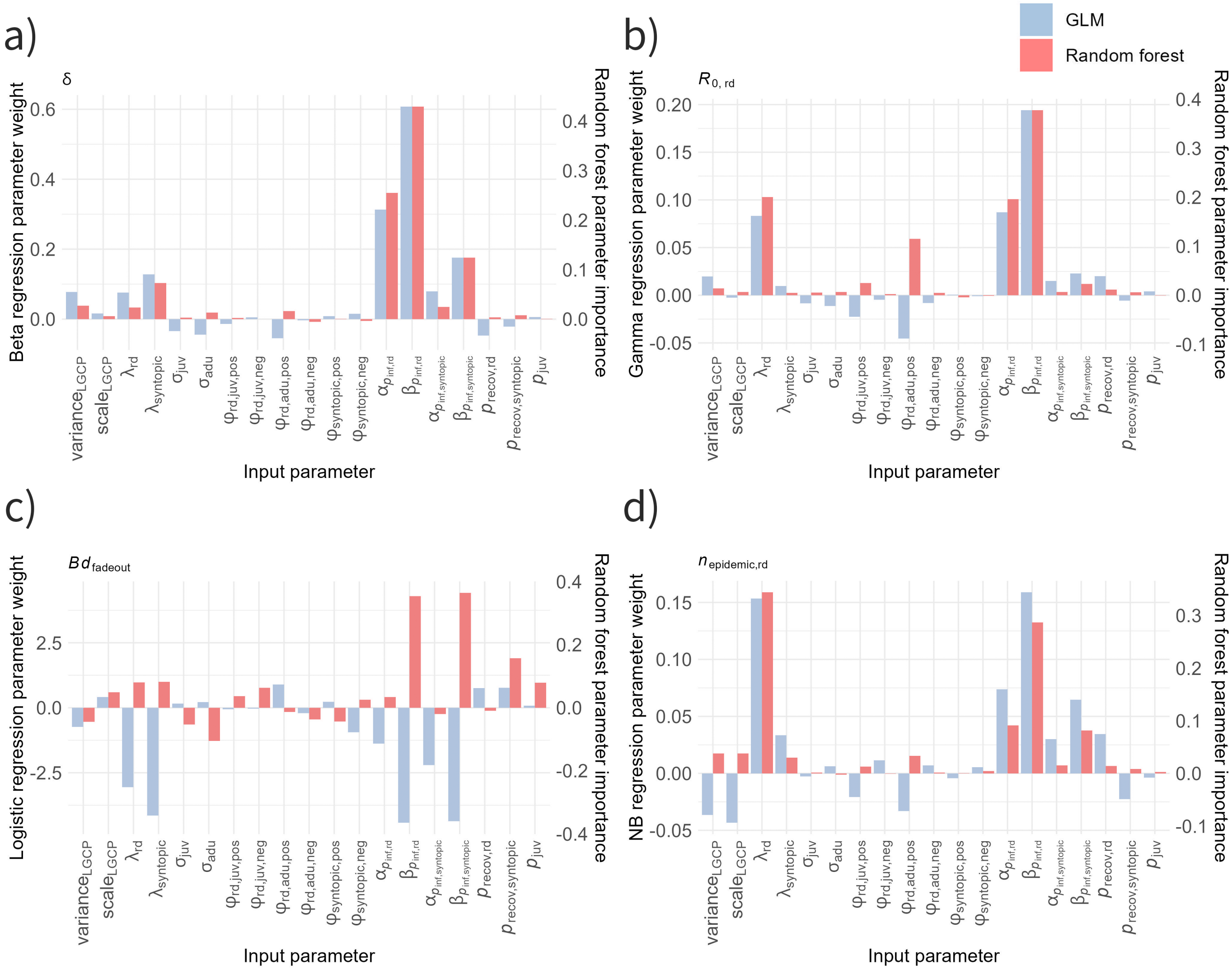


**Supplementary Figure** 6: Relative importance of 19 input parameters on $\delta$ (a), $R_0$ (b), $Bd_{\text{fadeout}}$ (c), and $n_{\text{epidemic,rd}}$ (d) in the global sensitivity analysis of the spatial individual-based model.

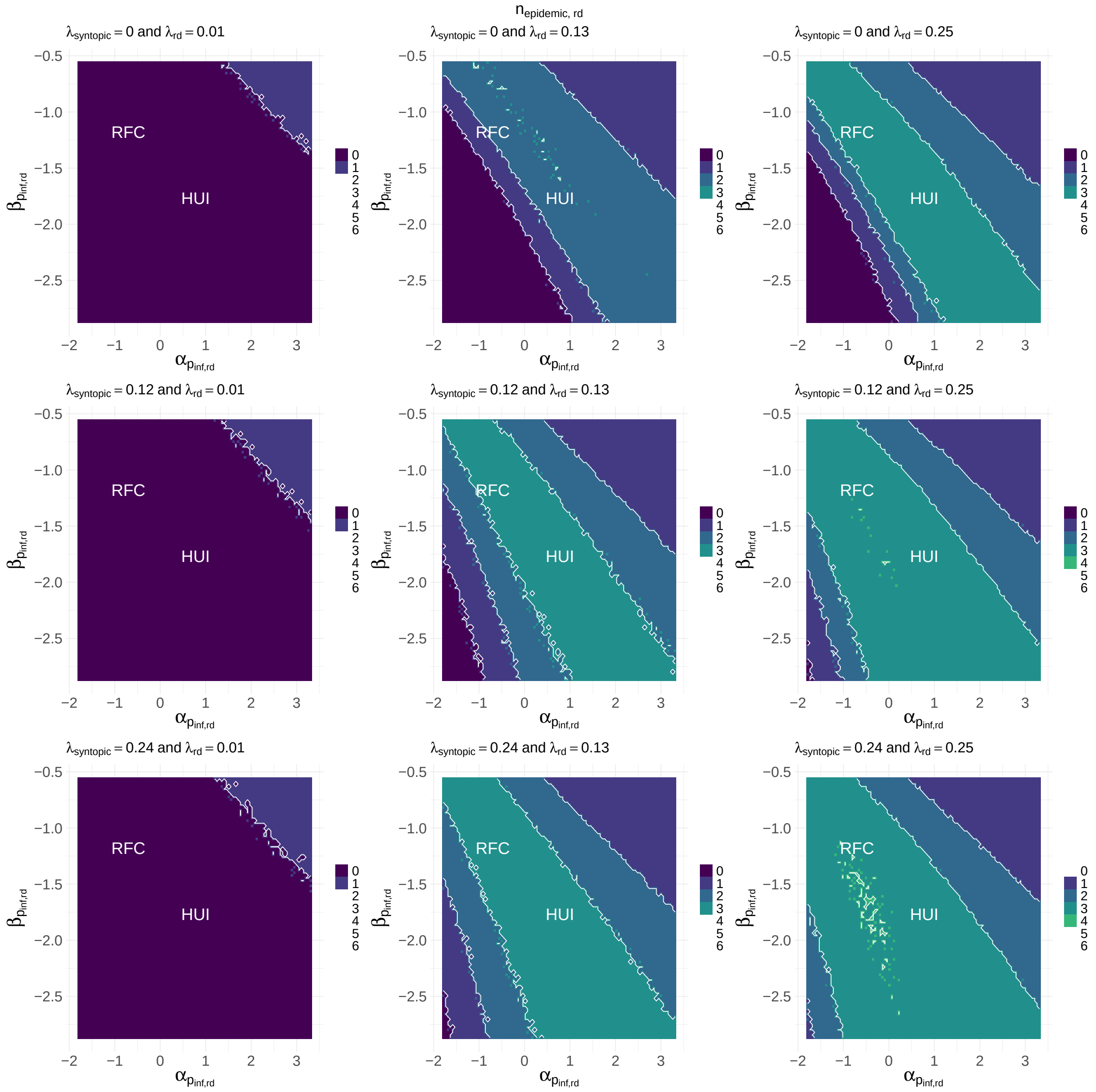


**Supplementary Figure** 7: Number of months with an epidemic ratio $\mathbf{R}_{t,\mathrm{rd}} > 1$ under different combinations of infection parameters and densities of *Rhinoderma darwinii* and a tolerant host species, predicted by a spatial individual-based model. $\alpha_{p_{\mathrm{inf,rd}}}$ represents the intercept and $\beta_{p_{\mathrm{inf,rd}}}$ the regression slope of a logistic regression modelling Bd infection probability as a function of distance to an infected individual. $\lambda_{\mathrm{rd}}$ and $\lambda_{\mathrm{syntopic}}$ denote the density (frogs $m^{-2}$) of *Rhinoderma darwinii* and the tolerant host, respectively. The results represent the median value from 1,000 simulations for each parameter combination. Contour lines indicate integer transitions.

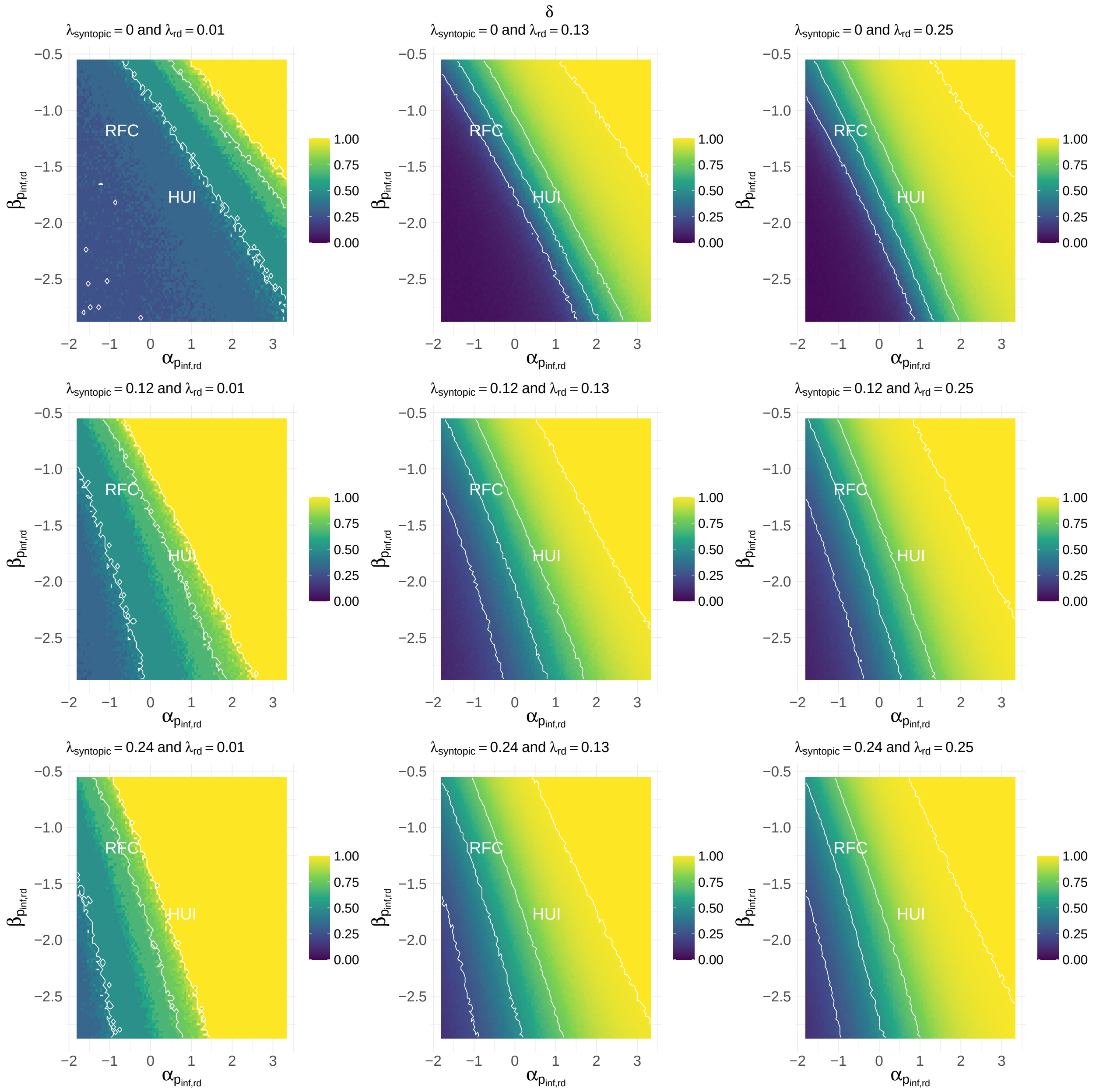


**Supplementary Figure** 8: *Batrachochytrium dendrobatidis*-driven population depression, $\delta$, in *Rhinoderma darwinii* under different combinations of infection parameters and densities of *Rhinoderma darwinii* and a tolerant host species, predicted by a spatial individual-based model. A value of $\delta = 0$ indicates no population depression (i.e., the population size when Bd is present is equal to that when Bd is absent), whereas a value close to 1 indicates high Bd-induced mortality. In the extreme case, $\delta = 1$ if the population with Bd becomes extinct. $\alpha_{p_{\text{inf,rd}}}$ represents the intercept and $\beta_{p_{\text{inf,rd}}}$ the regression slope of a logistic regression modelling Bd infection probability as a function of distance to an infected individual. $\lambda_{\text{rd}}$ and $\lambda_{\text{syntopic}}$ denote the density (frogs $m^{-2}$) of *Rhinoderma darwinii* and the tolerant host, respectively. The results represent the median value from 1,000 simulations for each parameter combination. Contour lines indicate $\delta$ values of 0.25, 0.5, 0.75 and 1.

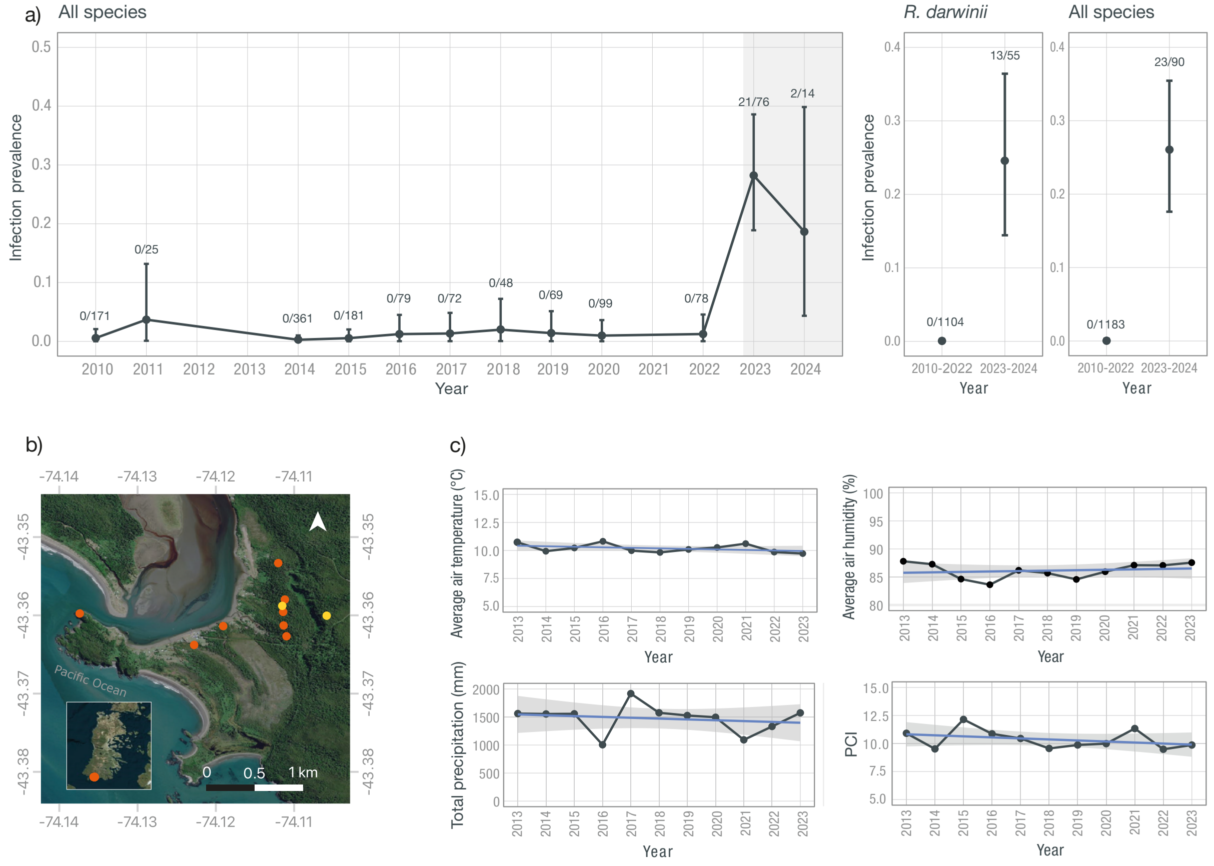


**Supplementary Figure** 9: (a) Prevalence of Bd infection in amphibians in Inio, Tantauco Park, from 2010 to 2024 (left panel) and grouped by the period before the first detection of Bd (i.e., 2010 to 2022) and when Bd was present (i.e., 2023 and 2024) (right panel). The numbers represent no. Bd(+) individuals/no. individuals sampled. (b) Map of the study area, showing sites where amphibians were sampled for Bd infection during 2010, 2011, and 2014 (orange circles). From 2015 to 2024, only sites TAN1 and TAN2 (yellow circles) were monitored for Bd infections in *Rhinoderma darwinii* and syntopic amphibians. (c) Climatic data recorded at a weather station located in Chiloé Island, approximately 75 km from the monitored populations. PCI stands for the "Precipitation Concentration Index", which was calculated on a daily precipitation series. Monthly climatic data (average air temperature, average humidity, and total precipitation) from 2013 to 2023 was obtained from a weather station located in Tara, Chonchi (Red Agrometeorológica de INIA, www.agrometeorologia.cl) approximately 75 km from Inio. We also calculated the Precipitation Concentration Index (PCI) using the R package precincton on a daily precipitation series for the same period of time. Error bars represent the Bayesian 95% credible intervals.

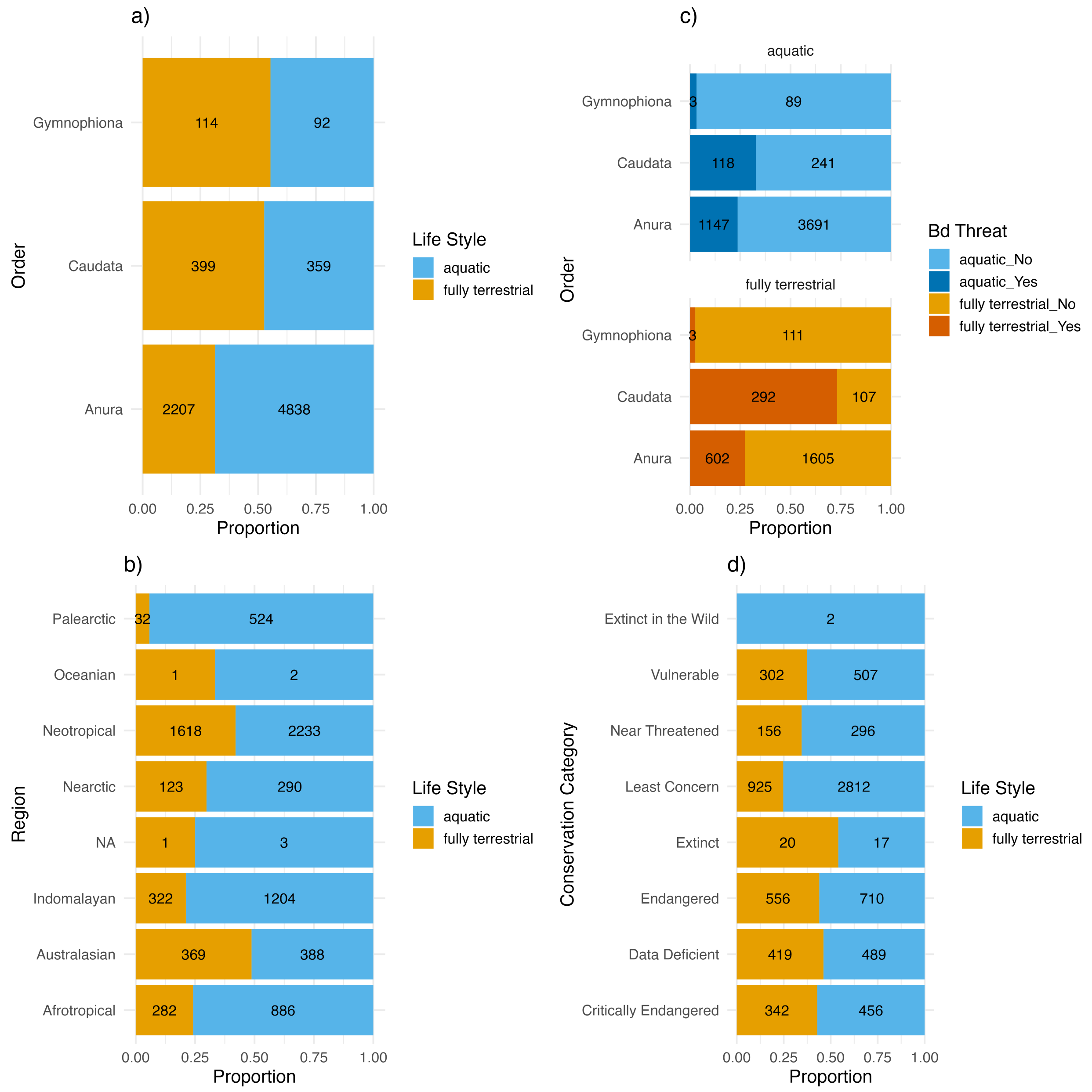


**Supplementary Figure** 10: Global patterns of amphibian lifestyle based on data from the IUCN Red List. a) Proportion of aquatic vs fully terrestrial amphibians across taxonomic orders. b) Distribution of lifestyles across biogeographic realms. c) Proportion of species within each amphibian order for which *Batrachochytrium dendrobatidis* (Bd) is listed as a threat in the IUCN Red List. d) Proportion of lifestyles by IUCN conservation category. The numbers represent the total number of species in each category

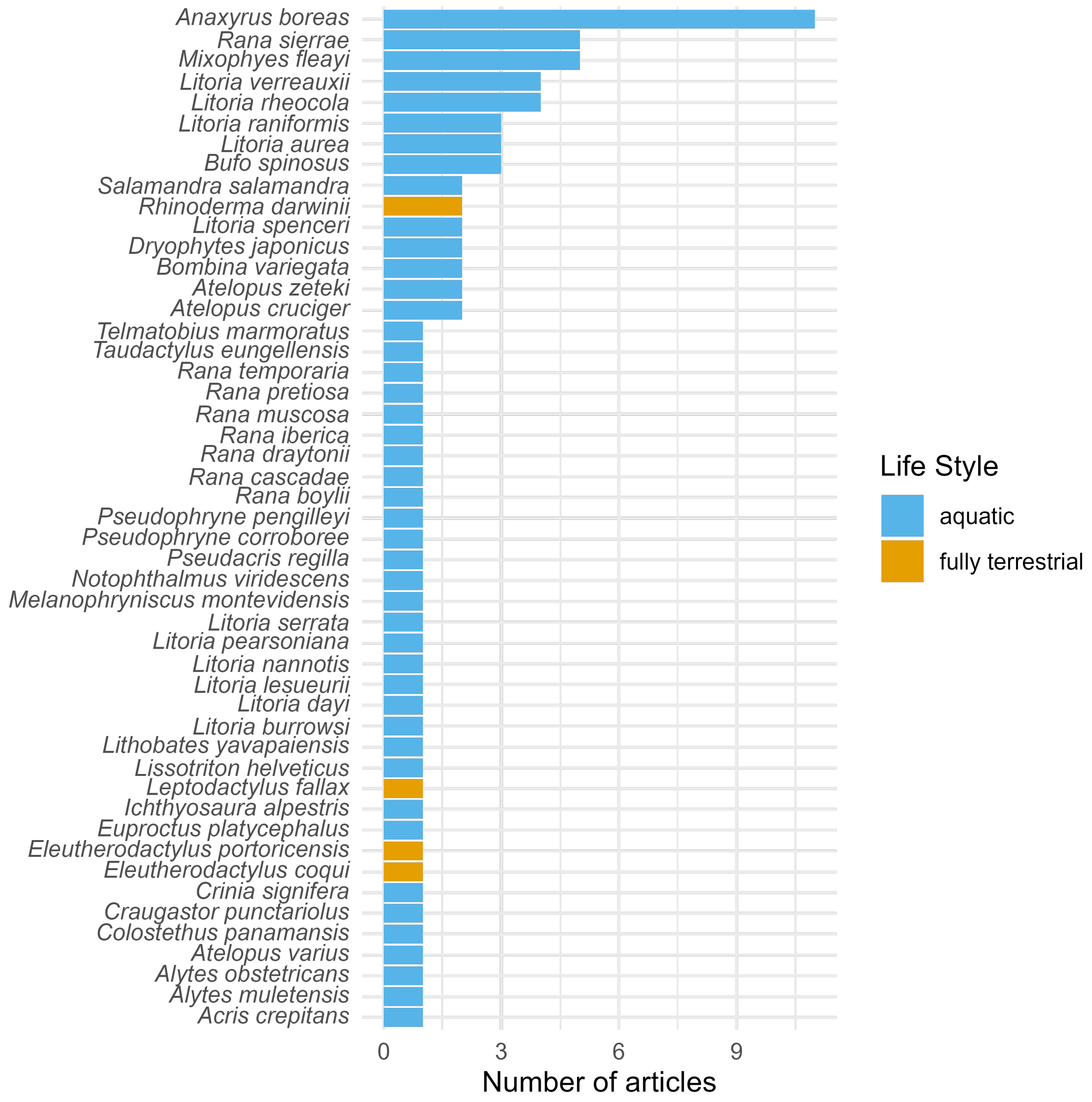


**Supplementary Figure** 11: Number of scientific articles evaluating the impact of *Batrachochytrium dendrobatidis* on amphibian species. Bars indicate the number of independent studies per species, and the colour of the bar represent the lifestyle of the species

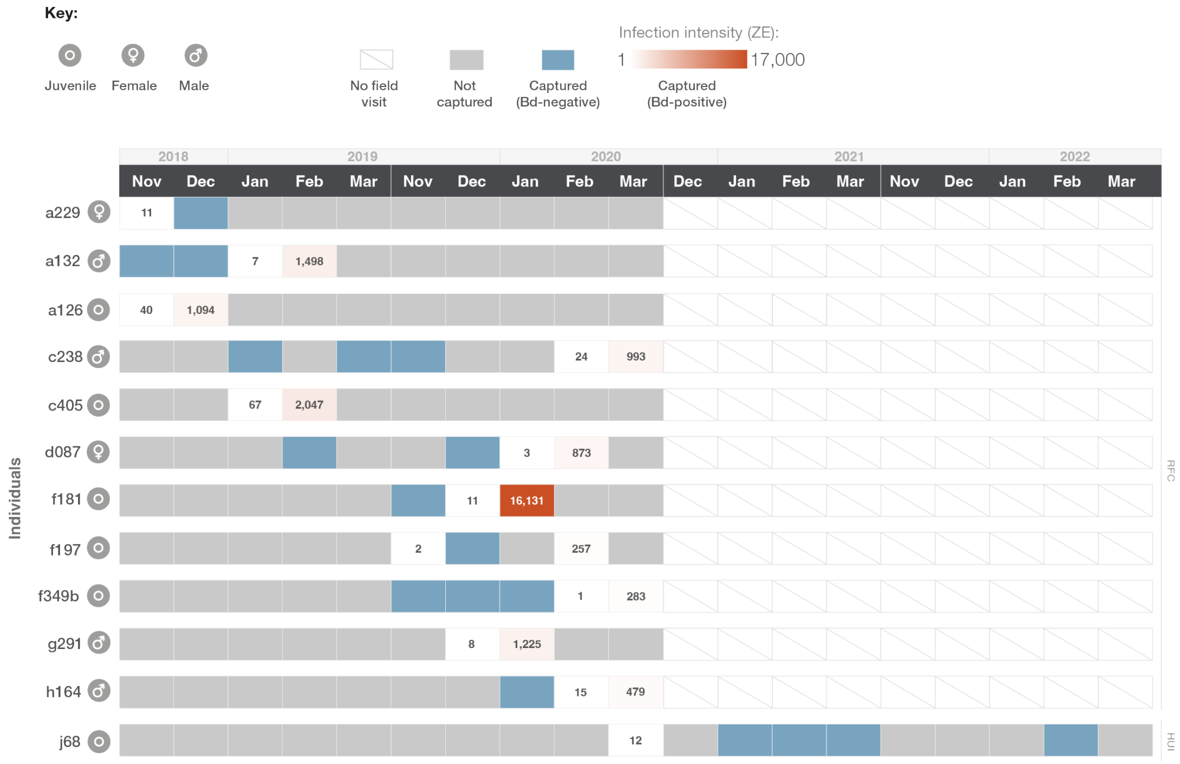


**Supplementary Figure** 12: Capture-recapture history of *Rhinoderma darwinii* individuals captured as Bd-positive and recaptured at least once in subsequent months in RFC and HUI.

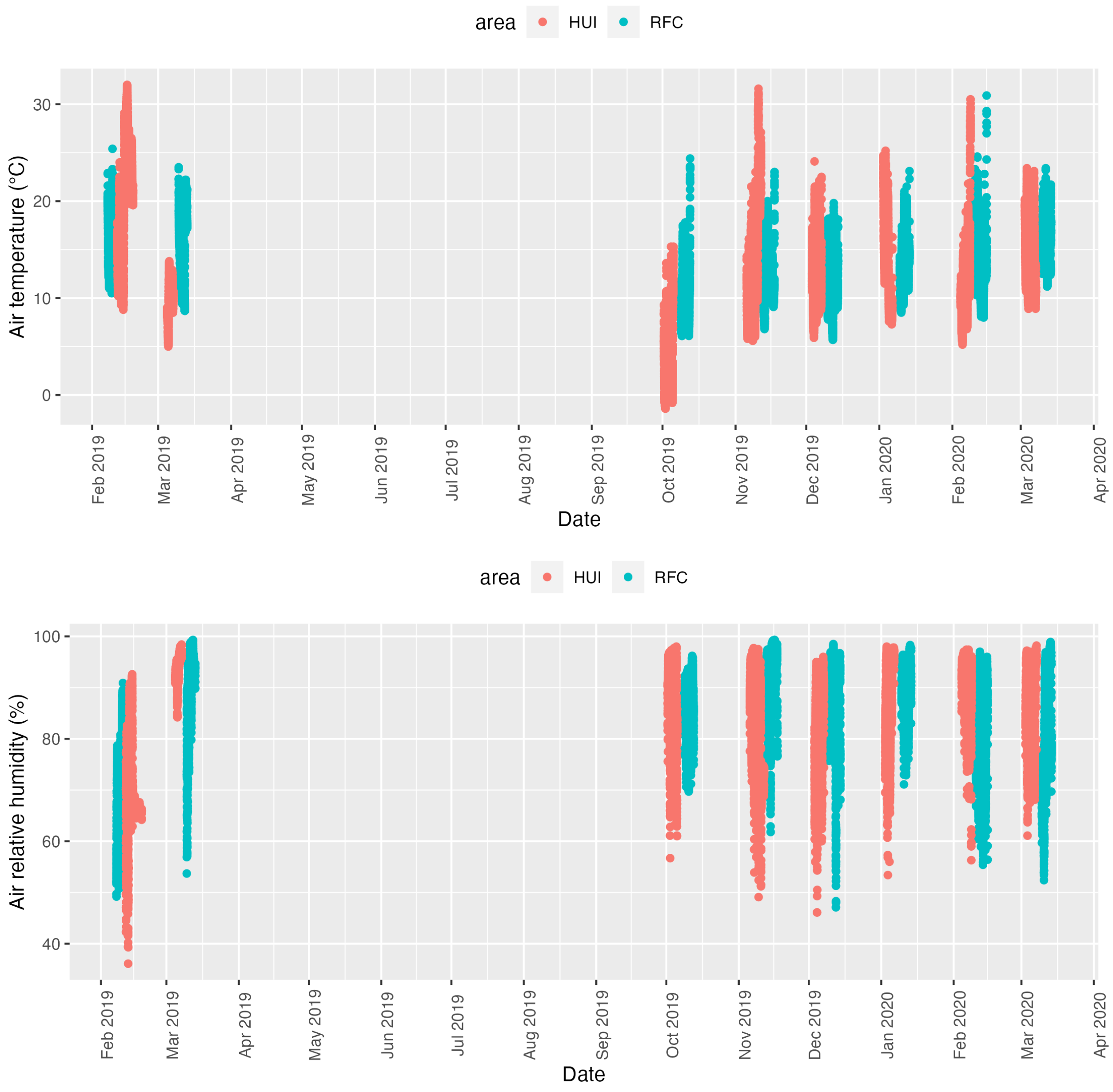


**Supplementary Figure** 13: Raw microclimatic data measured in Field Study 1.

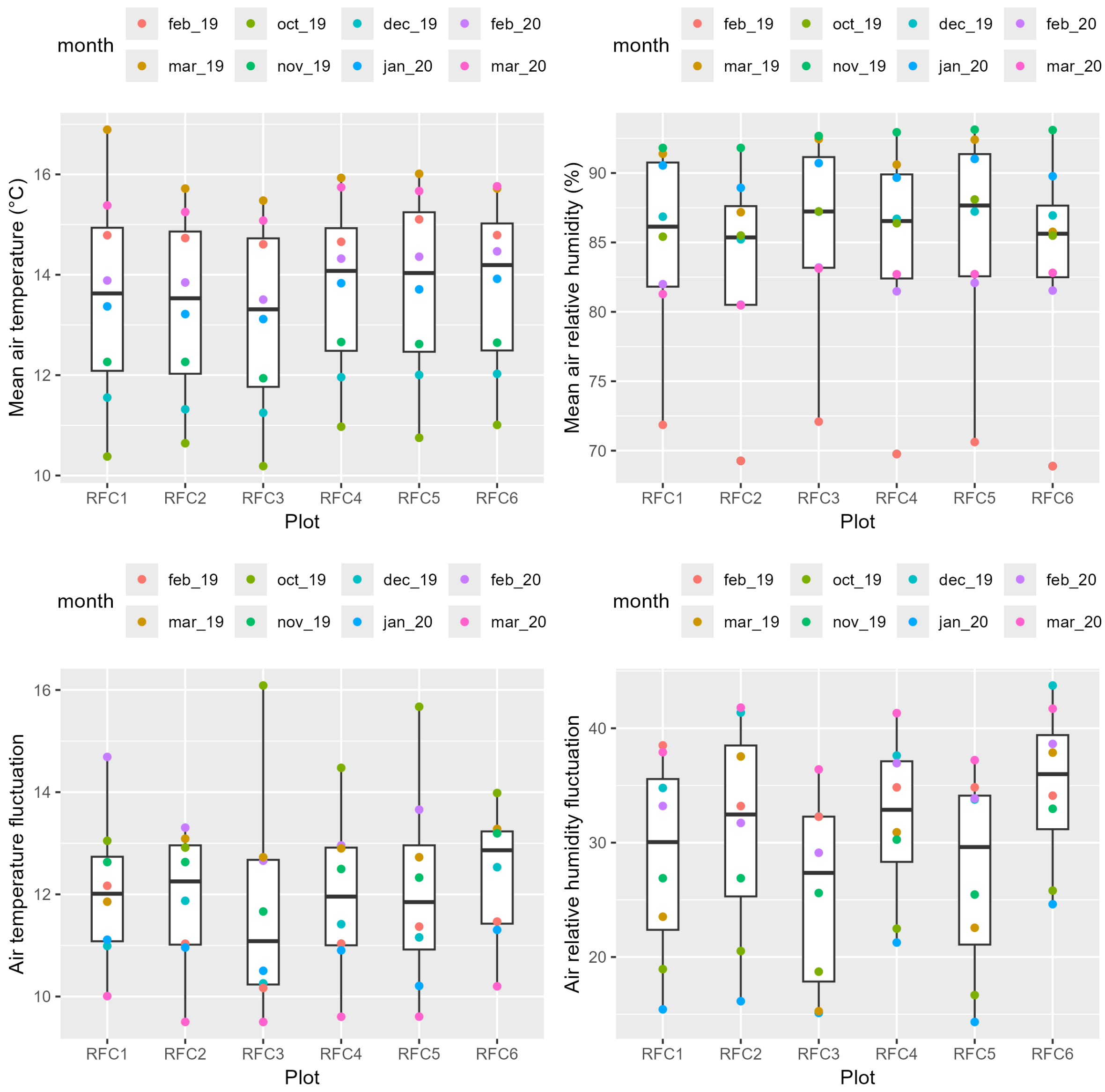


**Supplementary Figure** 14: Temporal and spatial variation in microclimatic variables measured at the plot-level (subpopulation) in Field Study 1 in RFC. Boxplots show the median (horizontal line), interquartile range (box), and whiskers extending to the most extreme values within 1.5× the interquartile range from the box, for either the mean or the fluctuation in each variable per sampling month (the latter defined as the difference between the 99th and 1st percentiles of values recorded during each monthly sampling period). Measurements were taken over approximately four consecutive days per month, totalling about 34 sampling days distributed across eight months. Raw data comprised 9,714 individual records per variable, collected across six plots.

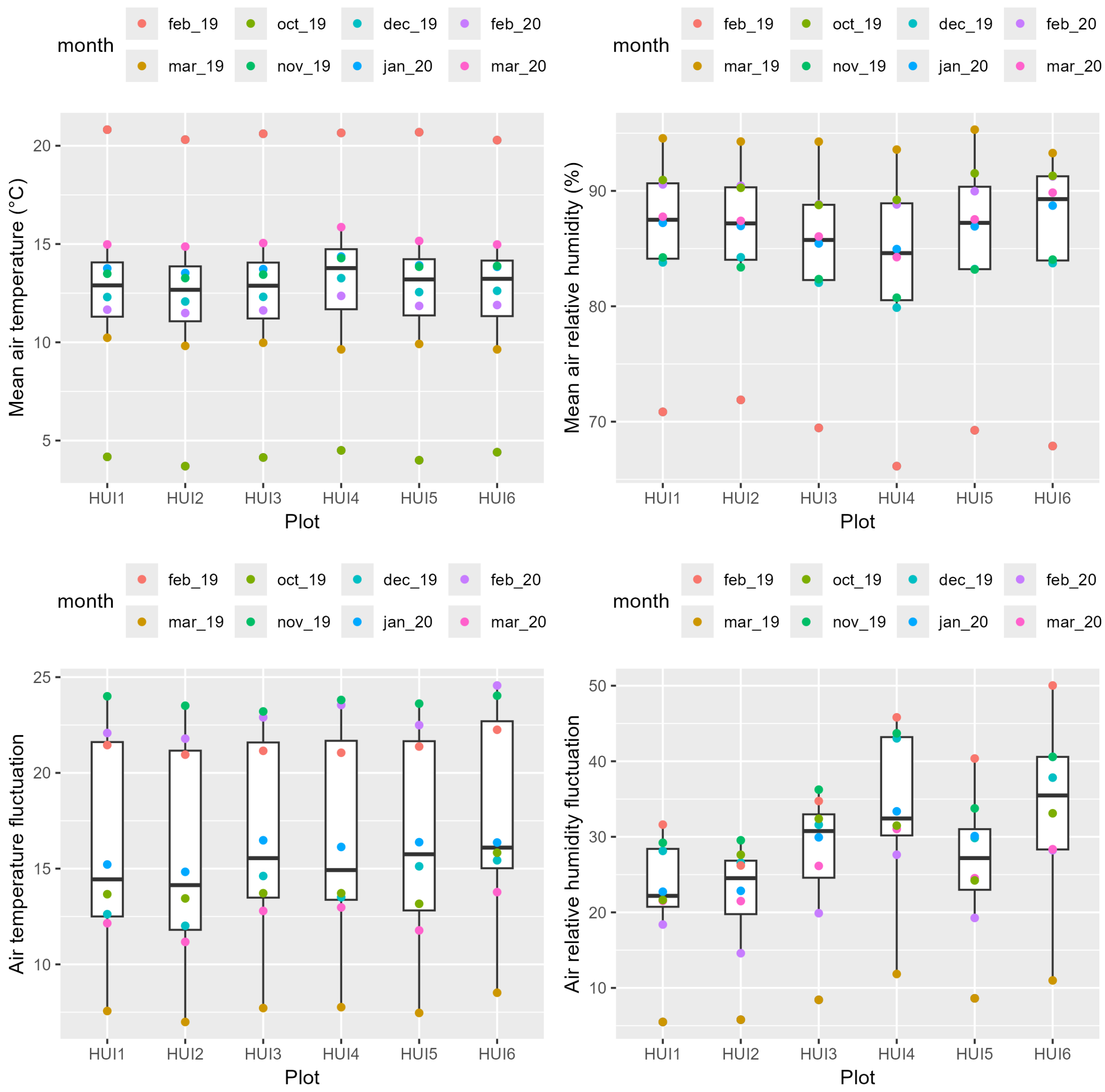


**Supplementary Figure** 15: Temporal and spatial variation in microclimatic variables measured at the plot-level (subpopulation) in Field Study 1 in HUI. Boxplots show the median (horizontal line), interquartile range (box), and whiskers extending to the most extreme values within 1.5× the interquartile range from the box, for either the mean or the fluctuation in each variable per sampling month (the latter defined as the difference between the 99th and 1st percentiles of values recorded during each monthly sampling period). Measurements were taken over approximately five consecutive days per month, totalling about 41 sampling days distributed across eight months. Raw data comprised 11,868 individual records per variable, collected across six plots.

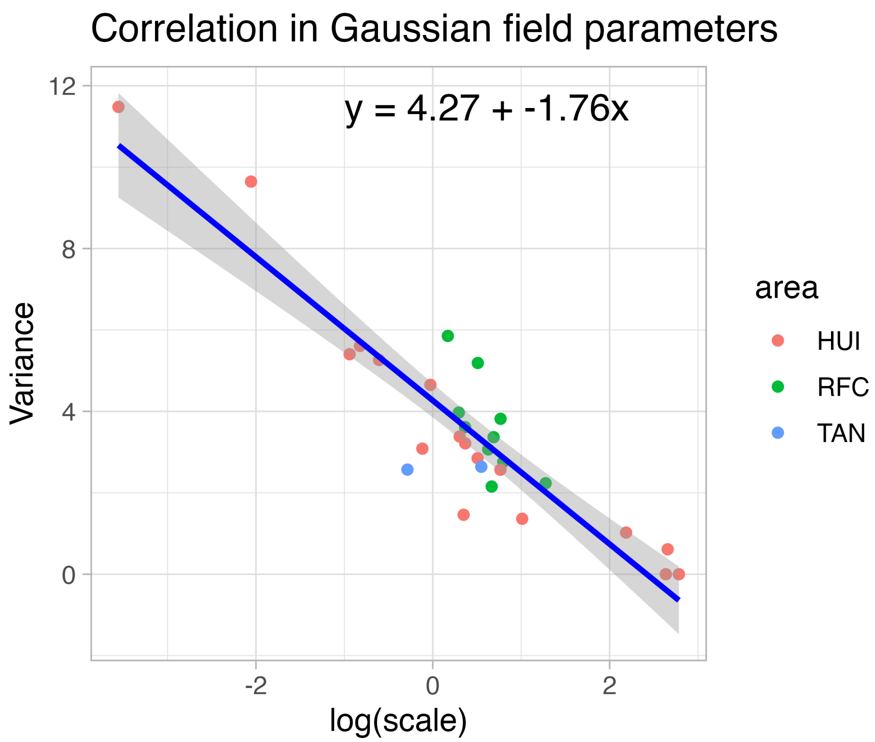


**Supplementary Figure** 16: Correlation in Gaussian field parameters from a log-Gaussian Cox process model with an exponential covariance function fitted to spatial distribution data of *Rhinoderma darwinii* in three geographical areas in southern Chile.

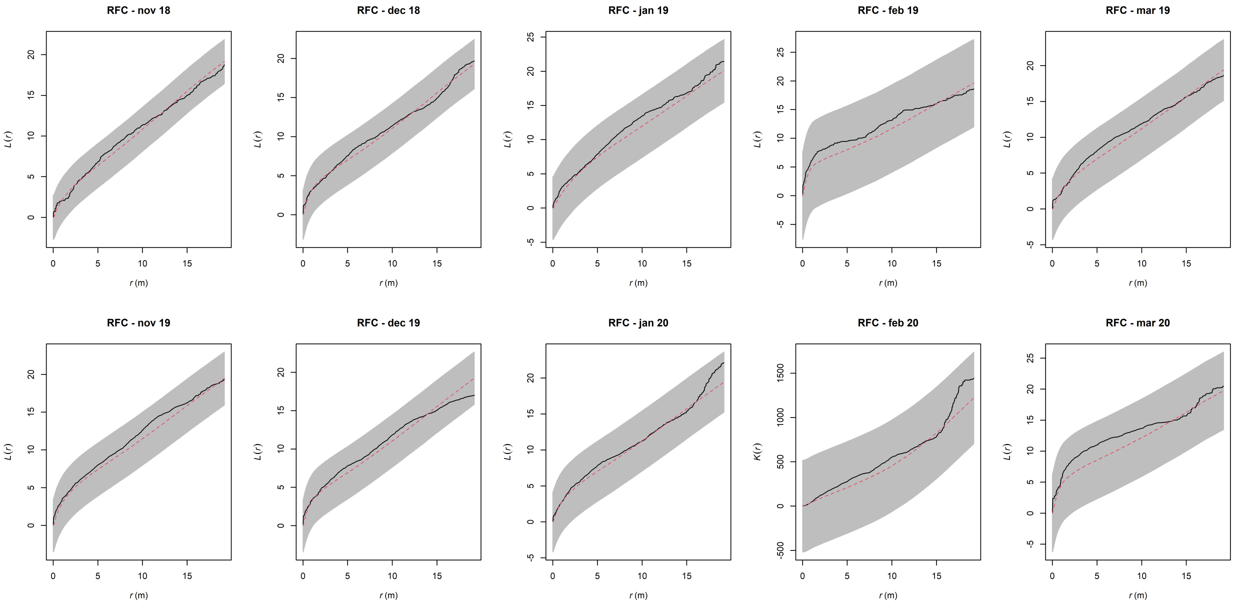


**Supplementary Figure** 17: Null hypothesis testing using global envelopes of the L-function applied to *Rhinoderma darwinii* data from RFC. The observed $L(r)$, represented by the black line, falls within the envelope (grey area) of the predicted $L(r)$ (red dashed line), indicating failure to reject the null hypothesis that the point pattern is governed by a log-Gaussian Cox process.

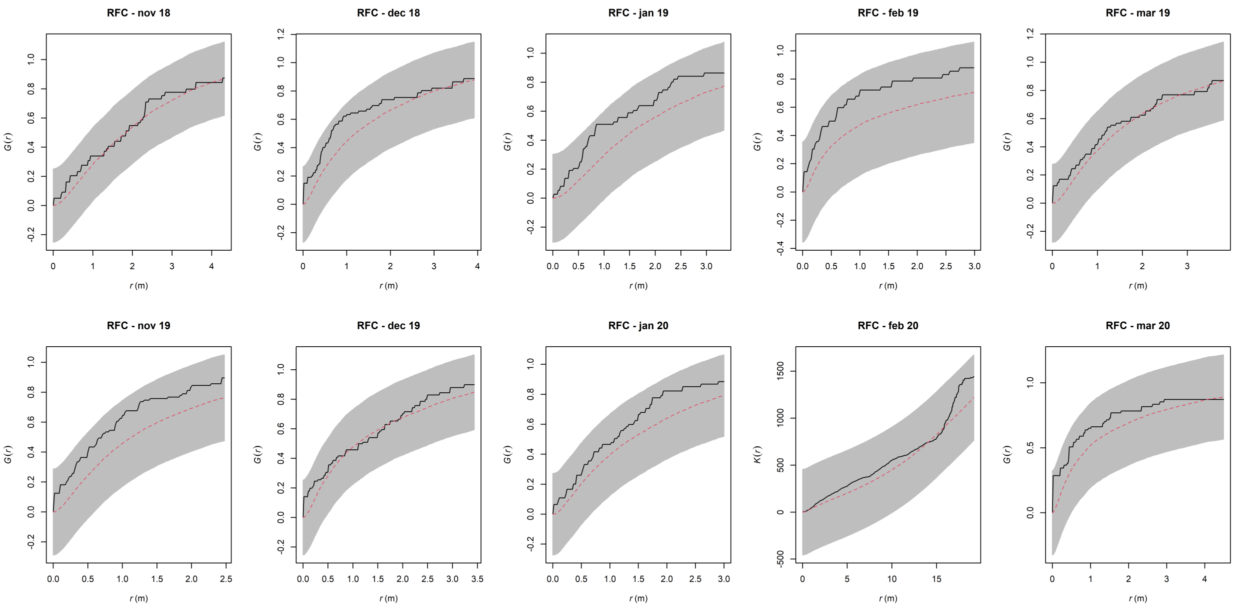


**Supplementary Figure** 18: Null hypothesis testing using global envelopes of the G-function applied to *Rhinoderma darwinii* data from RFC. The observed $L(r)$, represented by the black line, falls within the envelope (grey area) of the predicted $L(r)$ (red dashed line), indicating failure to reject the null hypothesis that the point pattern is governed by a log-Gaussian Cox process.

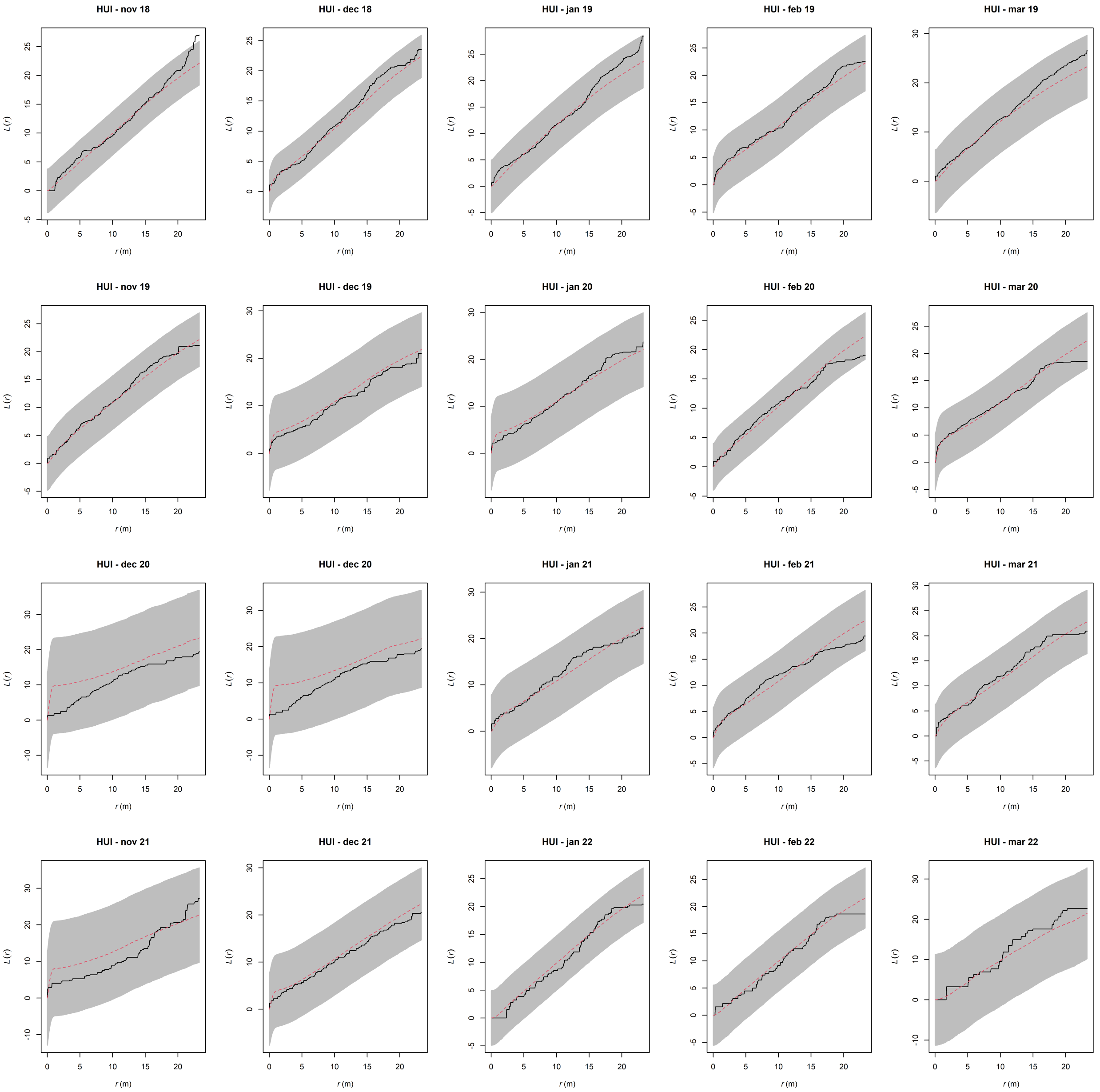


**Supplementary Figure** 19: Null hypothesis testing using global envelopes of the L-function applied to *Rhinoderma darwinii* data from HUI. The observed $L(r)$, represented by the black line, falls within the envelope (grey area) of the predicted $L(r)$ (red dashed line), indicating failure to reject the null hypothesis that the point pattern is governed by a log-Gaussian Cox process.

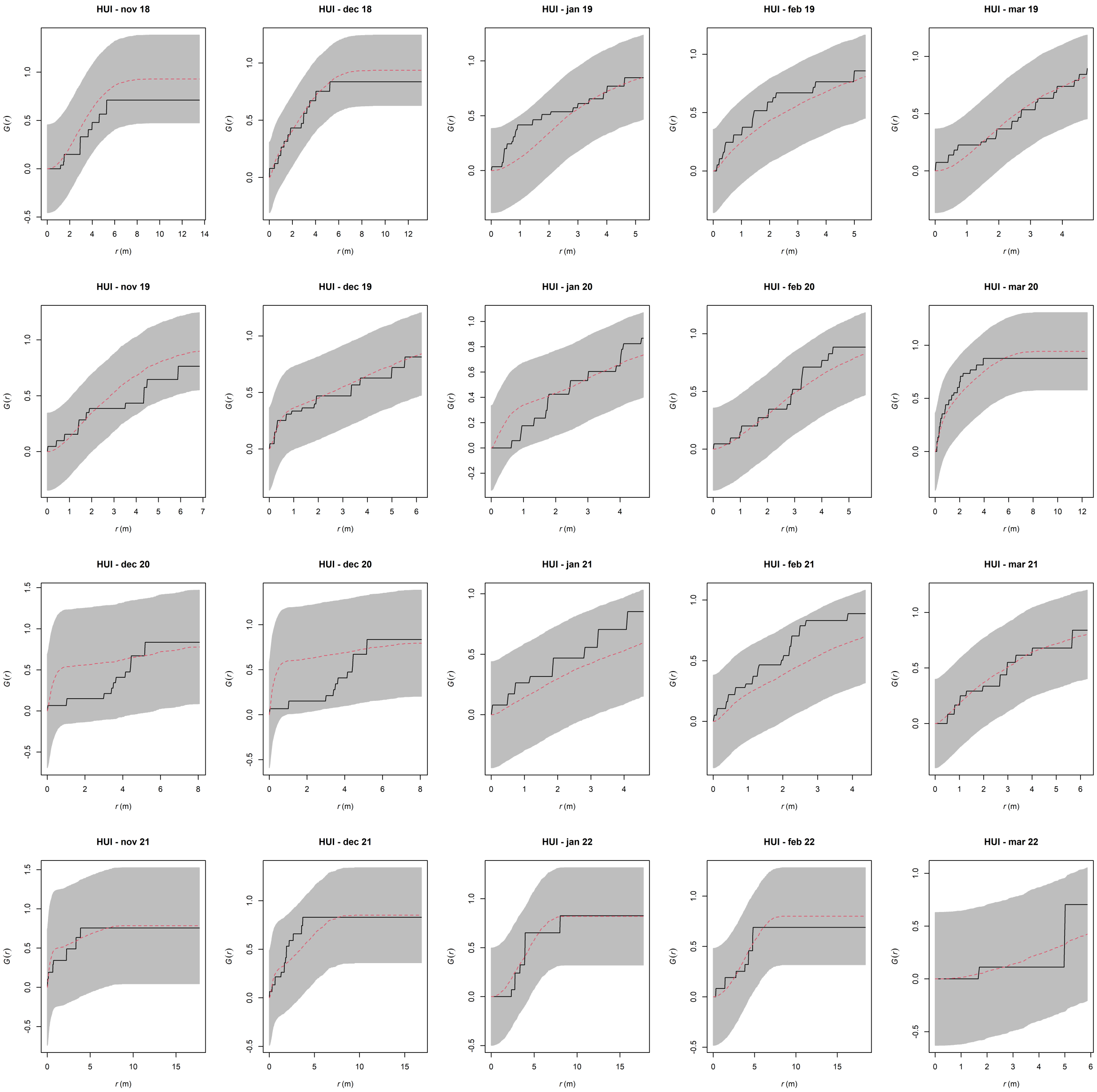


**Supplementary Figure** 20: Null hypothesis testing using global envelopes of the G-function applied to *Rhinoderma darwinii* data from HUI. The observed $L(r)$, represented by the black line, falls within the envelope (grey area) of the predicted $L(r)$ (red dashed line), indicating failure to reject the null hypothesis that the point pattern is governed by a log-Gaussian Cox process.

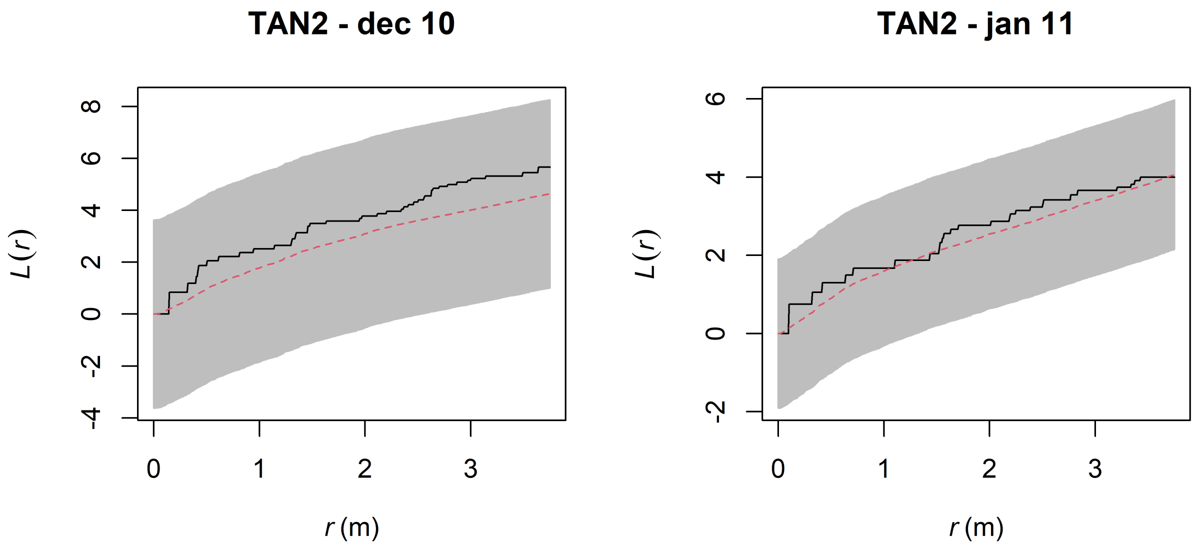


**Supplementary Figure** 21: Null hypothesis testing using global envelopes of the L-function applied to *Rhinoderma darwinii* data from TAN. The observed $L(r)$, represented by the black line, falls within the envelope (grey area) of the predicted $L(r)$ (red dashed line), indicating failure to reject the null hypothesis that the point pattern is governed by a log-Gaussian Cox process.

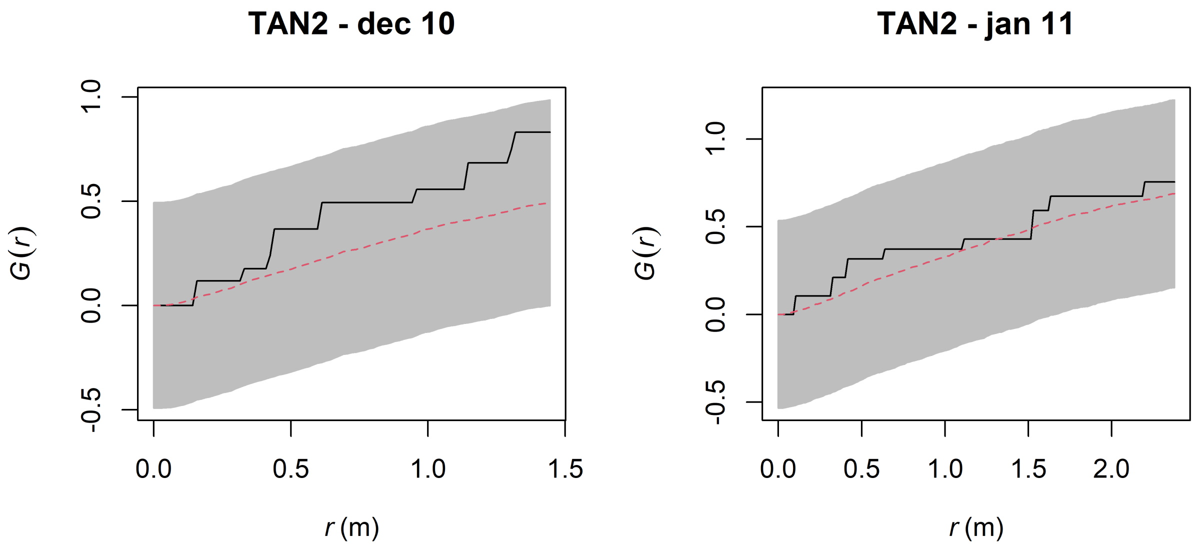


**Supplementary Figure** 22: Null hypothesis testing using global envelopes of the G-function applied to *Rhinoderma darwinii* data from TAN. The observed $L(r)$, represented by the black line, falls within the envelope (grey area) of the predicted $L(r)$ (red dashed line), indicating failure to reject the null hypothesis that the point pattern is governed by a log-Gaussian Cox process.

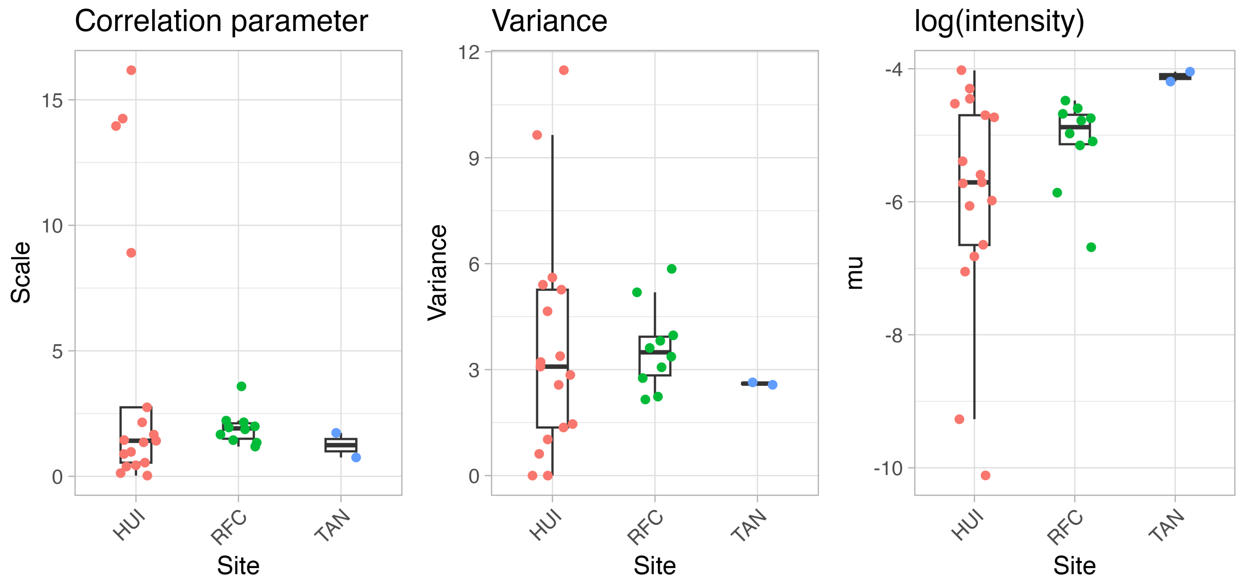


**Supplementary Figure** 23: Estimated parameters of a log-Gaussian Cox process model fitted to point pattern data of *Rhinoderma darwinii* from RFC, HUI, and TAN in southern Chile. Each dot represents data from a single month. Boxplots show the median (horizontal line), interquartile range (box), and whiskers extending to the most extreme values within 1.5× the interquartile range from the box, based on the monthly values.

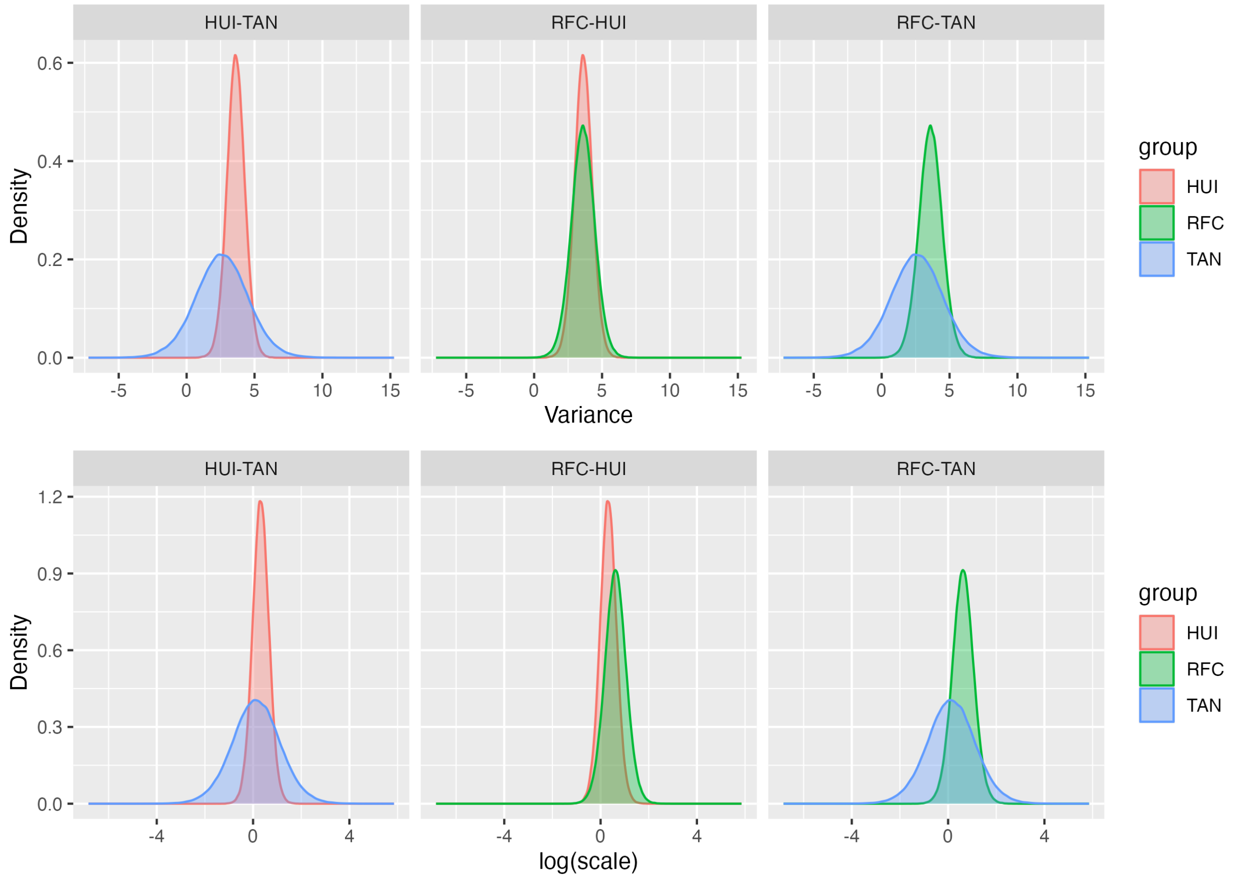


**Supplementary Figure** 24: Posterior distribution of the mean variance and scale (estimated using a Bayesian linear model) of log-Gaussian Cox process models fitted to point pattern data of *Rhinoderma darwinii* from RFC, HUI, and TAN in southern Chile.

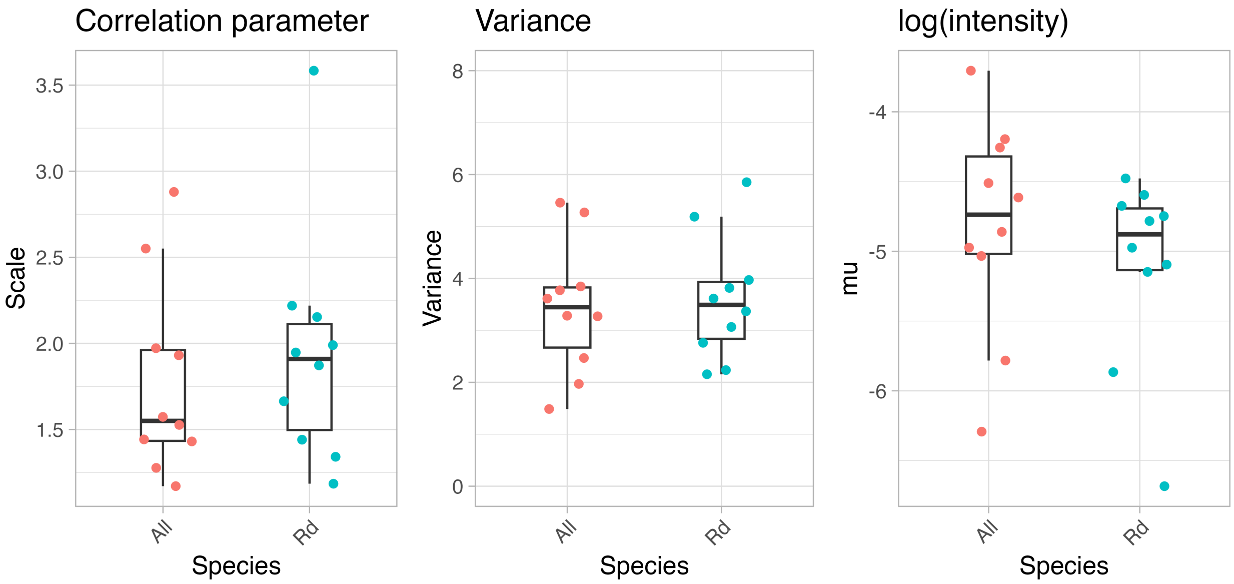


**Supplementary Figure** 25: Estimated parameters of a log-Gaussian Cox process model fitted to point pattern data of *Rhinoderma darwinii*-only and *Rhinoderma darwinii*+*Eupsophus* spp. (all) from RFC. Each dot represents data from a single month. Boxplots show the median (horizontal line), interquartile range (box), and whiskers extending to the most extreme values within 1.5× the interquartile range from the box, based on the monthly values.

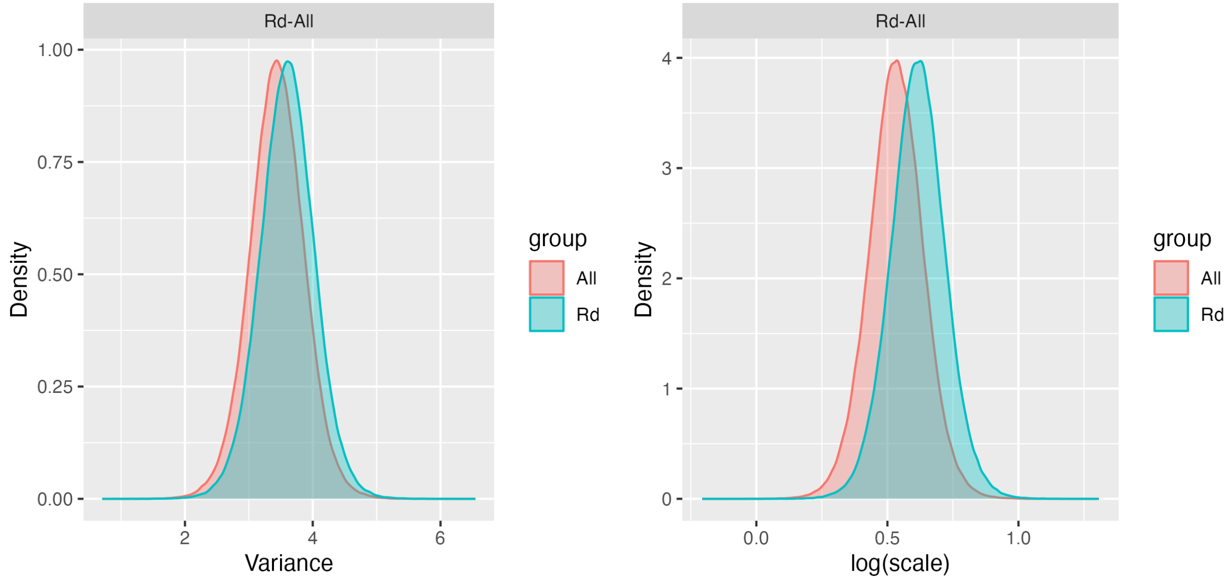


**Supplementary Figure** 26: Posterior distribution of the mean variance and scale (estimated using a Bayesian linear model) of log-Gaussian Cox process models fitted to point pattern data of *Rhinoderma darwinii*-only and *Rhinoderma darwinii*+*Eupsophus* spp. (all) from RFC.

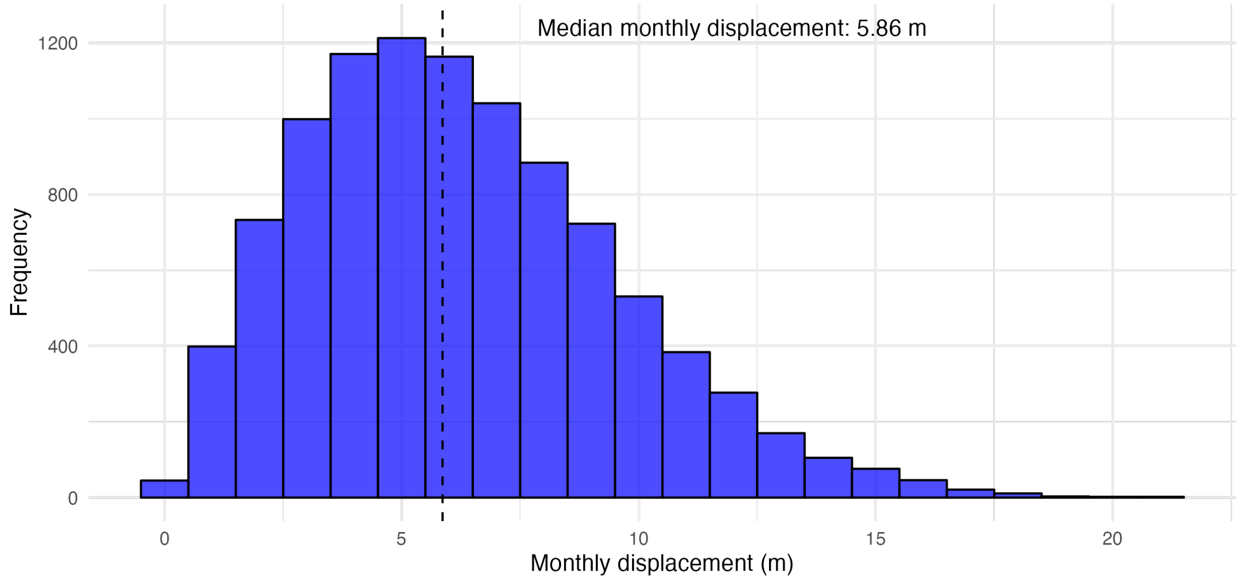


**Supplementary Figure** 27: Monthly displacement distances of 10,000 simulated individuals using $\sigma = 5.000$. The vertical dashed line represents the median.

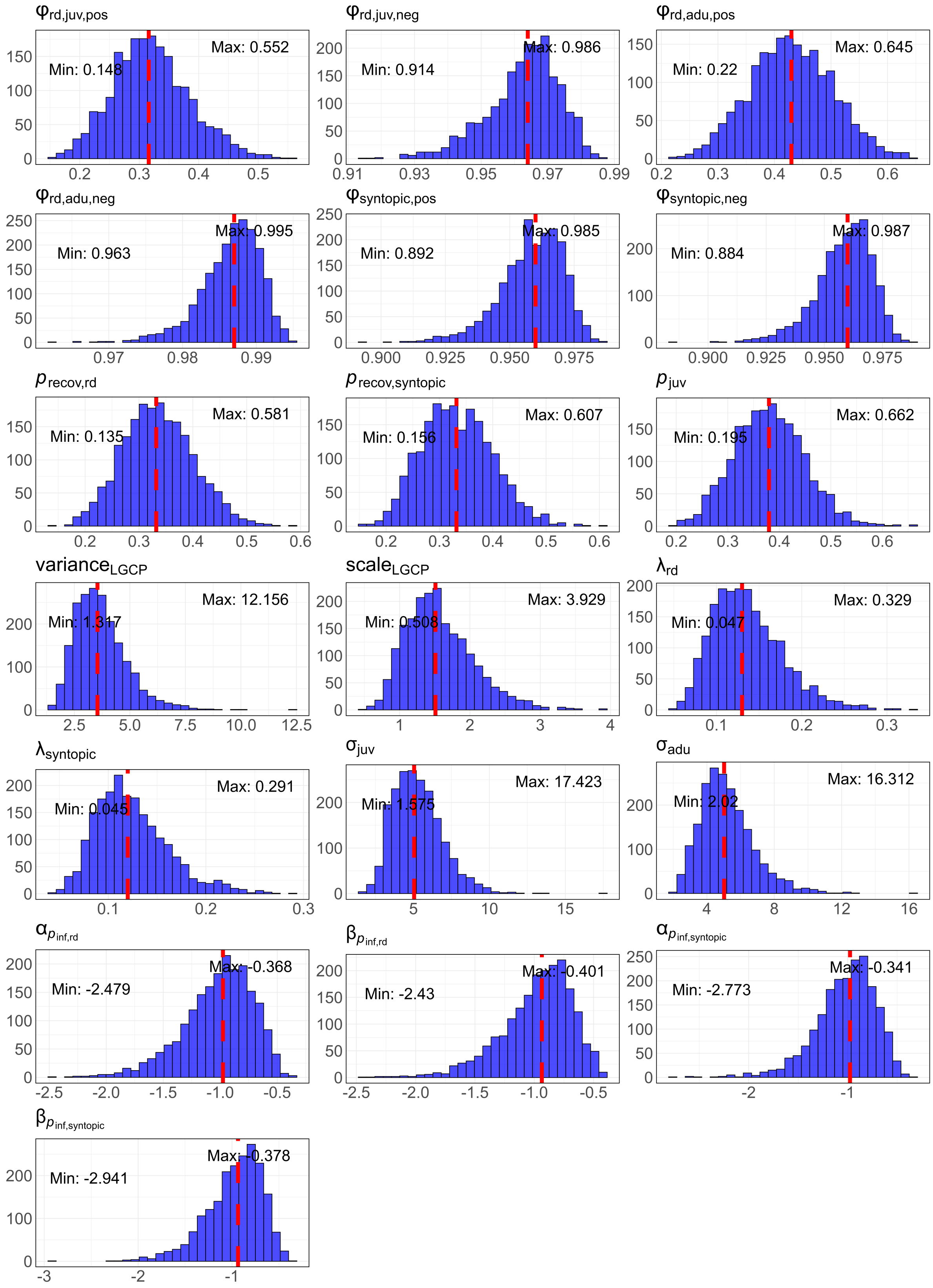


**Supplementary Figure** 28: Frequency distribution of the 19 input parameters of the IBM after perturbation for global sensitivity analysis. The red vertical line indicates the initial parameter value.

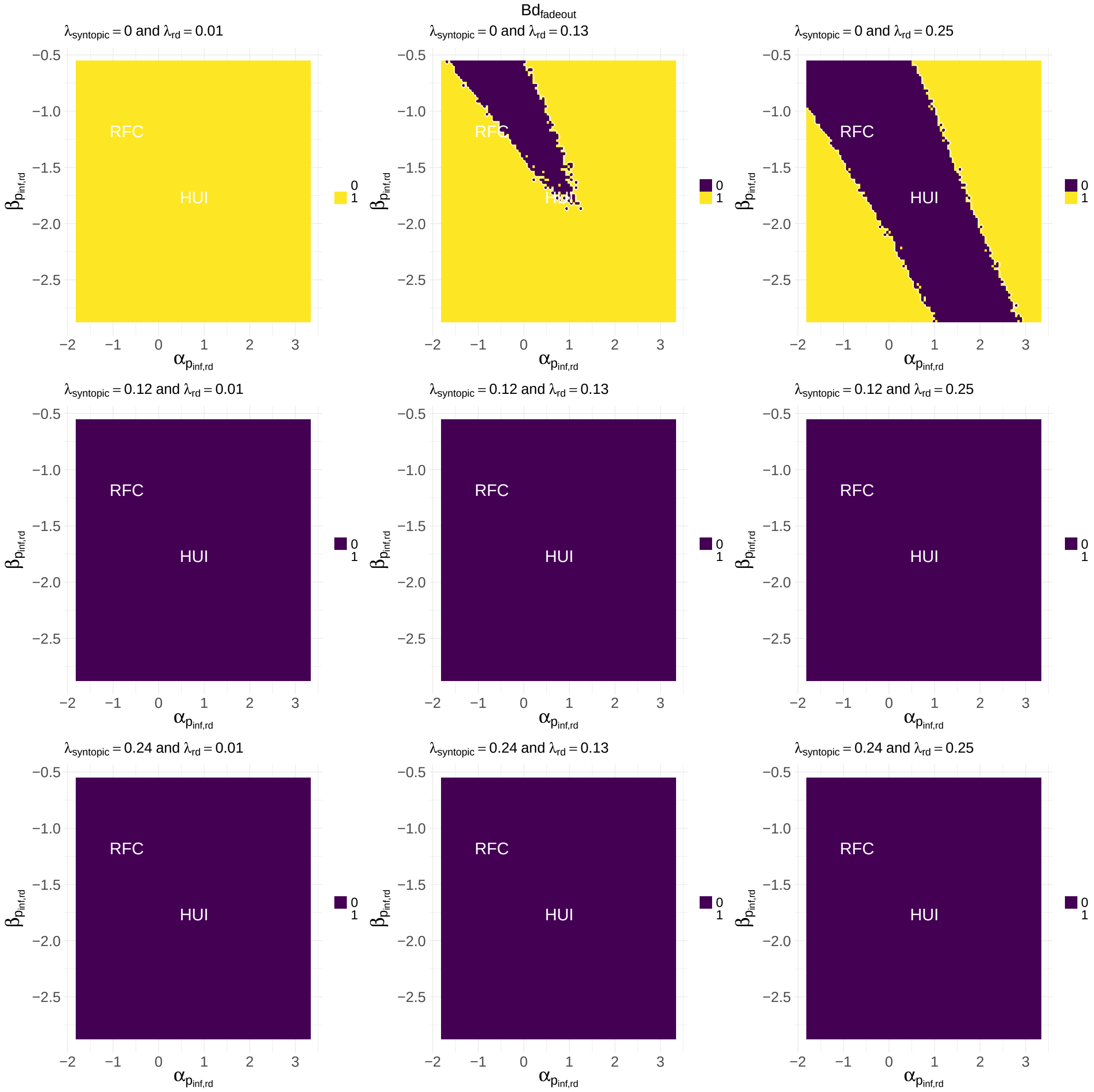


**Supplementary Figure** 29: $Bd_{\text{fadeout}}$ under different combinations of infection parameters and densities of *Rhinoderma darwinii* and a tolerant host species, predicted by the spatial individual-based model. $\alpha_{p_{\text{inf,rd}}}$ represents the intercept and $\beta_{p_{\text{inf,rd}}}$ the regression slope of a logistic regression modelling Bd infection probability as a function of distance to an infected individual. $\lambda_{\text{rd}}$ and $\lambda_{\text{syntopic}}$ denote the density (frogs $m^{-2}$) of *R. darwinii* and the tolerant host, respectively. The results represent the median value from 1,000 simulations for each parameter combination. Contour line indicates integer transition. "RFC" and "HUI" indicate the mean empirical estimates of $\alpha_{p_{\text{inf,rd}}}$ and $\beta_{p_{\text{inf,rd}}}$ in RFC and HUI, respectively.

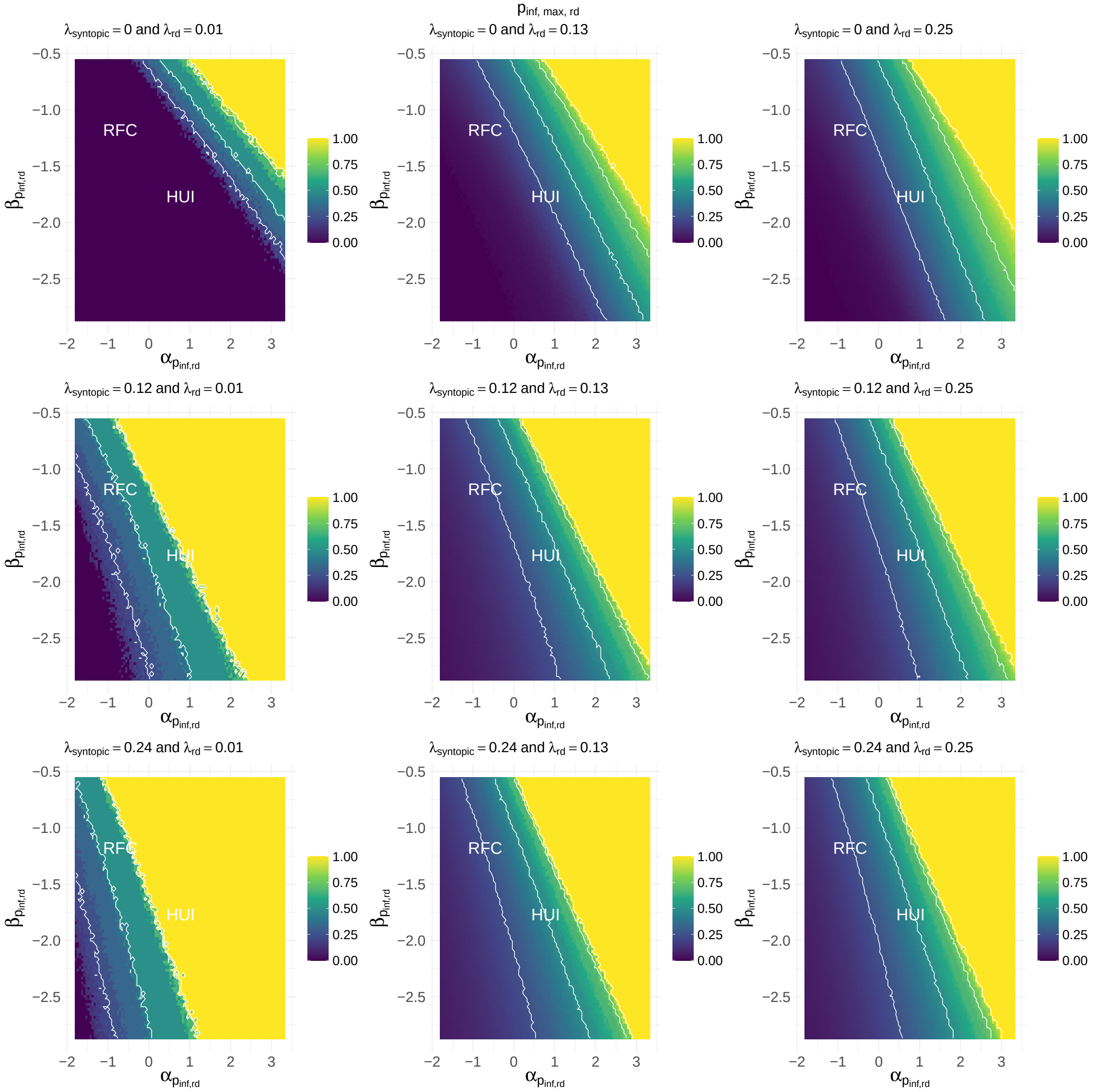


**Supplementary Figure** 30: $p_{inf_{\max,\mathrm{rd}}}$ under different combinations of infection parameters and densities of *Rhinoderma darwinii* and a tolerant host species, predicted by the spatial individual-based model. $\alpha_{p_{\mathrm{inf,rd}}}$ represents the intercept and $\beta_{p_{\mathrm{inf,rd}}}$ the regression slope of a logistic regression modelling Bd infection probability as a function of distance to an infected individual. $\lambda_{\mathrm{rd}}$ and $\lambda_{\mathrm{syntopic}}$ denote the density (frogs $m^{-2}$) of *R. darwinii* and the tolerant host, respectively. The results represent the median value from 1,000 simulations for each parameter combination. Contour lines indicate $p_{inf_{\max,\mathrm{rd}}}$ values of 0.25, 0.5, 0.75 and 1. "RFC" and "HUI" indicate the mean empirical estimates of $\alpha_{p_{\mathrm{inf,rd}}}$ and $\beta_{p_{\mathrm{inf,rd}}}$ in RFC and HUI, respectively.

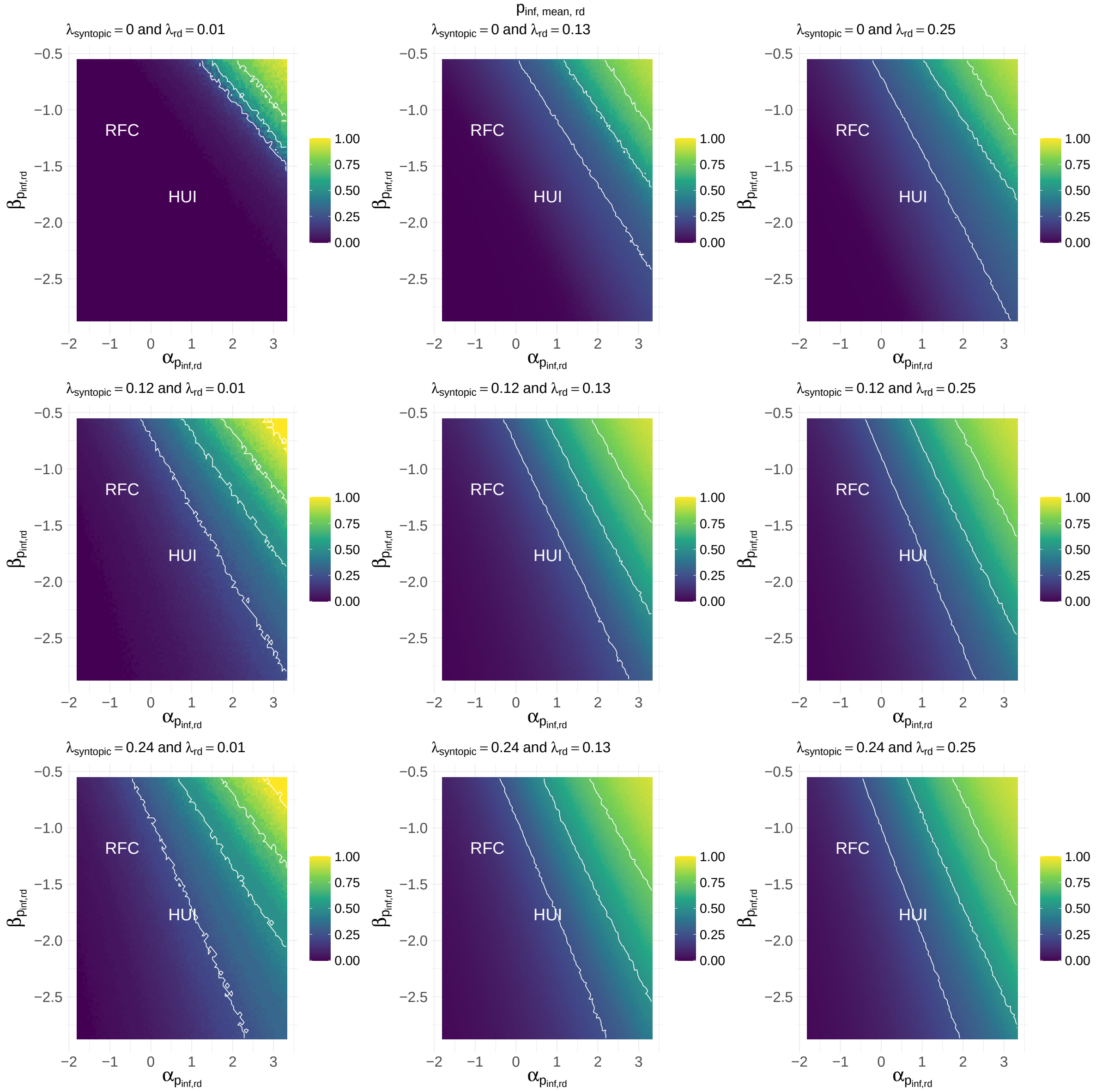


**Supplementary Figure** 31: $p_{inf_{\text{mean,rd}}}$ under different combinations of infection parameters and densities of *Rhinoderma darwinii* and a tolerant host species, predicted by the spatial individual-based model. $\alpha_{p_{\text{inf,rd}}}$ represents the intercept and $\beta_{p_{\text{inf,rd}}}$ the regression slope of a logistic regression modelling Bd infection probability as a function of distance to an infected individual. $\lambda_{\text{rd}}$ and $\lambda_{\text{syntopic}}$ denote the density (frogs $m^{-2}$) of *R. darwinii* and the tolerant host, respectively. The results represent the median value from 1,000 simulations for each parameter combination. Contour lines indicate $p_{inf_{\text{mean,rd}}}$ values of 0.25, 0.5, 0.75 and 1. "RFC" and "HUI" indicate the mean empirical estimates of $\alpha_{p_{\text{inf,rd}}}$ and $\beta_{p_{\text{inf,rd}}}$ in RFC and HUI, respectively.

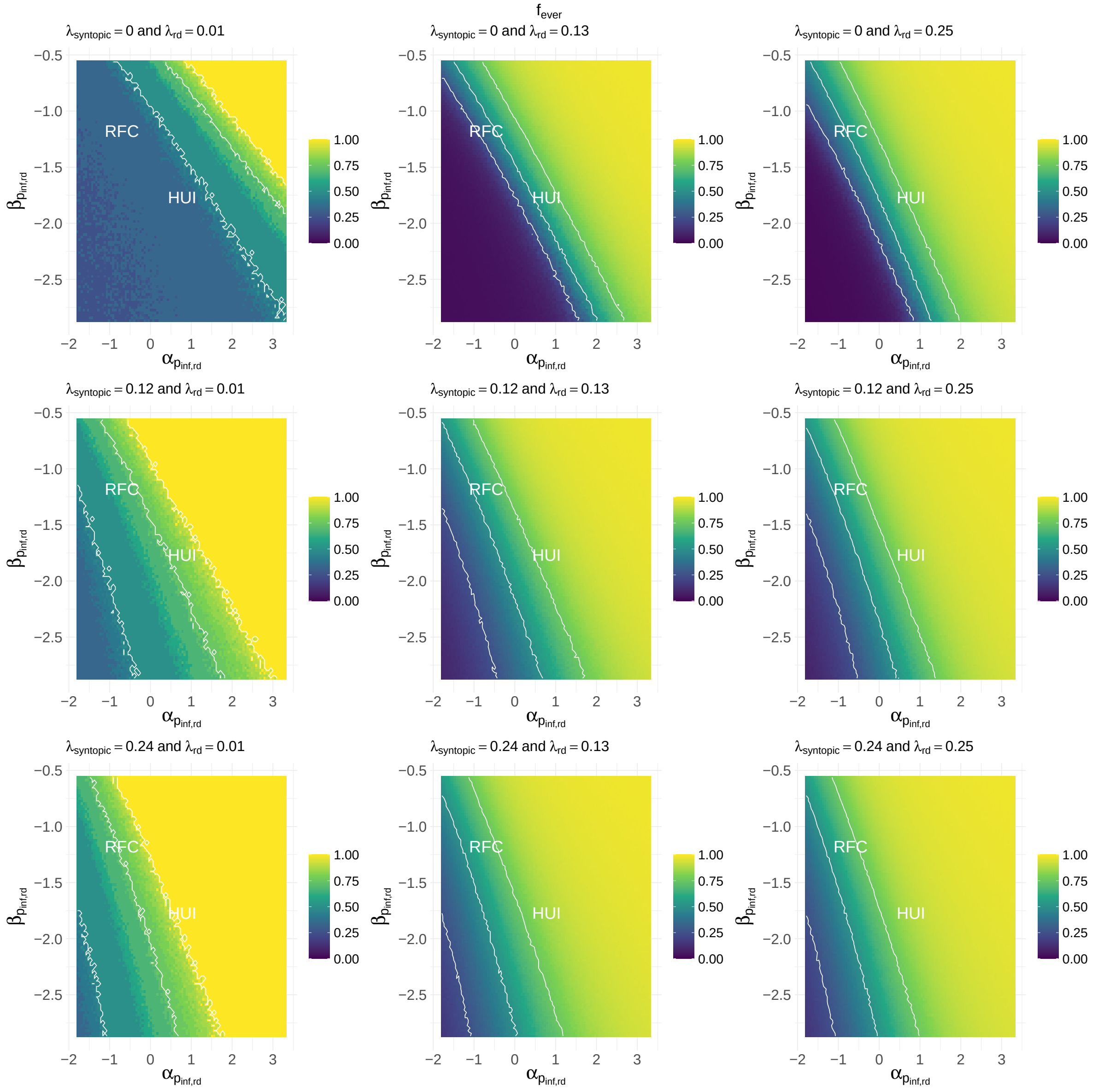


**Supplementary Figure** 32: $f_{\text{ever,inf,rd}}$ under different combinations of infection parameters and densities of *Rhinoderma darwinii* and a tolerant host species, predicted by the spatial individual-based model. $\alpha_{p_{\text{inf,rd}}}$ represents the intercept and $\beta_{p_{\text{inf,rd}}}$ the regression slope of a logistic regression modelling Bd infection probability as a function of distance to an infected individual. $\lambda_{\text{rd}}$ and $\lambda_{\text{syntopic}}$ denote the density (frogs $m^{-2}$) of *R. darwinii* and the tolerant host, respectively. The results represent the median value from 1,000 simulations for each parameter combination. Contour lines indicate $f_{\text{ever,inf,rd}}$ values of 0.25, 0.5, 0.75 and 1. "RFC" and "HUI" indicate the mean empirical estimates of $\alpha_{p_{\text{inf,rd}}}$ and $\beta_{p_{\text{inf,rd}}}$ in RFC and HUI, respectively.

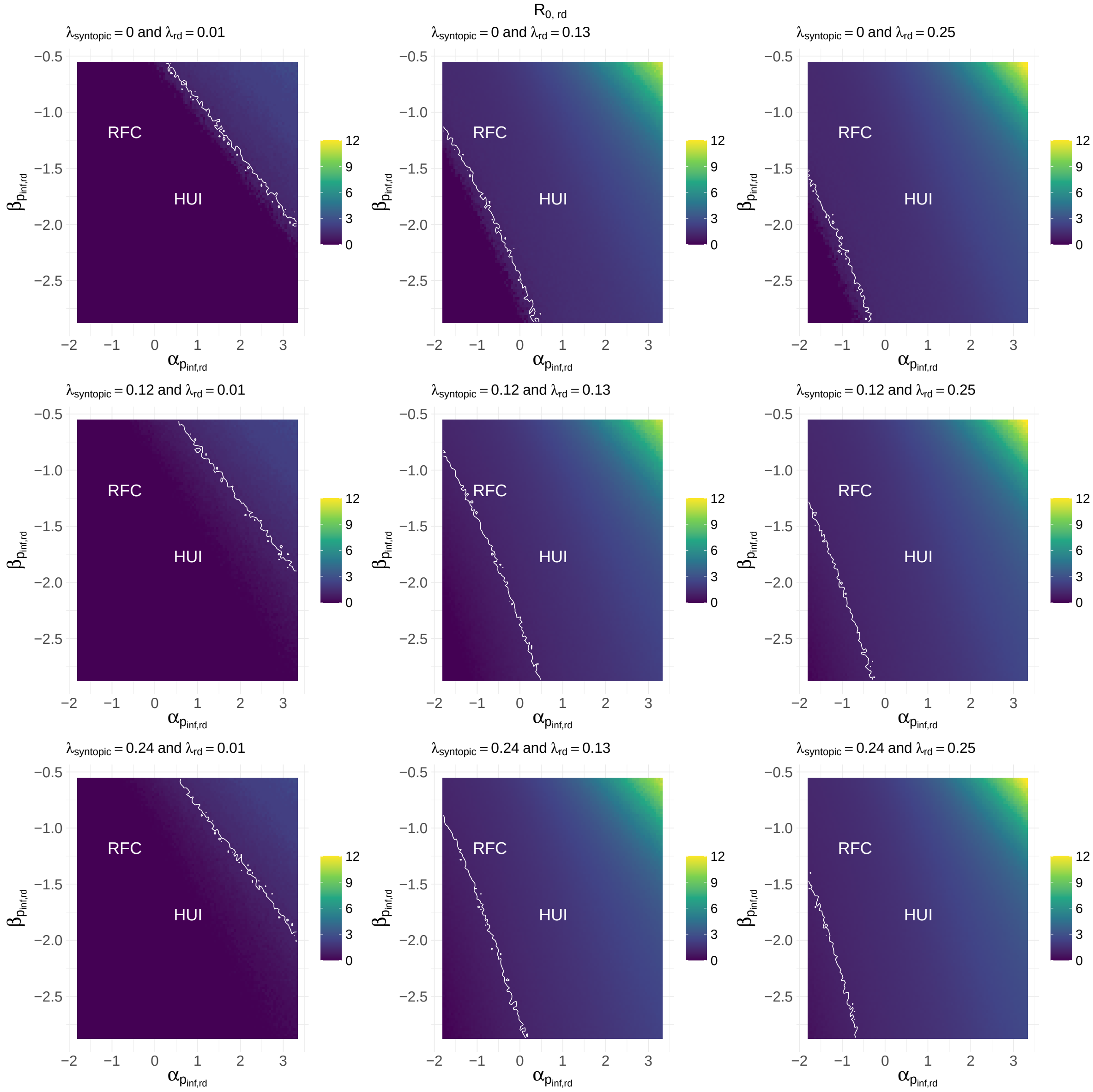


**Supplementary Figure** 33: $R_{0,\mathrm{rd}}$ under different combinations of infection parameters and densities of *Rhinoderma darwinii* and a tolerant host species, predicted by the spatial individual-based model. $\alpha_{p_{\mathrm{inf,rd}}}$ represents the intercept and $\beta_{p_{\mathrm{inf,rd}}}$ the regression slope of a logistic regression modelling Bd infection probability as a function of distance to an infected individual. $\lambda_{\mathrm{rd}}$ and $\lambda_{\mathrm{syntopic}}$ denote the density (frogs $m^{-2}$) of *R. darwinii* and the tolerant host, respectively. The results represent the median value from 1,000 simulations for each parameter combination. Contour line indicates $R_{0,\mathrm{rd}} = 1$. "RFC" and "HUI" indicate the mean empirical estimates of $\alpha_{p_{\mathrm{inf,rd}}}$ and $\beta_{p_{\mathrm{inf,rd}}}$ in RFC and HUI, respectively.

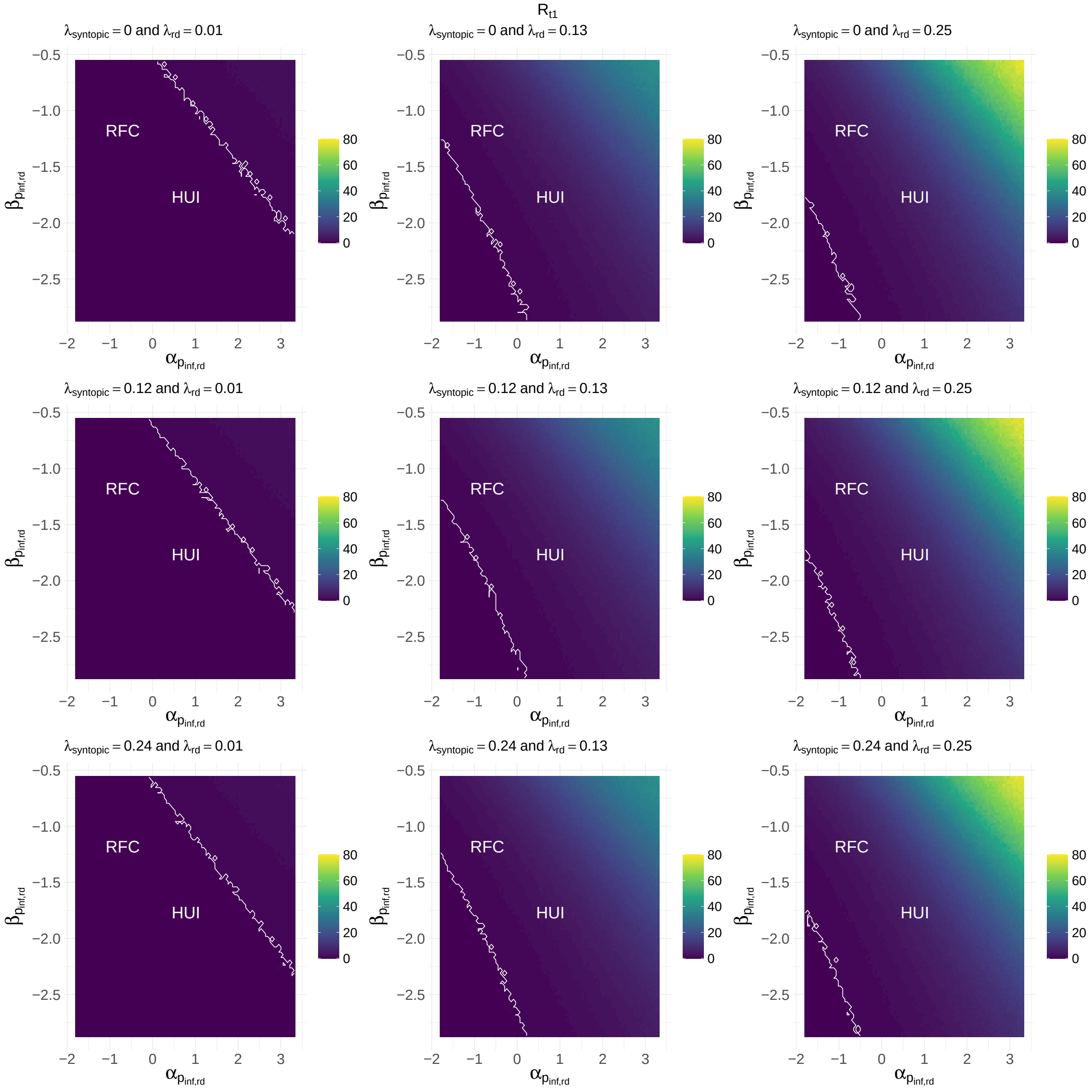


**Supplementary Figure** 34: $R_{\mathrm{t1,rd}}$ under different combinations of infection parameters and densities of *Rhinoderma darwinii* and a tolerant host species, predicted by the spatial individual-based model. $\alpha_{p_{\mathrm{inf,rd}}}$ represents the intercept and $\beta_{p_{\mathrm{inf,rd}}}$ the regression slope of a logistic regression modelling Bd infection probability as a function of distance to an infected individual. $\lambda_{\mathrm{rd}}$ and $\lambda_{\mathrm{syntopic}}$ denote the density (frogs $m^{-2}$) of *R. darwinii* and the tolerant host, respectively. The results represent the median value from 1,000 simulations for each parameter combination. Contour line indicates $R_{\mathrm{t1,rd}} = 1$. "RFC" and "HUI" indicate the mean empirical estimates of $\alpha_{p_{\mathrm{inf,rd}}}$ and $\beta_{p_{\mathrm{inf,rd}}}$ in RFC and HUI, respectively.

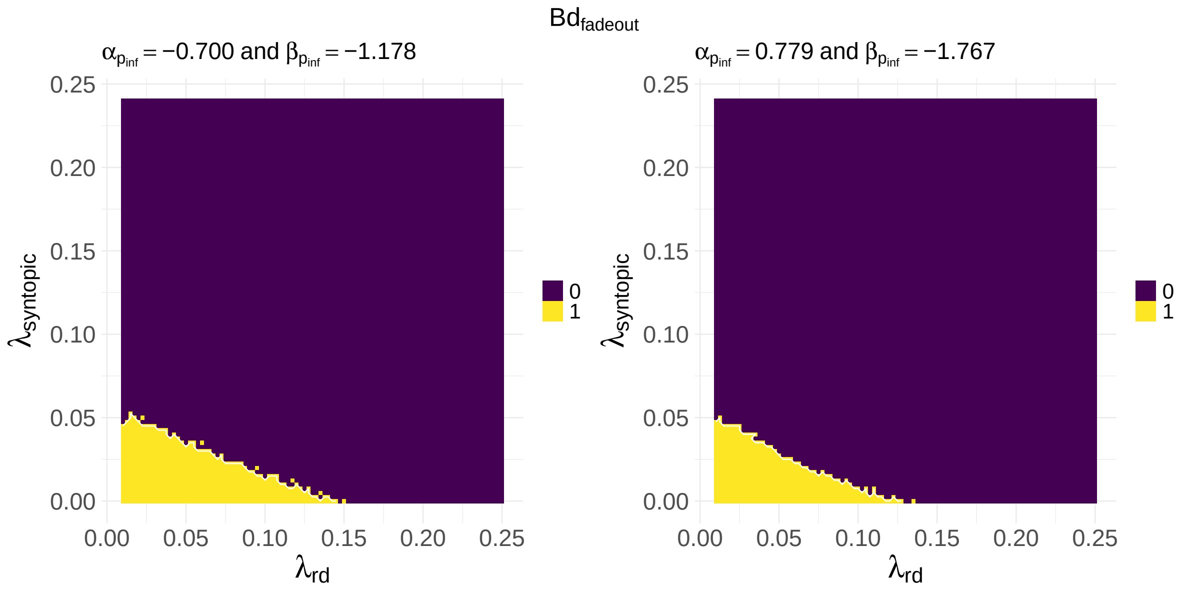


**Supplementary Figure** 35: $Bd_{\text{fadeout}}$ under different combinations of densities of *Rhinoderma darwinii* and a tolerant host species for two combinations of infection parameters ($\alpha_{p_{\text{inf,rd}}}$ and $\beta_{p_{\text{inf,rd}}}$), representing the mean estimates of these parameters from RFC and HUI. $\alpha_{p_{\text{inf,rd}}}$ represents the intercept and $\beta_{p_{\text{inf,rd}}}$ the regression slope of a logistic regression modelling Bd infection probability as a function of distance to an infected individual. $\lambda_{\text{rd}}$ and $\lambda_{\text{syntopic}}$ denote the density (frogs $m^{-2}$) of *R. darwinii* and the tolerant host, respectively. The results represent the median value from 1,000 simulations for each parameter combination. Contour line indicates integer transition.

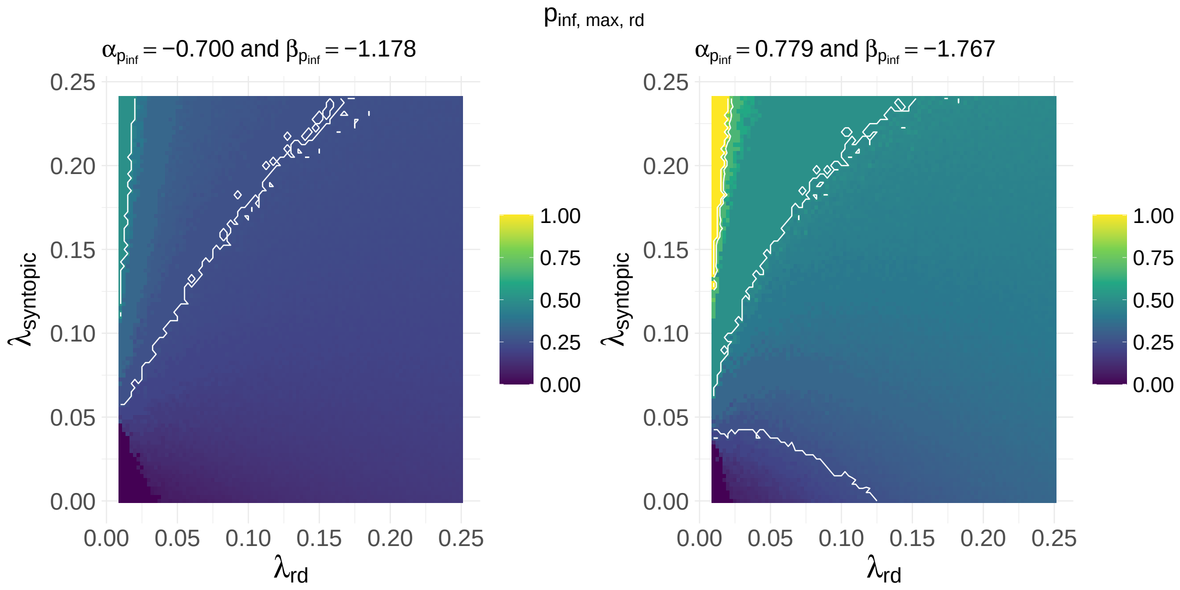


**Supplementary Figure** 36: $p_{inf_{\text{max,rd}}}$ under different combinations of densities of *Rhinoderma darwinii* and a tolerant host species for two combinations of infection parameters ($\alpha_{p_{\text{inf,rd}}}$ and $\beta_{p_{\text{inf,rd}}}$), representing the mean estimates of these parameters from RFC and HUI. $\alpha_{p_{\text{inf,rd}}}$ represents the intercept and $\beta_{p_{\text{inf,rd}}}$ the regression slope of a logistic regression modelling Bd infection probability as a function of distance to an infected individual. $\lambda_{\text{rd}}$ and $\lambda_{\text{syntopic}}$ denote the density (frogs $m^{-2}$) of *R. darwinii* and the tolerant host, respectively. The results represent the median value from 1,000 simulations for each parameter combination. Contour lines indicate $p_{inf_{\text{max,rd}}}$ values of 0.25, 0.5, 0.75 and 1.

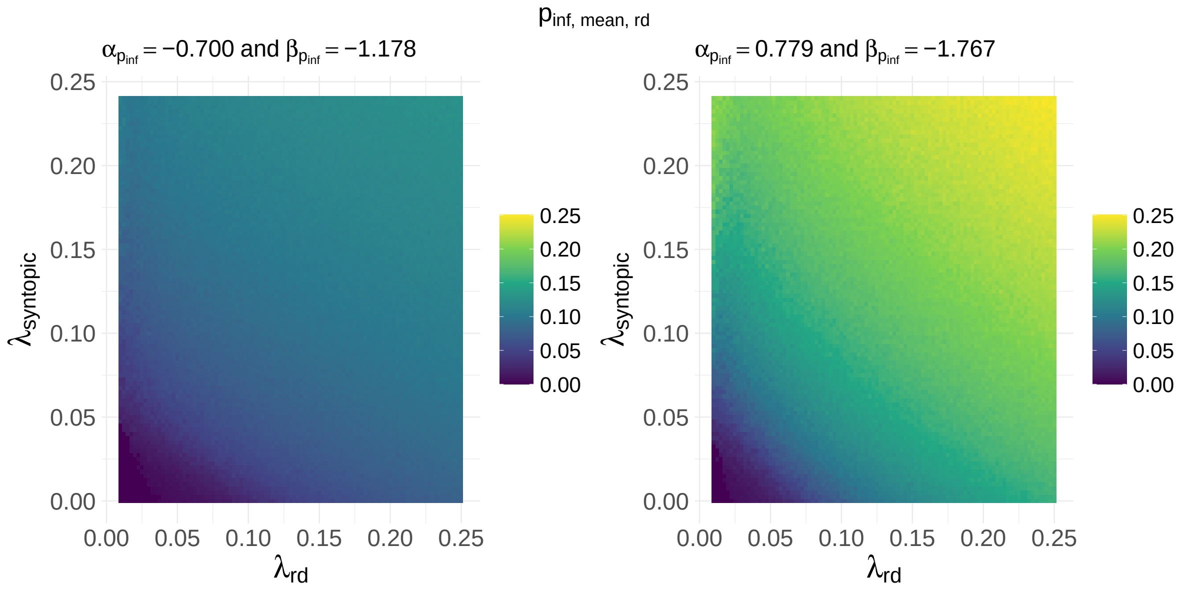


**Supplementary Figure** 37: $p_{inf_{\mathrm{mean,rd}}}$ under different combinations of densities of *Rhinoderma darwinii* and a tolerant host species for two combinations of infection parameters ($\alpha_{p_{\mathrm{inf,rd}}}$ and $\beta_{p_{\mathrm{inf,rd}}}$), representing the mean estimates of these parameters from RFC and HUI. $\alpha_{p_{\mathrm{inf,rd}}}$ represents the intercept and $\beta_{p_{\mathrm{inf,rd}}}$ the regression slope of a logistic regression modelling Bd infection probability as a function of distance to an infected individual. $\lambda_{\mathrm{rd}}$ and $\lambda_{\mathrm{syntopic}}$ denote the density (frogs $m^{-2}$) of *R. darwinii* and the tolerant host, respectively. The results represent the median value from 1,000 simulations for each parameter combination. Contour lines indicate $p_{inf_{\mathrm{mean,rd}}}$ values of 0.25, 0.5, 0.75 and 1.

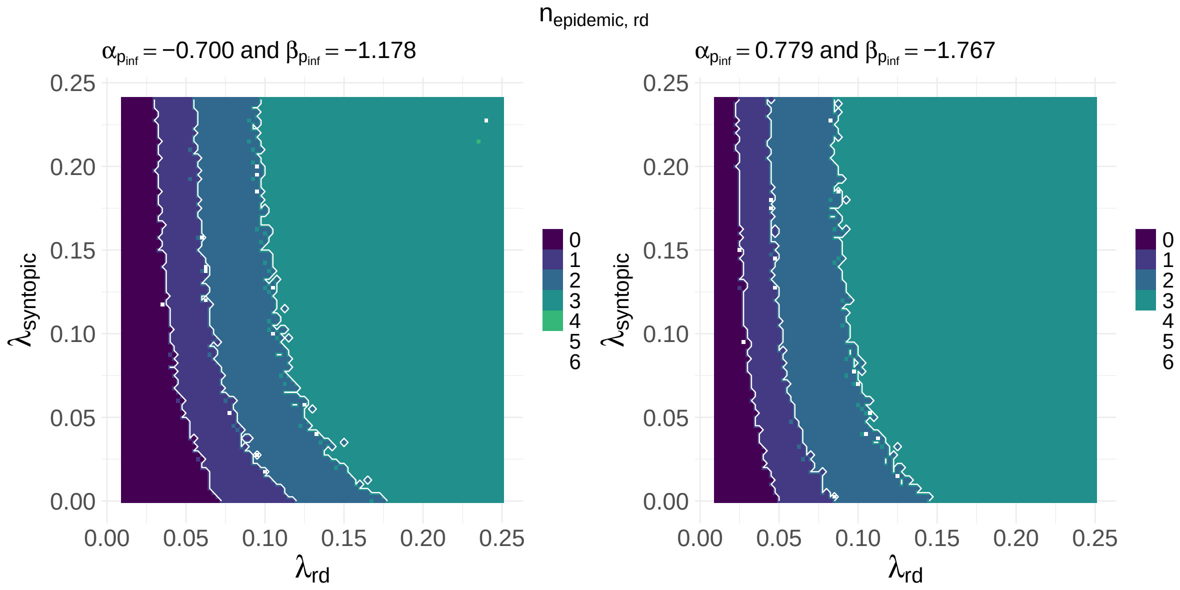


**Supplementary Figure** 38: $n_{\text{epidemic, rd}}$ under different combinations of densities of *Rhinoderma darwinii* and a tolerant host species for two combinations of infection parameters ($\alpha_{p_{\text{inf,rd}}}$ and $\beta_{p_{\text{inf,rd}}}$), representing the mean estimates of these parameters from RFC and HUI. $\alpha_{p_{\text{inf,rd}}}$ represents the intercept and $\beta_{p_{\text{inf,rd}}}$ the regression slope of a logistic regression modelling Bd infection probability as a function of distance to an infected individual. $\lambda_{\text{rd}}$ and $\lambda_{\text{syntopic}}$ denote the density (frogs $m^{-2}$) of *R. darwinii* and the tolerant host, respectively. The results represent the median value from 1,000 simulations for each parameter combination. Contour lines indicate integer transitions.

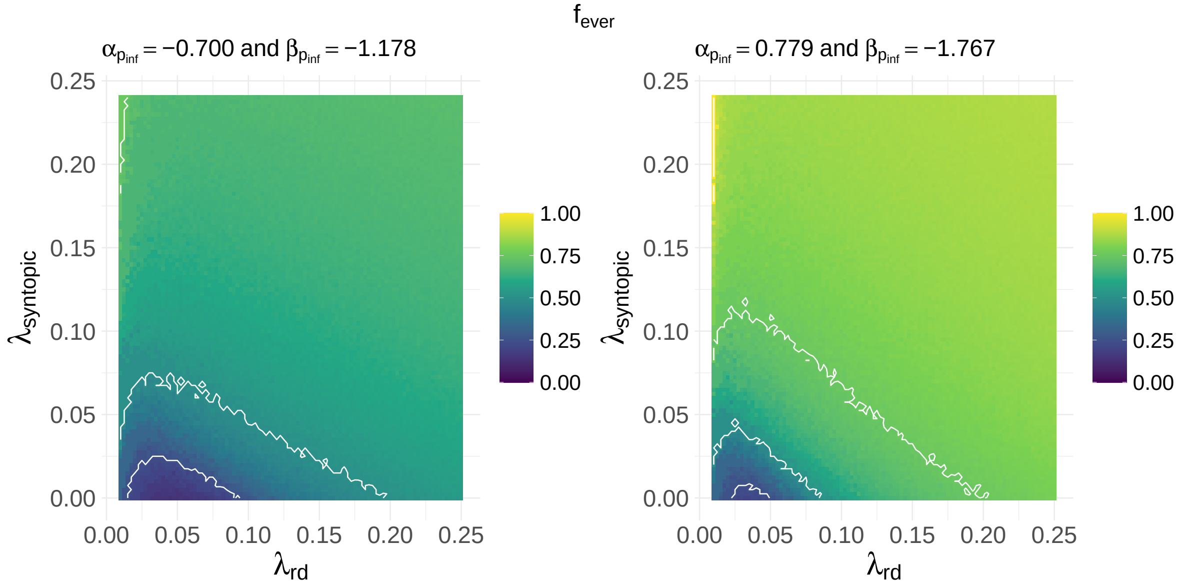


**Supplementary Figure** 39: $f_{\text{ever,inf,rd}}$ under different combinations of densities of *Rhinoderma darwinii* and a tolerant host species for two combinations of infection parameters ($\alpha_{p_{\text{inf,rd}}}$ and $\beta_{p_{\text{inf,rd}}}$), representing the mean estimates of these parameters from RFC and HUI. $\alpha_{p_{\text{inf,rd}}}$ represents the intercept and $\beta_{p_{\text{inf,rd}}}$ the regression slope of a logistic regression modelling Bd infection probability as a function of distance to an infected individual. $\lambda_{\text{rd}}$ and $\lambda_{\text{syntopic}}$ denote the density (frogs $m^{-2}$) of *R. darwinii* and the tolerant host, respectively. The results represent the median value from 1,000 simulations for each parameter combination. Contour lines indicate $f_{\text{ever,inf,rd}}$ values of 0.25, 0.5, 0.75 and 1.

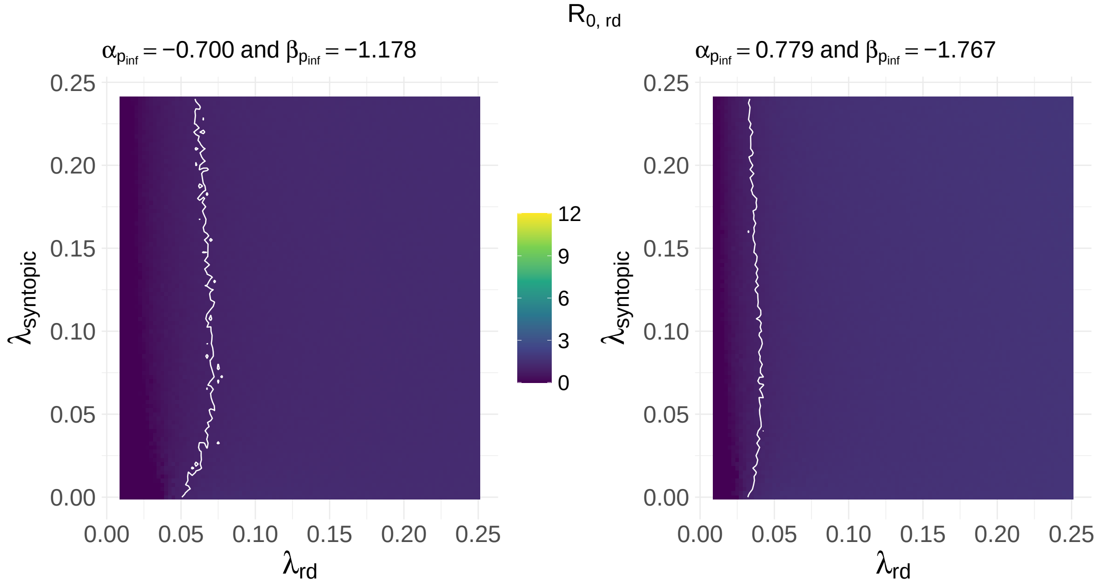


**Supplementary Figure** 40: $R_{0,\mathrm{rd}}$ under different combinations of densities of *Rhinoderma darwinii* and a tolerant host species for two combinations of infection parameters ($\alpha_{p_{\mathrm{inf,rd}}}$ and $\beta_{p_{\mathrm{inf,rd}}}$), representing the mean estimates of these parameters from RFC and HUI. $\alpha_{p_{\mathrm{inf,rd}}}$ represents the intercept and $\beta_{p_{\mathrm{inf,rd}}}$ the regression slope of a logistic regression modelling Bd infection probability as a function of distance to an infected individual. $\lambda_{\mathrm{rd}}$ and $\lambda_{\mathrm{syntopic}}$ denote the density (frogs $m^{-2}$) of *R. darwinii* and the tolerant host, respectively. The results represent the median value from 1,000 simulations for each parameter combination. Contour line indicates $R_{0,\mathrm{rd}} = 1$.

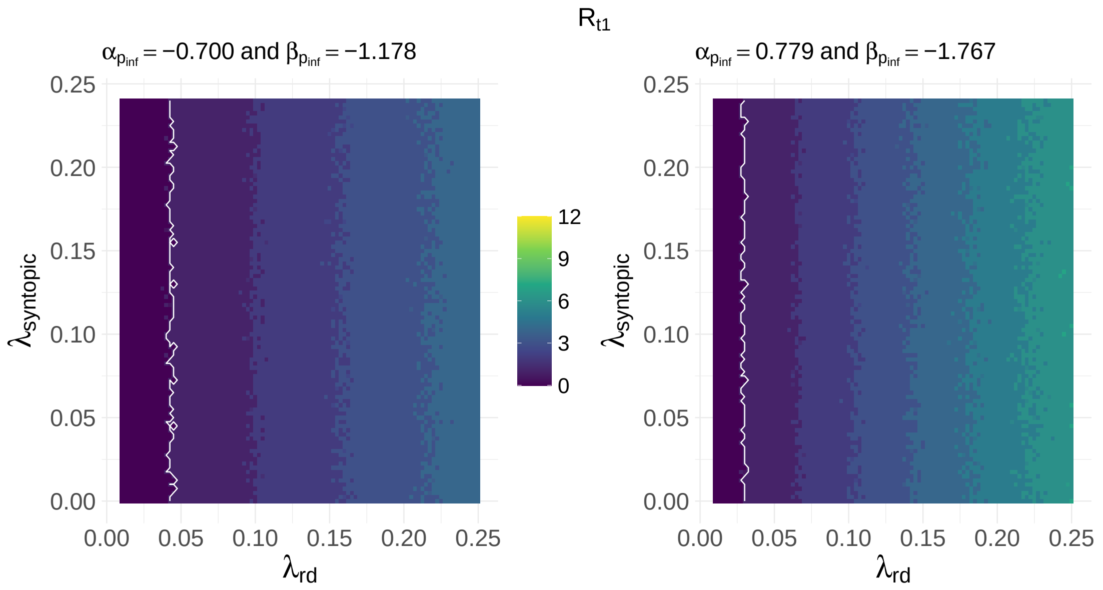


**Supplementary Figure** 41: $R_{\mathrm{t1,rd}}$ under different combinations of densities of *Rhinoderma darwinii* and a tolerant host species for two combinations of infection parameters ($\alpha_{p_{\mathrm{inf,rd}}}$ and $\beta_{p_{\mathrm{inf,rd}}}$), representing the mean estimates of these parameters from RFC and HUI. $\alpha_{p_{\mathrm{inf,rd}}}$ represents the intercept and $\beta_{p_{\mathrm{inf,rd}}}$ the regression slope of a logistic regression modelling Bd infection probability as a function of distance to an infected individual. $\lambda_{\mathrm{rd}}$ and $\lambda_{\mathrm{syntopic}}$ denote the density (frogs $m^{-2}$) of *R. darwinii* and the tolerant host, respectively. The results represent the median value from 1,000 simulations for each parameter combination. Contour line indicates $R_{\mathrm{t1,rd}} = 1$.

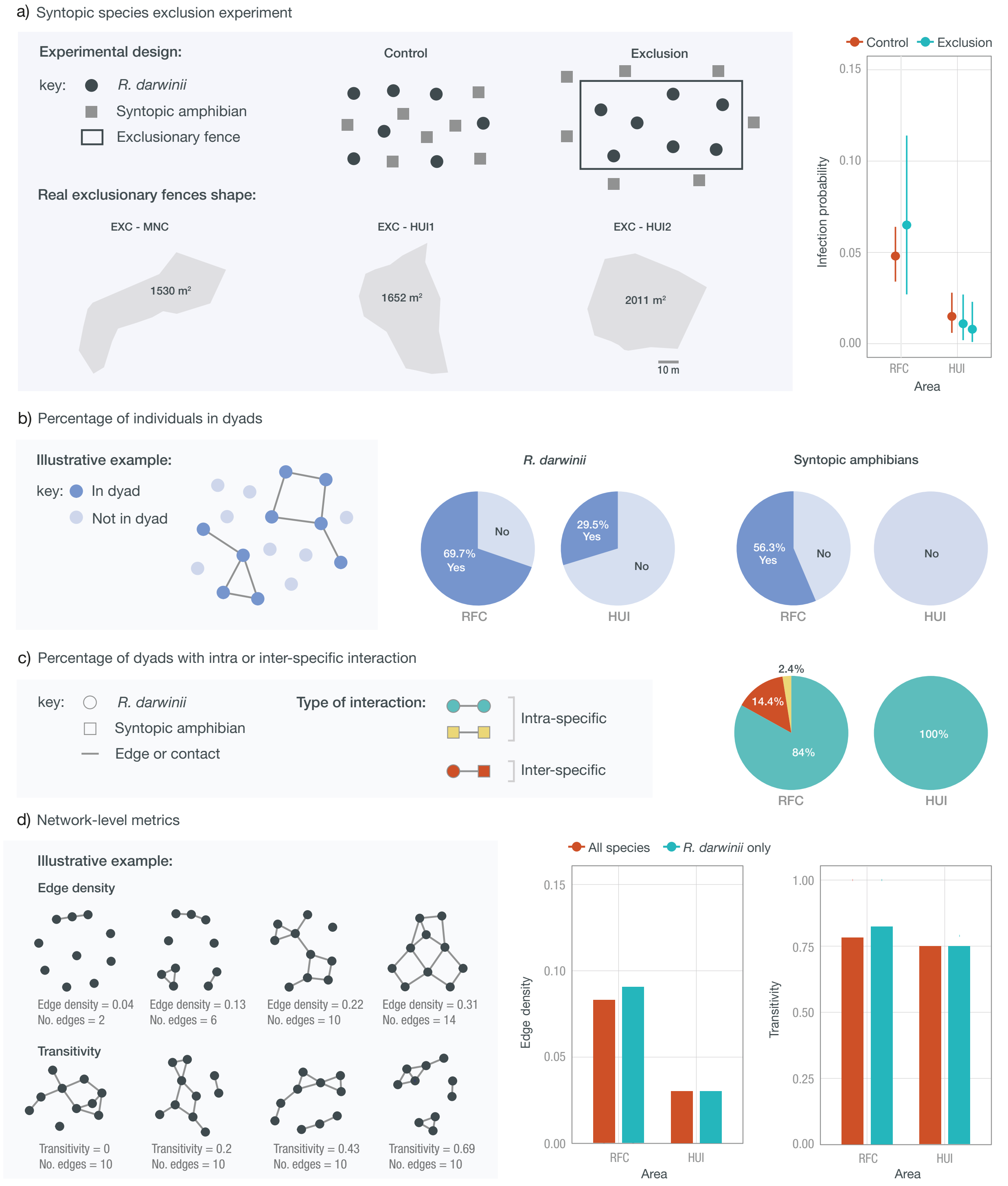


**Supplementary Figure** 42: Role of syntopic amphibians in Bd infection dynamics in *R. darwinii* populations. (a) Syntopic amphibians exclusion experiment and its impact on Bd infection probability in *R. darwinii*. (b) Median percentage of *R. darwinii* and syntopic amphibians that were observed in an active dyad during each primary capture occasion. (c) Median percentage of active dyads that corresponded to intra- or inter-specific interactions. (d) Comparison of network-level metrics from a social network analysis on a network constructed considering either all amphibian species or *R. darwinii* individuals only.

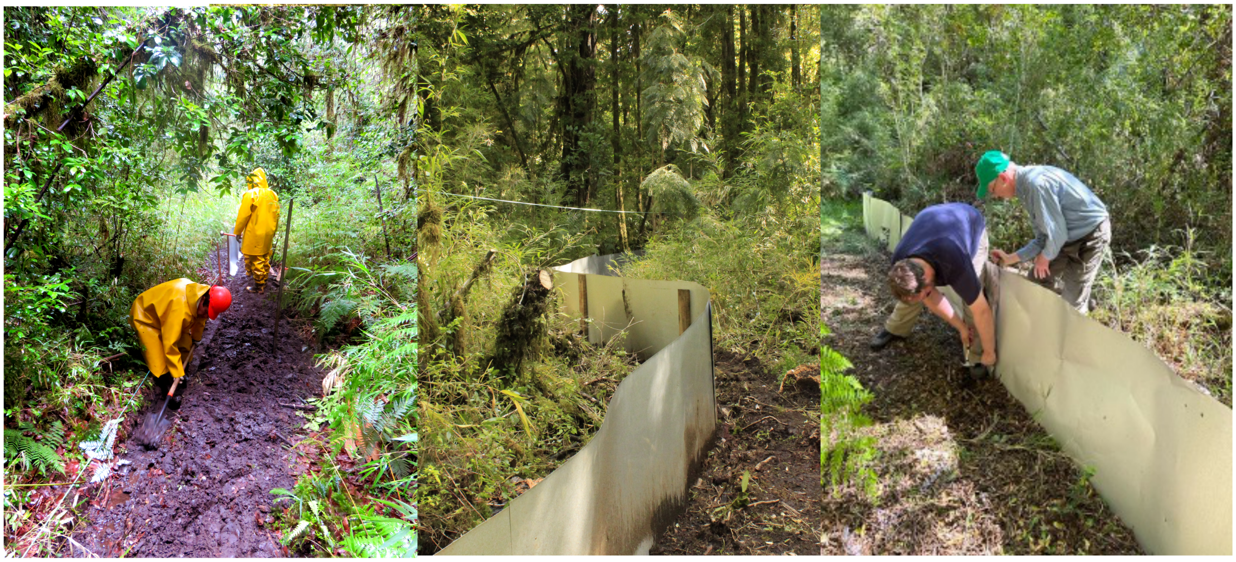

**Supplementary Figure** 43: Installation of the syntopic amphibian exclusion experiment.

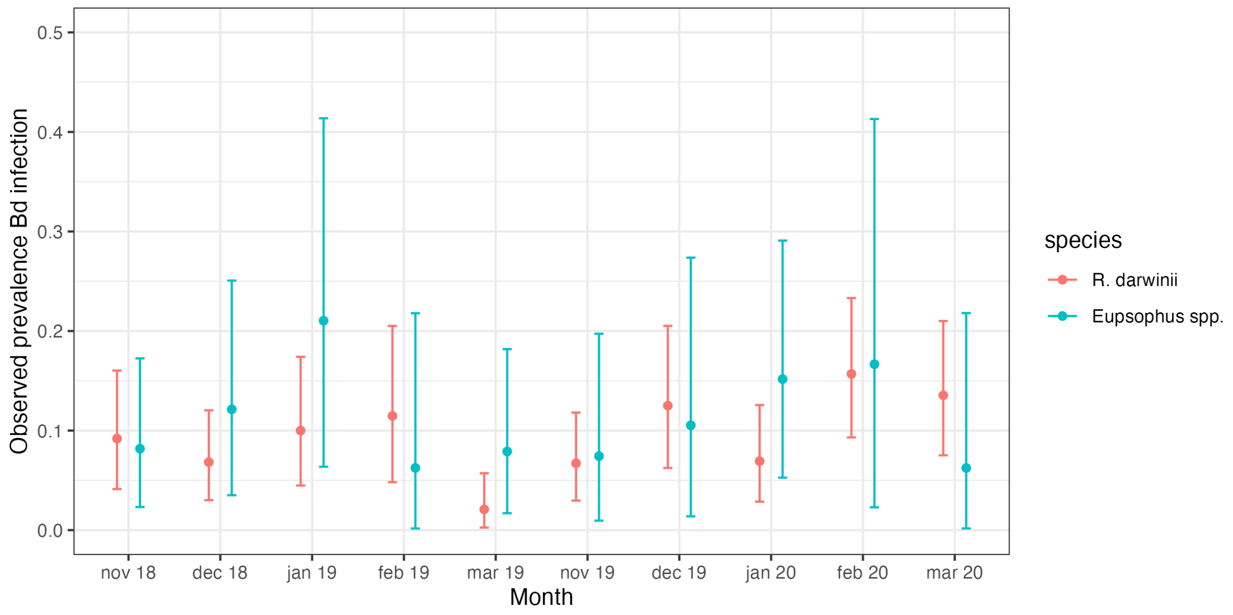


**Supplementary Figure** 44: Estimated prevalence of *Batrachochytrium dendrobatidis* infection in *Rhinoderma darwinii* and *Eupsophus* spp. from RFC. Points represent the posterior means estimated using a Bayesian binomial model, and error bars indicate 95% Bayesian credible intervals. The model was fitted to data from 419 *R. darwinii* and 216 *Eupsophus* spp. individuals.

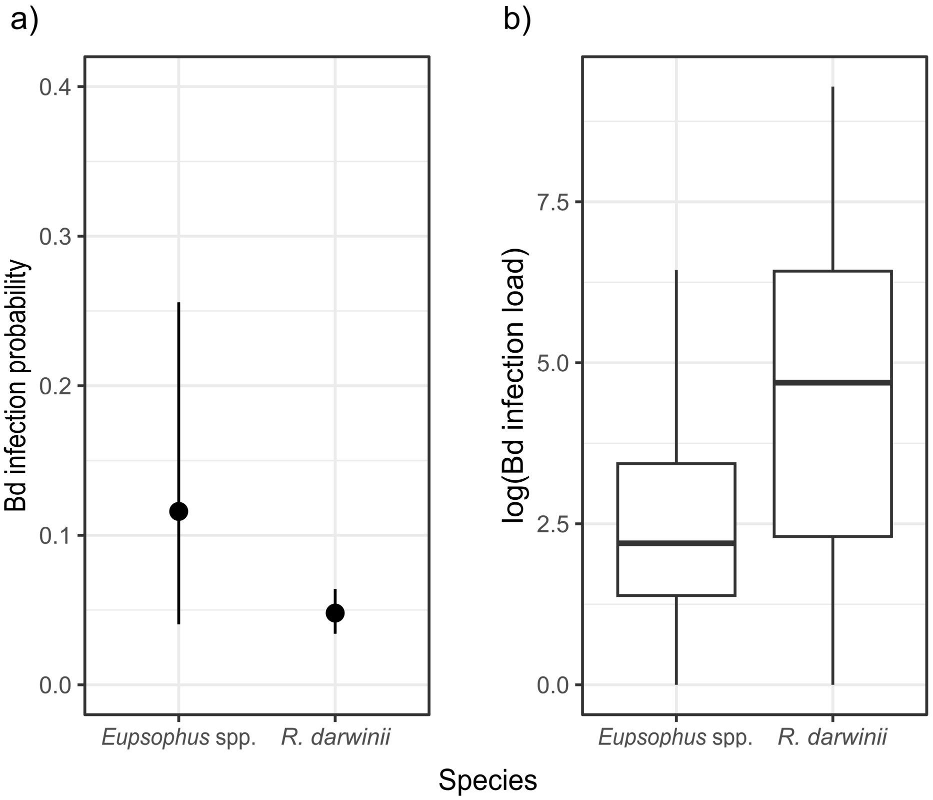


**Supplementary Figure** 45: Average *Batrachochytrium dendrobatidis* infection probability (a) and infection load (b) in *Rhinoderma darwinii* and *Eupsophus* spp. from RFC. In (a), points represent the posterior means estimated using a Bayesian multistate capture-recapture model fitted to data from 419 *R. darwinii* and 216 *Eupsophus* spp. individuals, and error bars indicate 95% Bayesian credible intervals. In (b), boxplots show the median (horizontal line), interquartile range (box), and whiskers extending to the most extreme values within 1.5× the interquartile range from the box, based on infection loads of Bd-positive samples (*R. darwinii*, n = 76; *Eupsophus* spp., n = 21).

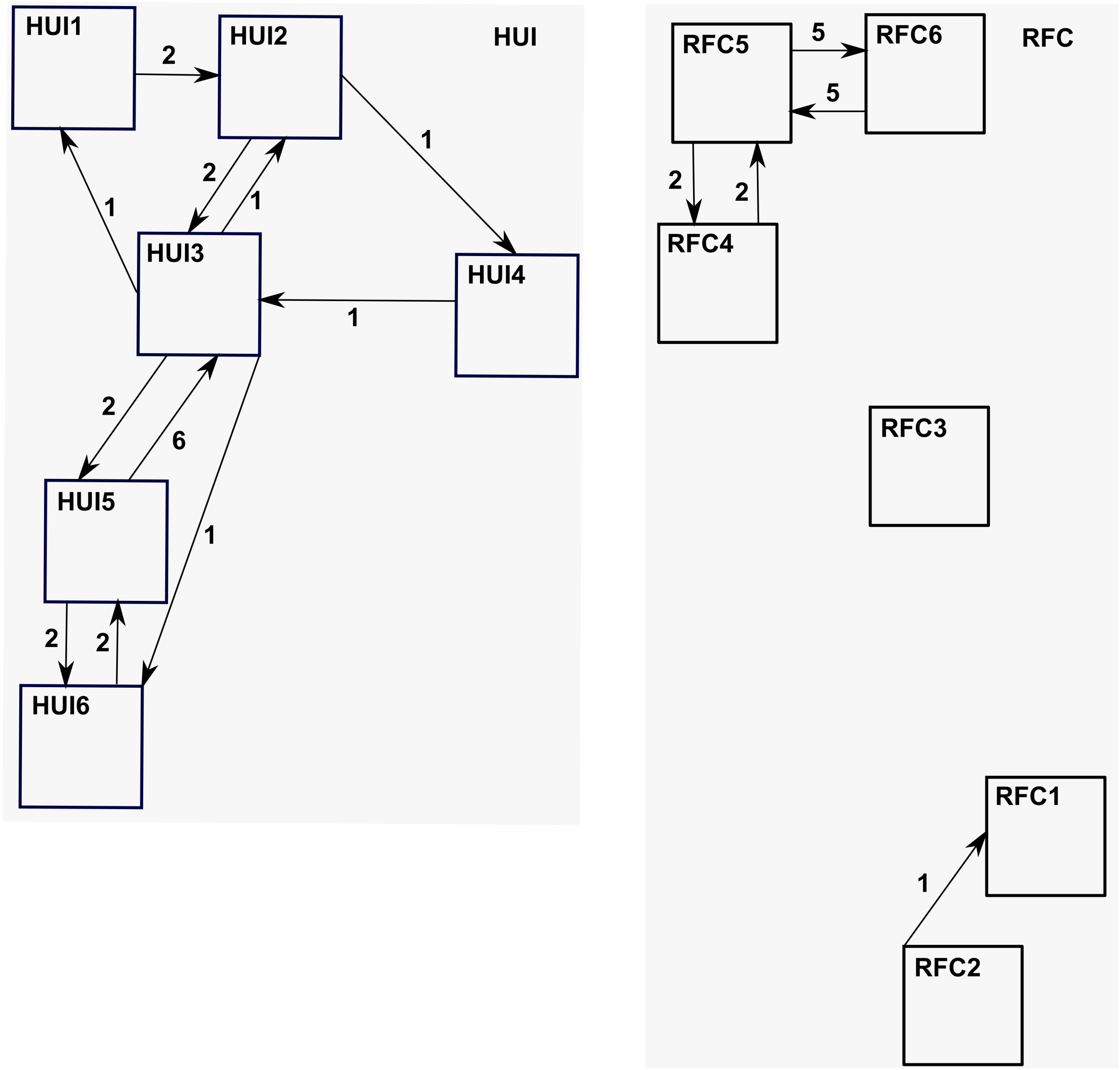


**Supplementary Figure** 46: Observed inter-plot dispersal events in *Rhinoderma darwinii* individuals from RFC and HUI. Arrows indicate the direction of dispersal, pointing to the destination plot, while numbers represent the observed dispersal events in that direction. Data were collected at RFC from November 2018 to March 2020, and at HUI from November 2018 to March 2022. The spatial arrangement of the 20 × 20 m plots closely resembles their real locations but has been slightly adjusted for better visualisation (see the main manuscript for the actual spatial representation).

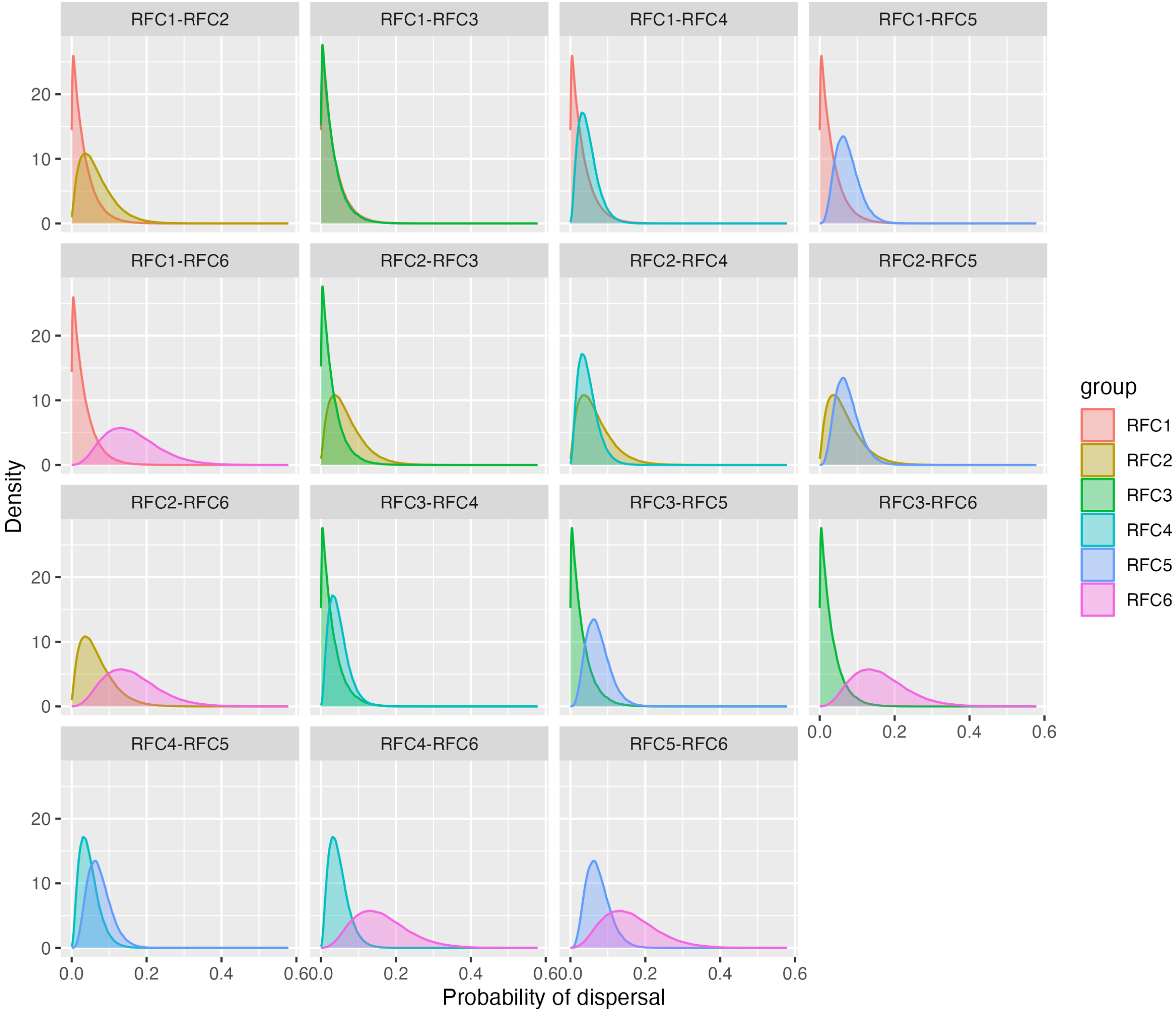


**Supplementary Figure** 47: Pairwise comparison of the posterior distribution of plot-level dispersal probability ($p_{\mathrm{disp},s}$) of *Rhinoderma darwinii* individuals at RFC.

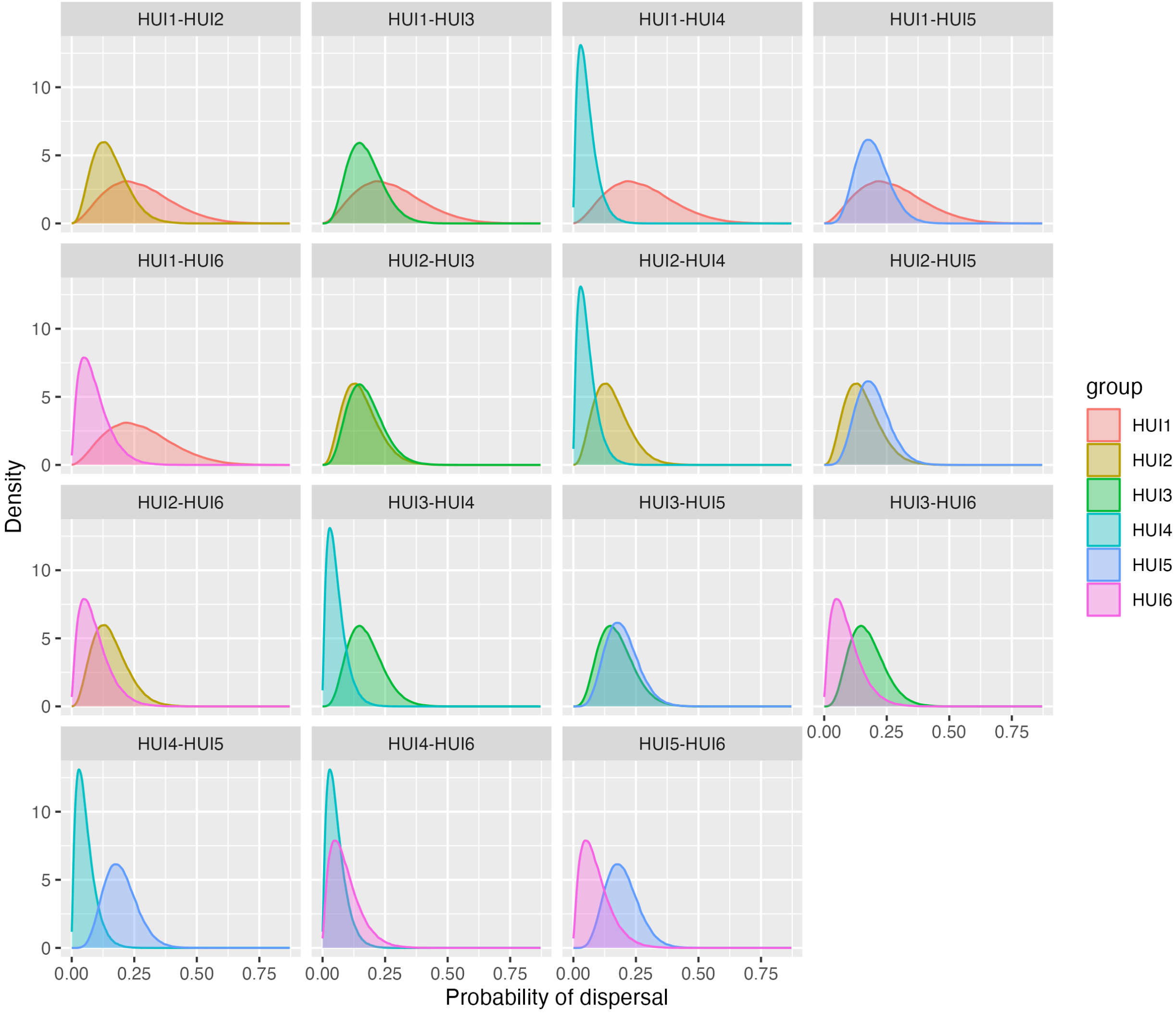


**Supplementary Figure** 48: Pairwise comparison of the posterior distribution of plot-level dispersal probability ($p_{\text{disp},s}$) of *Rhinoderma darwinii* individuals at HUI.

# Supplementary Tables

**Supplementary Table** 1: Probability of differing infection probabilities between pairs of local *Rhinoderma darwinii* populations in RFC. This probability is defined as the proportion of the posterior distributions of infection probability that do not overlap, calculated using the R package `Overlapping`. Proportions equal to or greater than 0.8 (i.e., strong or moderate evidence as defined in the main manuscript) are highlighted in bold.

| | **RFC1** | **RFC2** | **RFC3** | **RFC4** | **RFC5** | **RFC6** |
|---|---|---|---|---|---|---|
| **RFC1** | - | 0.251 | **0.931** | **0.992** | 0.335 | 0.447 |
| **RFC2** | - | - | **0.946** | **0.993** | 0.279 | 0.602 |
| **RFC3** | - | - | - | 0.344 | **0.986** | **0.797** |
| **RFC4** | - | - | - | - | **1.000** | **0.952** |
| **RFC5** | - | - | - | - | - | 0.729 |

**Supplementary Table** 2: Probability of differing infection probabilities between pairs of local *Rhinoderma darwinii* populations in HUI. This probability is defined as the proportion of the posterior distributions of infection probability that do not overlap, calculated using the R package `Overlapping`. Proportions equal to or greater than 0.8 (i.e., strong or moderate evidence as defined in the main manuscript) are highlighted in bold.

| | **HUI1** | **HUI2** | **HUI3** | **HUI4** | **HUI5** | **HUI6** |
|---|---|---|---|---|---|---|
| **HUI1** | - | 0.160 | 0.698 | 0.226 | 0.363 | 0.367 |
| **HUI2** | - | - | **0.825** | 0.358 | 0.218 | 0.518 |
| **HUI3** | - | - | - | 0.655 | **0.932** | 0.354 |
| **HUI4** | - | - | - | - | 0.568 | 0.308 |
| **HUI5** | - | - | - | - | - | 0.683 |

**Supplementary Table** 3: Pairwise comparison of environmental variables between plots, including mean temperature (Mean Temp), temperature fluctuation (Temp Var), mean relative humidity (Mean RH), and relative humidity fluctuation (RH Var). Fluctuations were calculated as the difference between the 99% and the 1% percentile of each variable during each month. The probability is defined as the proportion of the posterior distributions of the mean of each variable (from a Bayesian linear model where the monthly value of each variable was the dependent variable, while plot was used as a predictor) that do not overlap, calculated using the R package `Overlapping`. Probabilities equal to or greater than 0.8 (i.e., strong or moderate evidence as defined in the main manuscript) are highlighted in bold.

| **Plot 1** | **Plot 2** | **Mean Temp** | **Temp Var** | **Mean RH** | **RH Var** |
|---|---|---|---|---|---|
| RFC1 | RFC2 | 0.111 | 0.100 | 0.235 | 0.322 |
| RFC1 | RFC3 | 0.240 | 0.242 | 0.145 | 0.387 |
| RFC1 | RFC4 | 0.116 | 0.058 | 0.021 | 0.416 |
| RFC1 | RFC5 | 0.125 | 0.019 | 0.119 | 0.171 |
| RFC1 | RFC6 | 0.132 | 0.222 | 0.133 | 0.701 |
| RFC2 | RFC3 | 0.133 | 0.144 | 0.370 | 0.643 |
| RFC2 | RFC4 | 0.225 | 0.042 | 0.216 | 0.105 |
| RFC2 | RFC5 | 0.234 | 0.118 | 0.347 | 0.472 |
| RFC2 | RFC6 | 0.240 | 0.316 | 0.104 | 0.470 |
| RFC3 | RFC4 | 0.349 | 0.186 | 0.166 | 0.707 |
| RFC3 | RFC5 | 0.357 | 0.260 | 0.027 | 0.229 |
| RFC3 | RFC6 | 0.363 | 0.444 | 0.273 | **0.847** |
| RFC4 | RFC5 | 0.010 | 0.077 | 0.140 | 0.555 |
| RFC4 | RFC6 | 0.017 | 0.278 | 0.112 | 0.381 |
| RFC5 | RFC6 | 0.008 | 0.205 | 0.248 | **0.789** |
| HUI1 | HUI2 | 0.069 | 0.099 | 0.020 | 0.060 |
| HUI1 | HUI3 | 0.016 | 0.089 | 0.226 | 0.525 |
| HUI1 | HUI4 | 0.105 | 0.088 | 0.384 | **0.879** |
| HUI1 | HUI5 | 0.016 | 0.062 | 0.055 | 0.427 |
| HUI1 | HUI6 | 0.006 | 0.284 | 0.006 | **0.887** |
| HUI2 | HUI3 | 0.053 | 0.188 | 0.207 | 0.570 |
| HUI2 | HUI4 | 0.172 | 0.186 | 0.366 | **0.896** |
| HUI2 | HUI5 | 0.085 | 0.160 | 0.035 | 0.477 |
| HUI2 | HUI6 | 0.074 | 0.374 | 0.023 | **0.903** |
| HUI3 | HUI4 | 0.121 | **0.850** | 0.170 | 0.608 |

Continued on next page

**Supplementary Table** 3 (Continued)

| Plot 1 | Plot 2 | Mean Temp | Temp Var | Mean RH | RH Var |
|---|---|---|---|---|---|
| HUI3 | HUI5 | 0.032 | 0.028 | 0.173 | 0.120 |
| HUI3 | HUI6 | 0.021 | 0.198 | 0.228 | 0.626 |
| HUI4 | HUI5 | 0.089 | 0.026 | 0.335 | 0.685 |
| HUI4 | HUI6 | 0.099 | 0.199 | 0.386 | 0.027 |
| HUI5 | HUI6 | 0.012 | 0.224 | 0.058 | 0.701 |

**Supplementary Table** 4: Environmental variables calculated at the plot-level (subpopulation) in Field Study 1 for two spatially structured populations of *Rhinoderma darwinii* (RFC and HUI) in southern Chile.

| **Plot** | **Basal area** | **Air temperature** | | **Air relative Humidity** | |
|---|---|---|---|---|---|
| | ($m^2$ $ha^{-1}$)† | Mean (°C)‡ | Fluctuation‡ | Mean (%)‡ | Fluctuation‡ |
| RFC1 | 61.8 | 13.6 | 12.1 | 85.1 | 28.6 |
| RFC2 | 87.7 | 13.4 | 11.9 | 83.6 | 31.1 |
| RFC3 | 59.0 | 13.1 | 11.7 | 86.1 | 25.6 |
| RFC4 | 60.6 | 13.8 | 12.0 | 85.0 | 32.0 |
| RFC5 | 71.9 | 13.8 | 12.1 | 85.9 | 27.3 |
| RFC6 | 78.1 | 13.8 | 12.4 | 84.3 | 34.9 |
| HUI1 | 48.4 | 12.8 | 16.2 | 85.5 | 23.2 |
| HUI2 | 36.1 | 12.5 | 15.9 | 85.4 | 22.6 |
| HUI3 | 60.9 | 12.7 | 16.9 | 83.9 | 28.4 |
| HUI4 | 69.1 | 13.3 | 17.1 | 82.4 | 34.5 |
| HUI5 | 56.0 | 12.9 | 16.8 | 85.0 | 27.9 |
| HUI6 | 33.0 | 12.8 | 17.8 | 85.4 | 35.1 |

† Only trees with a DBH > 5 cm were included in the calculation of tree basal area (Gutiérrez et al. 2019).

‡ Calculated using 20,250 observations (RFC = 9,714; HUI = 10,536) collected during 8 months (normally across 5–6 days per month).

**Supplementary Table** 5: Epidemiological and demographic metrics explored in the IBM.

| **Parameter** | **Description** |
|---|---|
| $\delta$ | Population depression: the proportional reduction in observed population size due to Bd infection relative to a no-pathogen scenario. |
| $Bd_{\mathrm{fadeout}}$ | Binary indicator variable set to 1 if no Bd-infected individuals are present at a given time (indicating pathogen extirpation), and 0 otherwise. |
| $n_{\mathrm{epidemic,rd}}$ | Number of epidemic months, defined as months with $R_{\mathrm{eff,rd}}(t) > 1$. |
| $\rho_{\mathrm{rd}}$ | Population growth rate (%) of *R. darwinii*, calculated as the percentage change in population size from $t = 1$ to $t = 12$. |
| $\rho_{\mathrm{syn}}$ | Population growth rate (%) of syntopic amphibians, calculated as the percentage change in population size from $t = 1$ to $t = 12$. |
| $p_{inf_{\mathrm{mean,rd}}}$ | Mean monthly infection probability for *R. darwinii* ($PrInf_{\mathrm{rd}}(t)$) over the simulation period (excluding $t = 1$). |
| $p_{inf_{\mathrm{mean,rd}}}$ | Maximum infection probability observed in *R. darwinii* ($PrInf_{\mathrm{rd}}(t)$)during the simulation (excluding $t = 1$). |
| $t_{\mathrm{max},PrInf_{\mathrm{rd}}}$ | Month at which $PrInf_{\mathrm{rd}}(t)$ attains its maximum value. |
| $\mathrm{Var}(PrInf_{\mathrm{rd}})$ | Variance of $PrInf_{\mathrm{rd}}(t)$ over the simulation period (excluding $t = 1$). |
| $R_{0,\mathrm{rd}}$ | Ferrari's basic reproduction number in *R. darwinii.* |
| $R_0$ | Ferrari's basic reproduction number in *R. darwinii* and syntopic species. |
| $R_{\mathrm{t,rd}}$ | Epidemic ratio in *R. darwinii*: the ratio of new infections in month $t + 1$ to the number of infected individuals in month $t$. |
| $\bar{R}_{\mathrm{t,rd}}$ | Geometric mean of $R_{\mathrm{t,rd}}$ over the simulation period. |
| $R^{\mathrm{max}}_{\mathrm{t,rd}}$ | Maximum value of $R_{\mathrm{t,rd}}$ observed during the simulation. |
| $t_{\mathrm{max},R_{\mathrm{t,rd}}}$ | Month at which $R_{\mathrm{t,rd}}$ attains its maximum value. |
| $\mathrm{Var}(R_{\mathrm{t,rd}})$ | Variance of $R_{\mathrm{t,rd}}$ over the simulation period. |
| $R_{\mathrm{t}}$ | Epidemic ratio for both *R. darwinii* and syntopic species. |
| $\bar{R}_{\mathrm{t}}$ | Geometric mean of $R_{\mathrm{t}}$ over the simulation period. |
| $R^{\mathrm{max}}_{\mathrm{t}}$ | Maximum value of $R_{\mathrm{t}}$ observed during the simulation. |
| $t_{\mathrm{max},R_{\mathrm{t}}}$ | Month at which $R_{\mathrm{t}}$ attains its maximum value. |
| $\mathrm{Var}(R_{\mathrm{t}})$ | Variance of $R_{\mathrm{t}}$ over the simulation period. |
| $f_{\mathrm{ever,inf,rd}}$ | Proportion of *R. darwinii* individuals that become infected at least once during the simulation. |
| $f_{\mathrm{ever,inf,syn}}$ | Proportion of syntopic amphibians that become infected at least once during the simulation. |
| $\bar{p}_{\mathrm{rd}}$ | Mean Bd prevalence in *R. darwinii* over all time steps (excluding $t = 1$). |
| $\bar{p}_{\mathrm{syntopic}}$ | Mean Bd prevalence in syntopic amphibians over all time steps (excluding $t = 1$). |
| $p_{\mathrm{max,rd}}$ | Maximum Bd prevalence observed in *R. darwinii* during the simulation (excluding $t = 1$). |
| $p_{\mathrm{max,syntopic}}$ | Maximum Bd prevalence observed in syntopic amphibians during the simulation (excluding $t = 1$). |

**Supplementary Table** 6: Input parameters included in the IBM function.

| Parameter | Description | Default value | Perturbation in GSA (R code) | Source |
|---|---|---|---|---|
| $\lambda_{\text{rd}}$ | Expected number of *R. darwinii* individuals per unit area (frogs $\text{m}^{-2}$). | 0.13 | `parameter * rlnorm(meanlog = 0, sdlog = 0.3)` | This study. |
| $\lambda_{\text{syntopic}}$ | Expected number of syntopic amphibians per unit area (frogs $\text{m}^{-2}$). | 0.12 | `parameter * rlnorm(meanlog = 0, sdlog = 0.3)` | This study. |
| $\text{variance}_{\text{LGCP}}$ | Variance of the Gaussian random field in the LGCP model. | 3.540 | `parameter * rlnorm(meanlog = 0, sdlog = 0.3)` | This study; average from RFC, HUI, and TAN. |
| $\text{scale}_{\text{LGCP}}$ | Scale of the Gaussian random field in the LGCP model. | 1.510 | `parameter * rlnorm(meanlog = 0, sdlog = 0.3)` | This study; average from RFC, HUI, and TAN. |
| $\sigma_{\text{juv}}$ | Standard deviation of x- and y-direction movement distances for juveniles. | 5.045 | `parameter * rlnorm(meanlog = 0, sdlog = 0.3)` | This study; average from RFC and HUI. |
| $\sigma_{\text{adu}}$ | Standard deviation of x- and y-direction movement distances for adults. | 5.021 | `parameter * rlnorm(meanlog = 0, sdlog = 0.3)` | This study; average from RFC and HUI. |
| $\alpha_{p_{\text{inf,rd}}}$ | Intercept of a logistic regression that models the probability of infection as a function of the log-transformed shortest distance of individual $i$ to a Bd-positive individual in *R. darwinii*. | $-0.700$ | `parameter * rlnorm(meanlog = 0, sdlog = 0.3)` | This study; default estimated at RFC. |
| $\beta_{p_{\text{inf,rd}}}$ | Slope of a logistic regression that models the probability of infection as a function of the log-transformed shortest distance of individual $i$ to a Bd-positive individual in *R. darwinii*. | $-1.178$ | `parameter * rlnorm(meanlog = 0, sdlog = 0.3)` | This study; default estimated at RFC. |
| $\alpha_{p_{\text{inf,syntopic}}}$ | Intercept of a logistic regression that models the probability of infection as a function of the log-transformed shortest distance of individual $i$ to a Bd-positive individual in syntopic species. | $-0.700$ | `parameter * rlnorm(meanlog = 0, sdlog = 0.3)` | Used same as in *R. darwinii*. |
| $\beta_{p_{\text{inf,syntopic}}}$ | Slope of a logistic regression that models the probability of infection as a function of the log-transformed shortest distance of individual $i$ to a Bd-positive individual in syntopic species. | $-1.178$ | `parameter * rlnorm(meanlog = 0, sdlog = 0.3)` | Used same as in *R. darwinii*. |
| $p_{\text{base}}$ | Background probability of Bd infection in the absence of Bd-positive individuals. | 0 | NA | NA |
| $p_{\text{recov,rd}}$ | Probability of recovery from Bd infection in *R. darwinii*. | 0.213 | `plogis(qlogis(parameter) + rnorm(mean = 0, sd = 0.3))` | This study; estimated at RFC. |
| $p_{\text{recov,syntopic}}$ | Probability of recovery from Bd infection in syntopic species. | 0.213 | `plogis(qlogis(parameter) + rnorm(mean = 0, sd = 0.3))` | Used same as in *R. darwinii*. |
| $\phi_{\text{rd,juv,neg}}$ | Monthly survival probability for Bd-negative *R. darwinii* juveniles. | 0.962 | `plogis(qlogis(parameter) + rnorm(mean = 0, sd = 0.3))` | This study; average from RFC and HUI. |
| $\phi_{\text{rd,juv,pos}}$ | Monthly survival probability for Bd-positive *R. darwinii* juveniles. | 0.317 | `plogis(qlogis(parameter) + rnorm(mean = 0, sd = 0.3))` | This study; average from RFC and HUI. |
| $\phi_{\text{rd,adu,neg}}$ | Monthly survival probability for Bd-negative *R. darwinii* adults. | 0.987 | `plogis(qlogis(parameter) + rnorm(mean = 0, sd = 0.3))` | This study; average from RFC and HUI. |
| $\phi_{\text{rd,adu,pos}}$ | Monthly survival probability for Bd-positive *R. darwinii* adults. | 0.403 | `plogis(qlogis(parameter) + rnorm(mean = 0, sd = 0.3))` | This study; average from RFC and HUI. |
| $\phi_{\text{syntopic,neg}}$ | Monthly survival probability for Bd-negative syntopic amphibians. | 0.960 | `plogis(qlogis(parameter) + rnorm(mean = 0, sd = 0.3))` | This study; estimated at RFC. |
| $\phi_{\text{syntopic,pos}}$ | Monthly survival probability for Bd-positive syntopic amphibians. | 0.960 | `plogis(qlogis(parameter) + rnorm(mean = 0, sd = 0.3))` | This study; equal to $\phi_{\text{syntopic,neg}}$. |
| $p_{\text{juv}}$ | Proportion of juveniles in the population. | 0.38 | `plogis(qlogis(parameter) + rnorm(mean = 0, sd = 0.3))` | Average from values in Valenzuela-Sánchez et al. (2022). |

**Supplementary Table 7**: Models used in the global sensitivity analysis.

| **Parameter** | **Machine learning model** | **GLM-based model** |
|---|---|---|
| $\delta$ | Random forest with scaled (z-transformed) predictors | Beta regression with logit link function and scaled (z-transformed) predictors |
| $R_0$ | Random forest with scaled (z-transformed) predictors | Gamma GLM with log link function and scaled (z-transformed) predictors |
| $Bd_{\text{fadeout}}$ | Random forest with scaled (z-transformed) predictors | Logistic regression with scaled (z-transformed) predictors |
| $n_{\text{epidemic,rd}}$ | Random forest with scaled (z-transformed) predictors | Negative binomial GLM with scaled (z-transformed) predictors |

**Supplementary Table** 8: Abundance (and Bayesian 95% CRI) and density (frogs m$^{-2}$) estimated at each population during this study using a closed capture-recapture model with detectability modelled as constant. Mean values are the average from all primary capture occasions, while the maximum values represent the capture occasion with the highest density for each population.

| **Plot** | **Mean abundance** | **Mean density** | **Max. abundance** | **Max. density** |
|---|---|---|---|---|
| RFC1 | 26 (20–34) | 0.06 | 41 (25–59) | 0.10 |
| RFC2 | 21 (15–28) | 0.05 | 34 (19–69) | 0.09 |
| RFC3 | 22 (17–29) | 0.06 | 36 (23–67) | 0.09 |
| RFC4 | 45 (38–52) | 0.11 | 59 (43–88) | 0.15 |
| RFC5 | 38 (33–46) | 0.10 | 53 (37–82) | 0.13 |
| RFC6 | 21 (14–30) | 0.05 | 36 (15–68) | 0.09 |
| HUI1 | 12 (6–18) | 0.03 | 19 (5–60) | 0.05 |
| HUI2 | 17 (12–24) | 0.04 | 37 (12–69) | 0.09 |
| HUI3 | 17 (12–24) | 0.04 | 33 (9–66) | 0.08 |
| HUI4 | 19 (14–25) | 0.05 | 54 (29–82) | 0.14 |
| HUI5 | 21 (15–28) | 0.05 | 36 (10–67) | 0.09 |
| HUI6 | 15 (10–21) | 0.04 | 28 (12–53) | 0.07 |
| TAN1 | 87 (79–97) | 0.12 | 143 (115–177) | 0.20 |
| TAN2 | 41 (37–46) | 0.08 | 72 (59–92) | 0.13 |

**Supplementary Table** 9: Number of individuals removed from the fenced areas in the exclusion experiments in RFC and HUI. EC = *Eupsophus contulmoensis/roseus*, EV = *Eupsophus vertebralis*, BL = *Batrachyla leptopus*, PT = *Pleurodema thaul*.

| **Month** | **Exclusion** | **Area** | **EC** | **EV** | **BL** | **PT** |
|---|---|---|---|---|---|---|
| Nov 2018 | EXCHUI1 | HUI | 1 | 0 | 0 | 0 |
| Dec 2018 | EXCHUI1 | HUI | 0 | 0 | 0 | 0 |
| Jan 2019 | EXCHUI1 | HUI | 0 | 0 | 1 | 1 |
| Feb 2019 | EXCHUI1 | HUI | 0 | 0 | 5 | 0 |
| Mar 2019 | EXCHUI1 | HUI | 1 | 0 | 0 | 1 |
| | | Total | 2 | 0 | 6 | 2 |
| Nov 2018 | EXCHUI2 | HUI | 0 | 0 | 0 | 0 |
| Dec 2018 | EXCHUI2 | HUI | 0 | 0 | 0 | 0 |
| Jan 2019 | EXCHUI2 | HUI | 0 | 0 | 1 | 0 |
| Feb 2019 | EXCHUI2 | HUI | 0 | 0 | 3 | 0 |
| Mar 2019 | EXCHUI2 | HUI | 0 | 0 | 1 | 0 |
| Jan 2021 | EXCHUI2 | HUI | 0 | 0 | 1 | 0 |
| | | Total | 0 | 0 | 6 | 0 |
| Nov 2018 | EXCMNC | RFC | 11 | 0 | 0 | 0 |
| Dec 2018 | EXCMNC | RFC | 6 | 0 | 0 | 0 |
| Jan 2019 | EXCMNC | RFC | 5 | 0 | 0 | 0 |
| Feb 2019 | EXCMNC | RFC | 4 | 0 | 2 | 0 |
| Mar 2019 | EXCMNC | RFC | 6 | 0 | 0 | 0 |
| Nov 2019 | EXCMNC | RFC | 2 | 0 | 0 | 0 |
| Jan 2020 | EXCMNC | RFC | 4 | 0 | 0 | 0 |
| Mar 2020 | EXCMNC | RFC | 5 | 0 | 0 | 0 |
| | | Total | 43 | 0 | 2 | 0 |

**Supplementary Table** 10: Observed dispersal events in *Rhinoderma darwinii* across RFC and HUI. The table includes the frog ID, number of captures, plots between which dispersal occurred, and the distance in metres between the centre of the plot of departure and arrival.

| Frog ID | No. Captures | Plots | Distance (m) |
|---|---|---|---|
| a177 | 4 | RFC4–RFC5 | 31.61 |
| a132 | 4 | RFC6–RFC5 | 23.42 |
| b183 | 3 | RFC5–RFC4 | 31.61 |
| b369 | 6 | RFC5–RFC6 | 23.42 |
| b369 | 6 | RFC6–RFC5 | 23.42 |
| b168 | 4 | RFC6–RFC5 | 23.42 |
| b168 | 4 | RFC5–RFC6 | 23.42 |
| b168 | 4 | RFC6–RFC5 | 23.42 |
| c186 | 5 | RFC4–RFC5 | 31.61 |
| c186 | 5 | RFC5–RFC4 | 31.61 |
| c321 | 3 | RFC6–RFC5 | 23.42 |
| c321 | 3 | RFC5–RFC6 | 23.42 |
| d045 | 2 | RFC5–RFC6 | 23.42 |
| d116 | 2 | RFC5–RFC6 | 23.42 |
| g237 | 4 | RFC2–RFC1 | 31.84 |
| a053 | 5 | HUI1–HUI2 | 34.02 |
| a015 | 3 | HUI4–HUI3 | 55.11 |
| a072 | 10 | HUI5–HUI6 | 34.02 |
| a072 | 10 | HUI6–HUI5 | 34.02 |
| a072 | 10 | HUI5–HUI6 | 34.02 |
| b094 | 6 | HUI5–HUI3 | 54.63 |
| b136 | 3 | HUI6–HUI5 | 34.02 |
| c050 | 2 | HUI2–HUI3 | 27.60 |
| c138 | 6 | HUI3–HUI5 | 54.63 |
| c138 | 6 | HUI5–HUI3 | 54.63 |
| d229 | 9 | HUI5–HUI3 | 54.63 |
| d229 | 9 | HUI3–HUI5 | 54.63 |
| e157 | 3 | HUI3–HUI1 | 32.30 |
| h1 | 4 | HUI5–HUI3 | 54.63 |
| j103 | 2 | HUI5–HUI3 | 54.63 |
| k84 | 5 | HUI2–HUI4 | 55.30 |
| k87 | 2 | HUI3–HUI6 | 88.41 |
| l1 | 2 | HUI2–HUI3 | 27.60 |

Continued on next page

**Supplementary Table** 10 (Continued)

| Frog ID | No. Captures | Plots | Distance (m) |
|---|---|---|---|
| p72 | 2 | HUI1–HUI2 | 34.02 |
| q23 | 3 | HUI3–HUI2 | 27.60 |
| r81 | 2 | HUI5–HUI3 | 54.63 |

**Supplementary Table** 11: Estimated *Rhinoderma darwinii* dispersal probabilities for each plot ($p_{\text{disp},s}$) within RFC and HUI. Values represent posterior means with 95% credible intervals (CRI) in parentheses.

| **Area** | **Plot** | **Dispersal probability** |
|---|---|---|
| RFC | RFC1 | 0.032 (0.001–0.116) |
| RFC | RFC2 | 0.067 (0.008–0.177) |
| RFC | RFC3 | 0.030 (0.001–0.109) |
| RFC | RFC4 | 0.047 (0.010–0.110) |
| RFC | RFC5 | 0.074 (0.025–0.146) |
| RFC | RFC6 | 0.160 (0.048–0.323) |
| HUI | HUI1 | 0.273 (0.067–0.557) |
| HUI | HUI2 | 0.154 (0.045–0.311) |
| HUI | HUI3 | 0.172 (0.061–0.327) |
| HUI | HUI4 | 0.055 (0.007–0.149) |
| HUI | HUI5 | 0.194 (0.084–0.337) |
| HUI | HUI6 | 0.091 (0.012–0.238) |
