# Supplementary material for: Localized transmission of an aquatic pathogen drives hidden epidemics and population collapse in a terrestrial host
Source: Nat Ecol Evol. 2026 Jan 2;10(2):308–17. doi: 10.1038/s41559-025-02930-1 (PMC12890588; doi:10.1038/s41559-025-02930-1)
Supplement: Supplementary file 2 — Reporting Summary [file 41559_2025_2930_MOESM2_ESM.pdf]

nature portfolio

Corresponding author(s): Andrés Valenzuela-Sánchez

Last updated by author(s): Oct 17, 2025

Reporting Summary

Nature Portfolio wishes to improve the reproducibility of the work that we publish. This form provides structure for consistency and transparency in reporting. For further information on Nature Portfolio policies, see our Editorial Policies and the Editorial Policy Checklist.

Statistics

For all statistical analyses, confirm that the following items are present in the figure legend, table legend, main text, or Methods section.

n/a	Confirmed	
☐	☒	The exact sample size (*n*) for each experimental group/condition, given as a discrete number and unit of measurement
☐	☒	A statement on whether measurements were taken from distinct samples or whether the same sample was measured repeatedly
☐	☒	The statistical test(s) used AND whether they are one- or two-sided<br>*Only common tests should be described solely by name; describe more complex techniques in the Methods section.*
☐	☒	A description of all covariates tested
☐	☒	A description of any assumptions or corrections, such as tests of normality and adjustment for multiple comparisons
☐	☒	A full description of the statistical parameters including central tendency (e.g. means) or other basic estimates (e.g. regression coefficient) AND variation (e.g. standard deviation) or associated estimates of uncertainty (e.g. confidence intervals)
☐	☒	For null hypothesis testing, the test statistic (e.g. *F*, *t*, *r*) with confidence intervals, effect sizes, degrees of freedom and *P* value noted<br>*Give P values as exact values whenever suitable.*
☐	☒	For Bayesian analysis, information on the choice of priors and Markov chain Monte Carlo settings
☐	☒	For hierarchical and complex designs, identification of the appropriate level for tests and full reporting of outcomes
☒	☐	Estimates of effect sizes (e.g. Cohen's *d*, Pearson's *r*), indicating how they were calculated

Our web collection on statistics for biologists contains articles on many of the points above.

Software and code

Policy information about availability of computer code

Data collection	R (version 4.4.2) was used for data collection
Data analysis	R (version 4.4.2) and JAGS (version 4.3.1) were used for data analysis. The data for reproducing the analyses from this study are available via Zenodo at https://doi.org/10.5281/zenodo.17148244.

For manuscripts utilizing custom algorithms or software that are central to the research but not yet described in published literature, software must be made available to editors and reviewers. We strongly encourage code deposition in a community repository (e.g. GitHub). See the Nature Portfolio guidelines for submitting code & software for further information.

Data

Policy information about availability of data

All manuscripts must include a data availability statement. This statement should provide the following information, where applicable:

- Accession codes, unique identifiers, or web links for publicly available datasets
- A description of any restrictions on data availability
- For clinical datasets or third party data, please ensure that the statement adheres to our policy

The code used for data analysis is available via Zenodo at https://doi.org/10.5281/zenodo.17148244.

Research involving human participants, their data, or biological material

Policy information about studies with human participants or human data. See also policy information about sex, gender (identity/presentation), and sexual orientation and race, ethnicity and racism.

Reporting on sex and gender	*Use the terms sex (biological attribute) and gender (shaped by social and cultural circumstances) carefully in order to avoid confusing both terms. Indicate if findings apply to only one sex or gender; describe whether sex and gender were considered in study design; whether sex and/or gender was determined based on self-reporting or assigned and methods used. Provide in the source data disaggregated sex and gender data, where this information has been collected, and if consent has been obtained for sharing of individual-level data; provide overall numbers in this Reporting Summary. Please state if this information has not been collected. Report sex- and gender-based analyses where performed, justify reasons for lack of sex- and gender-based analysis.*
Reporting on race, ethnicity, or other socially relevant groupings	*Please specify the socially constructed or socially relevant categorization variable(s) used in your manuscript and explain why they were used. Please note that such variables should not be used as proxies for other socially constructed/relevant variables (for example, race or ethnicity should not be used as a proxy for socioeconomic status). Provide clear definitions of the relevant terms used, how they were provided (by the participants/respondents, the researchers, or third parties), and the method(s) used to classify people into the different categories (e.g. self-report, census or administrative data, social media data, etc.) Please provide details about how you controlled for confounding variables in your analyses.*
Population characteristics	*Describe the covariate-relevant population characteristics of the human research participants (e.g. age, genotypic information, past and current diagnosis and treatment categories). If you filled out the behavioural & social sciences study design questions and have nothing to add here, write "See above."*
Recruitment	*Describe how participants were recruited. Outline any potential self-selection bias or other biases that may be present and how these are likely to impact results.*
Ethics oversight	*Identify the organization(s) that approved the study protocol.*

Note that full information on the approval of the study protocol must also be provided in the manuscript.

Field-specific reporting

Please select the one below that is the best fit for your research. If you are not sure, read the appropriate sections before making your selection.

☐ Life sciences ☐ Behavioural & social sciences ☒ Ecological, evolutionary & environmental sciences

For a reference copy of the document with all sections, see nature.com/documents/nr-reporting-summary-flat.pdf

Ecological, evolutionary & environmental sciences study design

All studies must disclose on these points even when the disclosure is negative.

Study description	This article integrates novel empirical data derived from two distinct field studies and one in-silico study. Field Study 1 comprises observational research on two spatially structured populations of Rhinoderma darwinii located in Contulmo (RFC) and Neltume (HUI), southern Chile. In each of these spatially structured populations, six permanent plots, each covering 400 square metres, were established within the forest to collect spatial capture-recapture data. Field Study 2 involves the long-term epidemiological and demographic monitoring of two local populations (TAN1 and TAN2) of R. darwinii from Inio, Chiloé Island, southern Chile, with permanent study plots measuring 700 and 538 square metres, respectively.
Research sample	All amphibians found inside the study plots during visual encounter surveys were captured, this included R. darwinii, Eupsophus contulmoensis, E. roseus, E. vertebralis, and Batrachyla leptopus.
Sampling strategy	Capture-recapture methods require to capture all individuals observed during a given searching period. In terms of statistical power, the critical aspect is the number of recaptures per individual, as capture-recapture models can generally provide robust parameter estimates even with small sample sizes (e.g. 10-15 individuals per local population) if return rates are relatively high. For our focal species, R. darwinii, recapture probability is generally moderate to high (between 0.3 and 0.6) with the searching effort and methodology used in our study, allowing us to obtain good estimates for most of the parameters of interest.
Data collection	Two researchers surveyed each plot for 30 min to 1 hour per day depending on the site. Rhinoderma darwinii is a diurnal species, so we visually surveyed each site during daylight hours in a manner that covered the entire plot with an apparently equal search effort throughout the site. all captured frogs (R. darwinii and syntopic amphibians) were measured (snout-to-vent length), skin-swabbed for Batrachochytrium dendrobatidis infection detection and skin bacteriome characterisation (the latter in RFC only), photographed for individual recognition using their colouration patterns, and released at the exact point of capture. In RFC and HUI, each study plot was permanently delimited using plastic strings placed every 10 m, allowing us to record the spatial location (x- and y-coordinates) of each capture with a ±10 cm precision.
Timing and spatial scale	Field Study 1 was conducted from 2018 to 2022. The study was concluded prematurely at RFC after March 2020 due to a surge in violent attacks in the area, including an incendiary attack on our research team at the study site. From November to March each

	year, each plot was surveyed once per month (i.e., primary capture occasion) during three consecutive days (i.e., secondary capture occasions), totalling 10 and 19 primary capture occasions in RFC and HUI, respectively. Field Study 2 was conducted from 2014 to 2025. As above, the capture-recapture data were collected at two nested levels of capture occasions. Each year, we carried out one primary survey period in early summer during the peak of the reproductive season (January-February). During each primary period, we conducted three to four secondary survey occasions, each consisting of a 1-hour daily survey per plot on consecutive days. Additionally, during 2010, 2011, and 2014 we surveyed amphibians and sampled them for B. dendrobatidis infection in eight additional study plots near to TAN1 and TAN2 using the same methodology. The spatial scale of the study plots is described in 'Study description' above.
Data exclusions	No data were excluded from the analyses.
Reproducibility	To ensure reproducibility of this field study, detailed methodological protocols—including the spatial arrangement of study plots, sampling methodology, data collection procedures, and data analysis scripts—have been provided. All data and code required to replicate our findings are publicly available in a dedicated repository (https://doi.org/10.5281/zenodo.17148244), enabling independent verification and reproducibility of the analyses.
Randomization	This study is observational and based on naturally occurring spatially structured populations. No experimental treatments were applied, and hence randomization of individuals or groups was not applicable to this study.
Blinding	Given the observational nature of this field study, full blinding was not possible during data collection; however, fieldworkers were unaware of the Bd infection status of the captured animals. For laboratory analyses, personnel had access to sample codes and were generally unaware of the species, age, sex, or other characteristics of the animals during DNA extraction and PCR analysis.

Did the study involve field work? ☒ Yes ☐ No

Field work, collection and transport

Field conditions	This study was conducted in the Austral temperate forests of Chile, where air temperatures typically range from approximately 5°C to 25°C. Surveys were not conducted during periods of heavy rain or strong winds, as such conditions reduce amphibian detectability and pose an unacceptable risk to fieldworkers.
Location	The coordinates of the study sites are: RFC (38°01'50.9''S 73°12'17.0''W), HUI (39°52'21.4''S 71°54'49.2''W), TAN1 (43°21'36.13"S 74°6'20.87"W), TAN2 (43°21'31.60"S 74°6'41.30"W).
Access & import/export	Fieldworkers took special care to minimise habitat disturbance during surveys by walking slowly and avoiding damage to vegetation and substrates. Footwear was always cleaned and disinfected with a 1% Virkon S (10 g/L) solution before entering any study site, even if disinfection had already been carried out at a nearby site earlier that same day. Disinfection of footwear and equipment was consistently performed when moving between study sites located more than 1 km apart; in areas with a high risk of B. dendrobatidis spread, this distance threshold was reduced. Other gear and vehicle tyres were cleaned and disinfected with a 1% Virkon S solution when necessary (e.g., in the presence of mud).This research was conducted in accordance with Chilean law under permits no. 5666/2013, no. 230/2015, no. 212/2016, no. 1997/2016, no. 1695/2018, no. 6618/2019, no. 7669/2020, no. 226/2021, no. 6488/2021, no. 7161/2022, no. 7163/2022, no. 8157/2023, no. 6001/2024 of the Servicio Agrícola y Ganadero de Chile, and no. 04/2018 IX, no. 10/2018 IX, and no. 10/2020 IX of the Corporación Nacional Forestal de Chile.
Disturbance	We did not observe any evident disturbance to the habitat or the sampled animals.

Reporting for specific materials, systems and methods

We require information from authors about some types of materials, experimental systems and methods used in many studies. Here, indicate whether each material, system or method listed is relevant to your study. If you are not sure if a list item applies to your research, read the appropriate section before selecting a response.

Materials & experimental systems

n/a	Involved in the study	
☒	☐	Antibodies
☒	☐	Eukaryotic cell lines
☒	☐	Palaeontology and archaeology
☐	☒	Animals and other organisms
☒	☐	Clinical data
☒	☐	Dual use research of concern
☒	☐	Plants

Methods

n/a	Involved in the study	
☒	☐	ChIP-seq
☒	☐	Flow cytometry
☒	☐	MRI-based neuroimaging

Animals and other research organisms

Policy information about studies involving animals; ARRIVE guidelines recommended for reporting animal research, and Sex and Gender in Research

Laboratory animals	This study did not involve laboratory animals.
Wild animals	We made 2,672 captures of 1,415 Rhinoderma darwinii individuals (RFC: 419, HUI: 339, TAN: 657). We also made 383 captures from 352 syntopic amphibians (RFC: 216, HUI: 12: TAN: 124), including eight species (RFC: Contulmo Ground Frog [Eupsophus contulmoensis] and Valdivia Ground Frog [Eupsophus vertebralis]; HUI: Grey Wood Frog [Batrachyla leptopus] and Rosy Ground Frog [Eupsophus roseus]; TAN: Grey Wood Frog [Batrachyla leptopus], Banded Wood Frog [Batrachyla taeniata], Emerald Forest Frog [Hylorina sylvatica], Chiloe Island Ground Frog [Eupsophus calcaratus] and Emilio's Ground Frog [Eupsophus emiliopugini]) found inside the study plots. All post-metamorphic amphibians found during the searches were captured, without differentiating by sex.<br><br>These procedures applied to all amphibian species captured in this study.<br><br>Source of animals / capture techniques:<br>All amphibians were studied in the field using visual encounter surveys in plots. During each survey period, each site was generally surveyed daily on three or four consecutive days by two researchers, in a manner that covered the entire plot with an apparently equal search effort throughout the site. Any frogs seen were captured by hand while wearing a new pair of disposable, powder-free nitrile gloves. Captured frogs were maintained individually in clean, disposable plastic bags filled with air and kept out of direct sunlight until they were processed. Only post-metamorphic individuals were captured.<br><br>Handling:<br>Each amphibian was always handled using clean, disposable, powder-free nitrile gloves. A pair of gloves was used to handle only one individual and was then safely disposed of.<br><br>Sampling:<br>Sampling consisted of the procedures detailed below. Importantly, sampling lasted no more than five minutes per individual, and animals were always handled while wearing a new pair of clean, powder-free nitrile gloves.<br><br>Morphometric measures and identification: Captured animals were measured (snout-to-vent length, SVL) using digital callipers, weighed using a digital scale, and photographed for individual identification using unique colouration patterns.<br><br>B. dendrobatidis detection from amphibian skin: We used a sterile, dry, rayon-tipped swab (MW100, Medical & Wire Equipment Co.) to sample for B. dendrobatidis DNA that may have been present on the skin of captured frogs. For this, a new swab was firmly run five times each over the ventral abdomen and pelvis, each ventral hind limb (femur and tibia), and the plantar surface of each hind foot, to complete a total of 35 strokes per individual. Using a second swab, this process was immediately repeated in some individuals to estimate pathogen detectability. Each frog was sampled for B. dendrobatidis detection no more than once per month (either using a single swab or two swabs in tandem), up to five times per year.<br><br>Release:<br>After sampling, each amphibian was released at the exact point of capture, which had been marked using a coloured peg. Release always took place within 3 hours of capture.
Reporting on sex	Sex-specific analyses were not conducted in this study, and sampling was not biased by sex.
Field-collected samples	Skin swabs were stored at ambient temperature in a dry, cool place in the field, and stored at –20°C in the lab until processing.
Ethics oversight	This research was approved by the ethics committees at Universidad Austral de Chile (no. 305/2018), Universidad Andrés Bello (no. 13/2015), and Zoological Society of London (no. WLE709 and no. IOZ222).

Note that full information on the approval of the study protocol must also be provided in the manuscript.

Plants

Seed stocks	*Report on the source of all seed stocks or other plant material used. If applicable, state the seed stock centre and catalogue number. If plant specimens were collected from the field, describe the collection location, date and sampling procedures.*
Novel plant genotypes	*Describe the methods by which all novel plant genotypes were produced. This includes those generated by transgenic approaches, gene editing, chemical/radiation-based mutagenesis and hybridization. For transgenic lines, describe the transformation method, the number of independent lines analyzed and the generation upon which experiments were performed. For gene-edited lines, describe the editor used, the endogenous sequence targeted for editing, the targeting guide RNA sequence (if applicable) and how the editor was applied.*
Authentication	*Describe any authentication procedures for each seed stock used or novel genotype generated. Describe any experiments used to assess the effect of a mutation and, where applicable, how potential secondary effects (e.g. second site T-DNA insertions, mosiacism, off-target gene editing) were examined.*
